# Twenty-year trajectories of cardio-metabolic factors among people with type 2 diabetes by dementia status in England: a retrospective cohort study

**DOI:** 10.1007/s10654-023-00977-7

**Published:** 2023-03-04

**Authors:** Heidi T. M. Lai, Kiara Chang, Mansour T. A. Sharabiani, Jonathan Valabhji, Edward W. Gregg, Lefkos Middleton, Azeem Majeed, Jonathan Pearson-Stuttard, Christopher Millett, Alex Bottle, Eszter P. Vamos

**Affiliations:** 1 Public Health Policy Evaluation Unit, School of Public Health, Imperial College London, St Dunstan’s Road, London, W6 8RP UK; 2 Department of Primary Care and Public Health, School of Public Health, Imperial College London, London, UK; 3 NHS England, London, UK; 4 Department of Diabetes and Endocrinology, St Mary’s Hospital, Imperial College Healthcare NHS Trust, London, UK; 5 Division of Metabolism, Digestion and Reproduction, Imperial College London, London, UK; 6 School of Population Health, RCSI University of Medicine and Health Sciences, Dublin, Ireland; 7 Department of Epidemiology and Biostatistics, School of Public Health, Imperial College London, London, UK; 8 Ageing Epidemiology Research Unit, School of Public Health, Imperial College London, London, UK; 9 Imperial College Healthcare NHS Trust, London, UK; 10 Health Analytics, Lane Clark & Peacock LLP, London, UK; 11 Northumbria Healthcare NHS Foundation Trust, North Shields, UK; 12 Public Health Research Centre, Comprehensive Health Research Center, NOVA National School of Public Health, CHRC, NOVA University Lisbon, Lisbon, Portugal

**Keywords:** Cardio-metabolic risk factors, Vascular risk factors, Predictors, Blood pressure, Body mass index, Glucose, Haemoglobin A1c, Cholesterol, Low-density lipoprotein, High-density lipoprotein, Type 2 diabetes, Dementia, Longitudinal, Trajectories, Cohort

## Abstract

To assess 20-year retrospective trajectories of cardio-metabolic factors preceding dementia diagnosis among people with type 2 diabetes (T2D). We identified 227,145 people with T2D aged > 42 years between 1999 and 2018. Annual mean levels of eight routinely measured cardio-metabolic factors were extracted from the Clinical Practice Research Datalink. Multivariable multilevel piecewise and non-piecewise growth curve models assessed retrospective trajectories of cardio-metabolic factors by dementia status from up to 19 years preceding dementia diagnosis (dementia) or last contact with healthcare (no dementia). 23,546 patients developed dementia; mean (SD) follow-up was 10.0 (5.8) years. In the dementia group, mean systolic blood pressure increased 16–19 years before dementia diagnosis compared with patients without dementia, but declined more steeply from 16 years before diagnosis, while diastolic blood pressure generally declined at similar rates. Mean body mass index followed a steeper non-linear decline from 11 years before diagnosis in the dementia group. Mean blood lipid levels (total cholesterol, LDL, HDL) and glycaemic measures (fasting plasma glucose and HbA1c) were generally higher in the dementia group compared with those without dementia and followed similar patterns of change. However, absolute group differences were small. Differences in levels of cardio-metabolic factors were observed up to two decades prior to dementia diagnosis. Our findings suggest that a long follow-up is crucial to minimise reverse causation arising from changes in cardio-metabolic factors during preclinical dementia. Future investigations which address associations between cardiometabolic factors and dementia should account for potential non-linear relationships and consider the timeframe when measurements are taken.

**Supplementary Information:**

The online version contains supplementary material available at 10.1007/s10654-023-00977-7.

## Introduction

Type 2 diabetes and dementia are both increasing in prevalence, costly to health systems, and major contributors to comorbidities and mortality globally [1, 2]. People with type 2 diabetes have a two-fold increased dementia risk [3]. In England, one fifth of people aged ≥ 65 years with dementia have diabetes [4], leading to increasingly complex clinical and social care needs.

Despite dementia being a leading cause of death, our understanding of its risk factors and prevention has advanced slower than that for other major non-communicable diseases. Type 2 diabetes represents a potential intervention point, as up to 10% of dementia cases may be attributable to type 2 diabetes [5]. However, the causal pathways of diabetes-related dementia risk are not well-understood and effective preventive approaches are currently limited [5]. Several underlying mechanisms have been proposed including cerebral insulin dysregulation, cerebrovascular abnormalities, advanced protein glycation, oxidative stress and cerebral accumulation of several proteins [5–8]. These pathologies, however, can occur in the absence of diabetes and are known to progressively accumulate over several years before the clinical onset of cognitive decline and dementia. These early pre-clinical stages are acknowledged as potential targets for secondary prevention and future disease-modifying pharmaceutical therapies [6]. Although hyperglycaemia has been linked to increased dementia risk, intensified glycaemic control and diabetes-specific medical treatments have not shown to change the course of dementia development [8, 9].

Cardio-metabolic factors present as potentially modifiable risk factors in people with type 2 diabetes, as mid-life hypertension, obesity, physical inactivity, and dyslipidaemia each independently increase dementia risk in the general population [2, 10]. However, the levels of cardio-metabolic factors and their effects on dementia risk may vary over the lifespan, presenting challenges to identify targets for timely interventions. A few studies have explored cardio-metabolic factor trajectories within the general population [11–14]. Crucially, long-term retrospective trajectories of cardio-metabolic factors before dementia onset have not been described among populations with type 2 diabetes apart from one small study [15]. Generally, studies assessing links between cardio-metabolic factors and dementia in people with diabetes have been limited by study design, small sample size, short follow-up, and a limited range of study covariates and outcomes [15–22]. To address these knowledge gaps, this study aims to characterise and compare the 20-year retrospective trajectories of eight routinely measured cardio-metabolic factors leading up to dementia diagnosis among people with type 2 diabetes using data from the UK Clinical Practice Research Datalink in England.

## Methods

### Study design and population

We used data from the UK Clinical Practice Research Datalink (CPRD) ‘GOLD’, one of the largest electronic primary care database which holds longitudinal pseudo-anonymised electronic health records [23]. For a subset of English practices (n = 410), data linkage is available to Hospital Episode Statistics (HES) and Office for National Statistics (ONS) mortality files covering approximately 7% of the English population [24]. Individuals’ socio-demographic characteristics, clinical history and assessments, laboratory tests and prescriptions were collected prospectively during routine care [24]. Ethics approval was obtained from the Independent Scientific Advisory Committee for Medicines and Healthcare products Regulatory Agency under registered study protocol number 16_252. We extracted data from 1st January 1999 to 31st December 2018 for people with type 2 diabetes.

### Type 2 diabetes ascertainment

We established our analytical cohort by identifying individuals with type 2 diabetes using diagnostic (C10) and management (66A) ‘Read codes’, prescription data for glucose lowering therapy, and ICD-10 diagnostic codes in HES and ONS records (Supplementary Tables 1–3). We included people who were diagnosed with type 2 diabetes at any time between 1st January 1999 and 31st December 2018. We did not include people diagnosed with diabetes ≤ 35 years of age who were prescribed insulin within 6 months of diagnosis without receiving oral hypoglycaemic medications for > 6 months as part of our definition, as they were likely to have type 1 diabetes.

### Dementia ascertainment

Incident dementia was identified using an algorithm (Supplementary Fig. 1) including combinations of clinical diagnostic, administrative, cognitive functioning testing, prescription, hospital admissions, referrals, and mortality data to maximise the detection of dementia cases, because dementia is known to be under-recorded in electronic health records. Dementia codes in CPRD have been previously validated [25], and are listed in Supplementary Tables 4–6. For the main dementia definition, we firstly identified diagnostic codes and prescription of dementia drugs from primary care records, and diagnostic codes on admissions in HES data and ONS mortality files in any diagnostic field. We then maximised detection rates by including valid results indicative of dementia from cognitive tests, including Mini-Mental State Examination, General Practitioner Assessment of Cognition, Six-item Cognitive Impairment Test, Abbreviated Mental Test, and Addenbrooke’s Cognitive Examination.

To further maximise case detection, we identified people with diagnostic and administrative management codes that refer to mild/moderate cognitive impairment, memory loss or referral to memory clinic and classified people as probable cognitive impairment cases if they also had a cognitive testing recorded without a valid result. This resulted in 519 probable cognitive impairment cases in addition to 23,546 from the main definition, resulting in a total of 24,065 cases. We also identified 697 people with referral to memory clinic (possible cognitive impairment cases), which increased the total number of dementia cases to 24,243.

### Final analytical population

We identified 351,428 eligible people, and excluded 91,267 who were born after 1957 (aged < 42 years old in 1999, as dementia mostly affect elderly individuals, and risk of dementia increases with age), 17,685 who did not meet CPRD quality assurance criteria for research use [24], 7471 who did not receive a diabetes diagnosis within their valid follow-up times, 7858 who received a dementia diagnosis before or at the beginning of their follow-up, and two who reported indeterminate gender. As such, 227,145 people aged 42 years old or older who developed type 2 diabetes at any time during follow-up (i.e., after the index year of 1999) were included in the final sample (Supplementary Fig. 2). All were followed up until dementia diagnosis, death, transfer out-of-practice or end of study period, whichever occurred first.

### Cardio-metabolic measurements

Cardiometabolic factors were measured according to standardised protocols during routine primary care visits [24]. Systolic blood pressure (SBP), diastolic blood pressure (DBP), body mass index (BMI), fasting plasma glucose (FPG), haemoglobin A1c (HbA1c), total cholesterol, high-density lipoprotein (HDL), and low-density lipoprotein (LDL) measurements were extracted and averaged as annual means for each individual. All measurements (including those taken before diabetes diagnosis) throughout follow-up were included. As the management of chronic diseases and recording of care processes have been financially incentivised in English primary care since 2004, there is a reasonably robust annual ascertainment of these parameters [26].

### Other covariates

Study covariates included baseline age, sex, ethnicity, smoking status, Index of Multiple Deprivation (IMD) quintiles [27], duration of diabetes, time-varying insulin, anti-hyperglycaemic, anti-hypertensive, anti-lipid, anti-platelet medications, and the number of co-morbid conditions. Detailed definitions can be found in supplementary methods.

### Statistical analysis

People with type 2 diabetes were divided into two groups based on whether they developed dementia at any point during follow-up. The date of dementia diagnosis (people with dementia) or last contact with their healthcare (people without dementia) is designated as the zero time-point. From the zero time-point, all people were traced backwards in time retrospectively to their first contact with healthcare, the earliest of which is 1st January 1999.

Annual means of each cardio-metabolic factors were first calculated for each individual and their duration of follow-up years. Multilevel linear or non-linear growth curve models (spline regression with individual-specific random intercept and slope) assessed the differences in longitudinal retrospective trajectories of cardio-metabolic factors between people with and without dementia. The incorporation of the spline function helped capture potential changes in trajectories of cardio-metabolic factors. Therefore, follow-up time was either treated as a single time period (non-piecewise) or segmented into two or more fragments (piecewise).

Choice and number of time segments was initially based on the visual inspection of cohort retrospective trajectories of cardio-metabolic factors plotted as a function of time (Supplementary Figs. 3–5). This was further optimised by testing incorporated time segments with different choices of nearby knots or simpler models with fewer or no knots (linear model). Higher-order polynomial of time segment(s) was also considered whenever appropriate. The best fitting model was determined using likelihood ratio tests for comparison between nested models, and by examining the Akaike Information Criterion (AIC) and Bayesian Information Criterion (BIC) between non-nested models. The most parsimonious model with the lowest AIC and BIC, as well as a likelihood ratio test result of *p < *0.05 when compared to the previous nested model iterations was considered superior and chosen for further analyses. As such, growth models for HDL and total cholesterol were non-piecewise (one single time segment); BMI, FPG and HbA1c were piecewise with two time segments; SBP, DBP and LDL with three time segments. Model equations are presented in supplementary materials (Eqs. 1–4).

Dementia status was modelled as a fixed effect, and its interactions with time examines the departure of outcome trajectories among people without dementia (reference) from those with dementia. For random slope effects, only the time segment closest to the zero time-point was included, as including two or more time segments resulted in non-convergence. Minimal adjustments included age, sex, and their interactions with time (fixed effects). The final models were additionally adjusted for (i) ethnicity at the zero time-point, smoking status, IMD quintiles, duration of diabetes, and their interactions with time, and (ii) time-varying insulin prescription and number of comorbid conditions.

For sensitivity analyses, we firstly assessed the robustness of our dementia definition by including probable and possible cognitive impairment cases as exposures separately. Second, we restricted the analyses to people with ≥ 10 years of follow-up to assess robustness against follow-up time. Third, additional individual adjustments for stroke and acute myocardial infarction, which are important potential confounders, in place of the number of comorbid conditions assessed the impacts of adjusting for major cardiovascular events in a time-varying manner. Fourth, we assessed the further inclusion of time-varying anti-hypertensive, oral anti-hyperglycaemic, anti-lipid, and anti-platelet medications. Lastly, we repeated the analyses using a case-control approach: matching two controls per person with dementia without replacement by age at zero time-point (± 3 years) and exactly by sex.

All statistical analyses were performed in Stata 16.1; a two-sided *p* < 0.05 was considered statistically significant.

## Results

### Cohort characteristics

Among 227,145 people with type 2 diabetes, there were 23,546 incident dementia cases (Table 1). The mean (SD) follow-up time was 9.6 (5.8) years for people with dementia and 10.0 (5.9) years for people without dementia. People with dementia were older, more likely to be white, male, non-smokers with a longer diabetes duration, and had more comorbid conditions compared with people without dementia. The two groups were similar regarding ever receiving insulin treatment and socioeconomic status.

**Table 1:** Characteristics of study participants with type 2 diabetes in the Clinical Research Practice Datalink at the time of dementia diagnosis (dementia group) or last contact with healthcare (non-dementia group) between 1999 and 2018 in England

	Total (n = 227,145)	Dementia (n = 23,546)	Non-dementia (n = 203,599)	*p*-value^*^
Age, years, mean (SD)	73.4 (10.2)	82.2 (8.1)	72.4 (10.0)	< 0.001
*Sex, n (%)*				
Male	119,561 (52.6)	10,157 (43.1)	109,404 (53.7)	< 0.001
Female	107,584 (47.4)	13,389 (56.9)	94,195 (46.3)	
*Ethnicity, n (%)*				
White	194,069 (85.4)	21,606 (91.8)	172,463 (84.7)	< 0.001
Non-White	19,296 (8.5)	1392 (5.9)	17,904 (8.8)	
Unknown/missing	13,780 (6.1)	548 (2.3)	13,232 (6.5)	
*Smoking status, n (%)*				
Non-smoker	86,213 (38.0)	9968 (42.3)	76,245 (37.4)	< 0.001
Smoker	70,084 (30.9)	5407 (23.0)	64,677 (31.8)	
Ex-smoker	70,848 (31.2)	8171 (34.7)	62,677 (30.8)	
*Index of Multiple Deprivation (IMD) quintiles, n (%)*				
1st quintile (least deprived)	32,377 (14.3)	3264 (13.8)	29,113 (14.3)	0.190
2	43,277 (19.1)	4480 (19.0)	38,797 (19.1)	
3	44,092 (19.4)	4520 (19.2)	39,572 (19.4)	
4	50,778 (22.4)	5367 (22.8)	45,411 (22.3)	
5th quintile (most deprived)	56,621 (24.9)	5915 (25.1)	50,706 (24.9)	
Diabetes duration, years, mean (SD)	6.9 (5.4)	8.2 (6.0)	6.7 (5.4)	< 0.001
Insulin, ever prescribed, n (%)	25,328 (11.2)	2558 (10.9)	22,770 (11.2)	0.201
Number of co-morbidities, median (IQR)^†^	1 (0–2)	2 (1–3)	1 (0–2)	< 0.001
Anti-hyperglycaemic, ever prescribed, n (%)	134,258 (59.1)	13,341 (56.7)	120,917 (59.4)	< 0.001
Anti-hypertensive, ever prescribed, n (%)	169,002 (74.4)	17,918 (76.1)	151,084 (74.2)	< 0.001
Anti-lipid, ever prescribed, n (%)	148,760 (65.5)	14,883 (63.2)	133,877 (65.8)	< 0.001
Anti-platelet, ever prescribed, n (%)	97,088 (42.7)	11,733 (49.8)	85,355 (41.9)	< 0.001
Retrospective follow-up time, years, mean (SD)	10.0 (5.8)	9.6 (5.8)	10.0 (5.9)	< 0.001 ara>

*Comparisons were made between dementia and non-dementia groups, with *p*-value obtained through two-sample independent *t*-test for continuous variables and chi-squared test for categorical variables

†Comorbidities include stroke, acute myocardial infarction, peripheral artery disease, atrial fibrillation, heart failure, asthma, chronic obstructive pulmonary disease, cancer, chronic kidney disease, rheumatoid arthritis, Parkinson’s disease, and clinical depression

### Blood pressure levels and body mass index

**Fig. 1:**
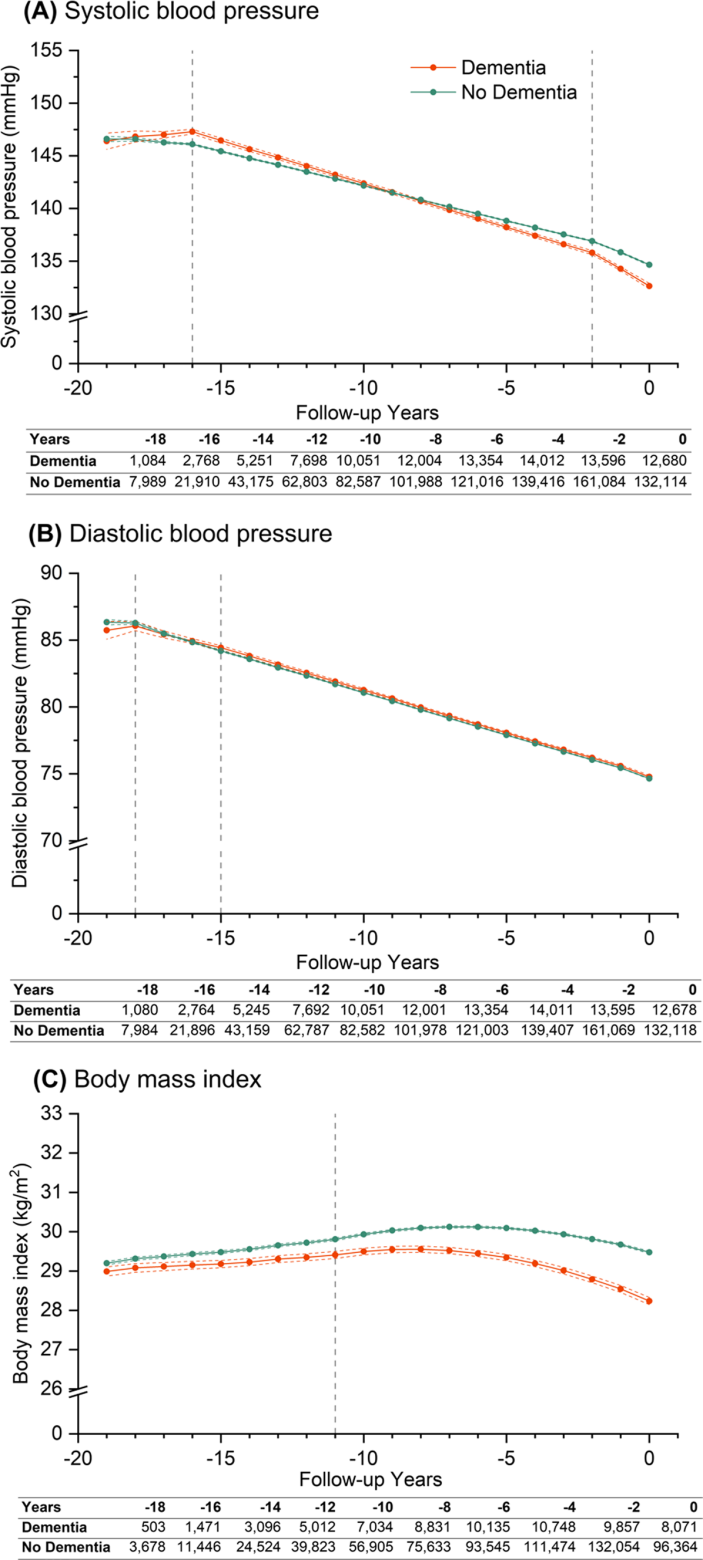
Trajectories of (**A**) systolic blood pressure, (**B**) diastolic blood pressure, and (**C**) body mass index over 20 years of retrospective follow-up. Year 0 (zero time point) represents either the date of diagnosis of dementia (dementia group) or last contact with healthcare (non-dementia group). Patients were traced backwards in time, and were allowed to enter the cohort at any time conditional on a diabetes diagnosis within their retrospective follow-up duration. Estimations are based on piecewise linear (systolic and diastolic blood pressure) and non-linear (body mass index) growth curve models, including up to three time periods modelled as linear or non-linear splines, dementia status, and interactions between dementia status and time. Coloured solid lines represent point estimates for each group, coloured dotted lines for 95% confidence intervals for said estimations, while modelled time periods are indicted by grey vertical dotted lines. Three time periods ranging from year 0 to − 2, − 2 to − 16, − 16 to − 19 (from right to left) were defined for systolic blood pressure; from year 0 to − 15, − 15 to − 18, − 18 to − 19 for diastolic blood pressure; and two time periods ranging from year 0 to − 11 and − 11 to − 19 were defined for body mass index. Retrospective trajectories are adjusted for covariates defined at year 0, including age, sex, ethnicity, smoking status, Index of Multiple Deprivation quintiles, duration of diabetes and their interactions with time, and time-varying covariates including insulin prescription and number of comorbid conditions. Tables underneath each graph report the number of measurements for dementia and non-dementia group at each stated retrospective follow-up year, where year 0 is the study baseline

In the fully adjusted models, trajectories of mean SBP generally followed a downward trend from the point of first contact regardless of dementia status (Fig. 1). However, annual rates of decline varied throughout (Supplementary Table 8). Mean SBP levels were similar between groups 19 years prior, at 146.4 (95% CI 145.6–147.2) mmHg and 146.6 (146.4–146.8) for people with and without dementia respectively (Fig. 1). Moving forward in time, mean SBP levels were initially higher for people with dementia vs. without between 17 and 10 years before diagnosis by 0.21 (0.02–0.39) to 1.19 (0.94–1.43) mmHg (Supplementary Table 9). Between seven to two years before diagnosis, mean SBP levels among people with dementia were lower than people without dementia by − 0.28 (− 0.46 to − 0.11) to − 1.10 (− 1.29 to − 0.90) mmHg (Supplementary Table 9). By diagnosis, people with dementia had a lower mean SBP [132.6 (132.4–132.9) mmHg] versus people without dementia [134.7 (134.6–134.7) mmHg], with a statistically significant difference of − 2.01 (− 2.28 to − 1.74) mmHg (Supplementary Table 9). These group differences are reflected in the annual rates of change in SBP levels for people with dementia relative to the non-dementia group: 0.47 (0.19–0.74) mmHg/year between − 19 and − 16 years; − 0.16 (− 0.18 to − 0.14) mmHg/year greater reductions between − 16 and − 2 years; and − 0.46 (− 0.60 to − 0.31) mmHg/year in the final two years before diagnosis (Supplementary Table 8). DBP declined steadily over retrospective follow-up in both dementia and non-dementia groups; differences between groups were statistically significant, but small in absolute terms (Supplementary Table 9).

People with dementia had a consistently lower mean BMI compared with people without dementia [− 0.21 (− 0.33 to − 0.09) kg/m^2^ to − 1.24 (− 1.34 to − 1.14) kg/m^2^, *p* < 0.002] throughout the lead up to diagnosis (Supplementary Table 9). Mean BMI was lower for the dementia group 19 years before diagnosis: 29.0 (28.9–29.1) kg/m^2^; and slightly higher in the non-dementia group at 29.2 (29.1–29.2) kg/m^2^ (Fig. 1). Although mean BMI levels initially increased in both groups during the first decade, albeit by a lesser extent among people with dementia [0.023 (− 0.035 to 0.011) kg/m^2^] (Supplementary Table 8), both trajectories followed a concave downward trend thereafter in the latter decade towards the zero time-point (Fig. 1), where people with dementia experienced a significantly steeper decline, resulting in a lower BMI. By diagnosis, BMI was 28.2 (28.1–28.3) kg/m^2^ for people with dementia vs. 29.5 (29.5–29.5) kg/m^2^ for people without dementia.

### Glucose and HbA1c

**Fig. 2:**
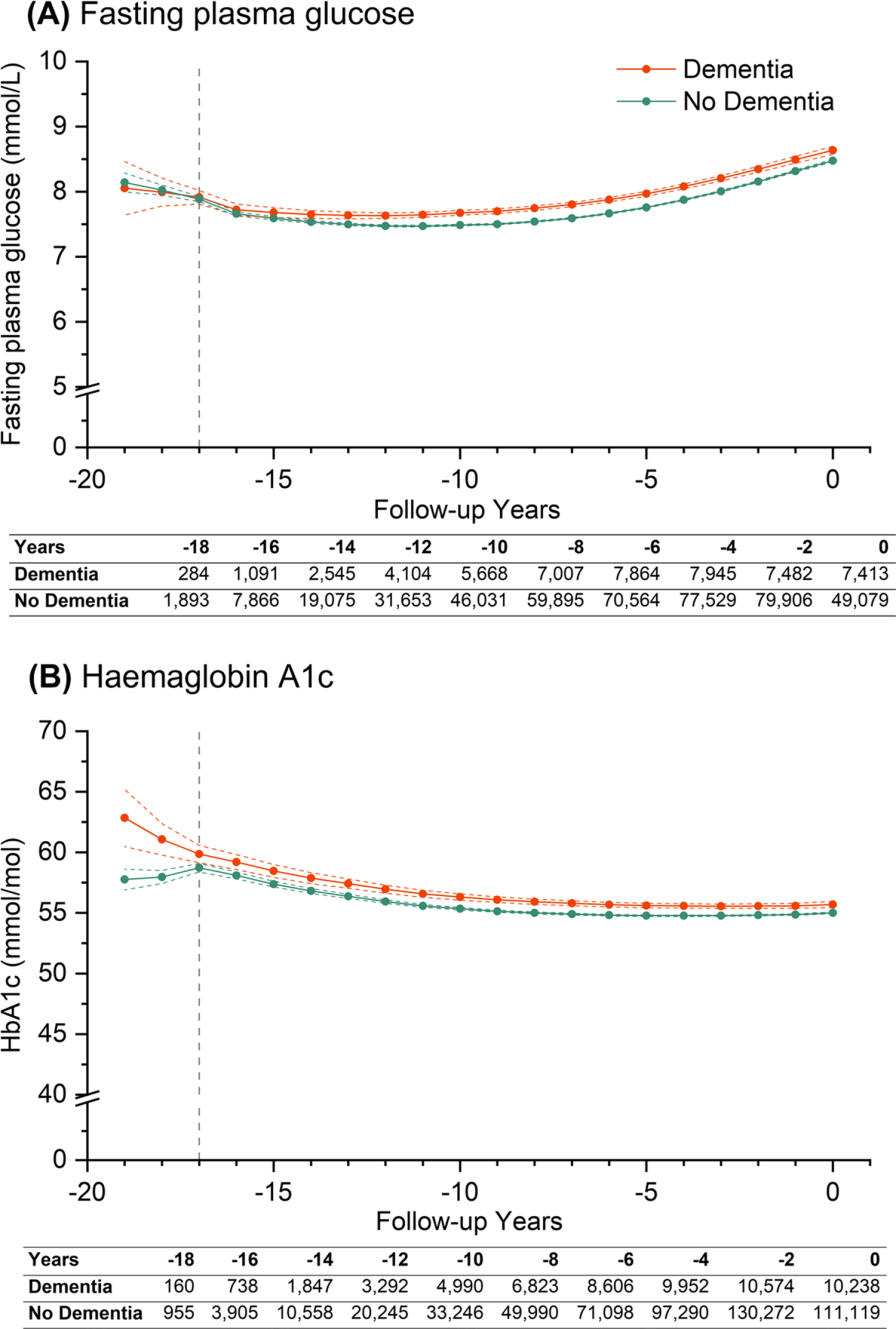
Trajectories of (**A**) fasting plasma glucose and (**B**) HbA1c over 20 years of retrospective follow-up. Year 0 (zero time point) represents either the date of diagnosis of dementia (dementia group) or last contact with healthcare (non-dementia group). Patients were traced backwards in time, and were allowed to enter the cohort at any time conditional on a diabetes diagnosis within their retrospective follow-up duration. Estimations are based on piecewise non-linear growth curve models, including two time periods modelled as linear or non-linear splines, dementia status, and interactions between dementia status and time. Coloured solid lines represent point estimates for each group, coloured dotted lines for 95% confidence intervals for said estimations, while modelled time periods are indicted by grey vertical dotted lines. The two time periods ranging from year 0 to − 17 and − 17 to − 19 (from right to left) were defined for fasting plasma glucose and haemaglobin A1c. Retrospective trajectories are adjusted for baseline covariates including age, sex, ethnicity, smoking status, Index of Multiple Deprivation quintiles, duration of diabetes and their interactions with time, and time-varying covariates including insulin prescription and number of comorbid conditions. Tables underneath each graph report the number of measurements for dementia and non-dementia group at each stated retrospective follow-up year, where year 0 is the study baseline

Mean levels of FPG and HbA1c were generally higher among people with dementia throughout follow-up (Fig. 2). At 19 years before zero time-point, mean FPG levels were lower in both groups at 8.05 (7.64–8.46) mmol/L for people with dementia and 8.14 (8.00–8.29) mmol/L for people without dementia (Fig. 2). Moving forward in time, trajectories of FPG were initially similar between groups, but followed a U-shaped trend upwards, which resulted in higher FPG levels at zero time-point. Mean differences in levels of FPG ranged from 0.09 (0.01–0.17) mmol/L to 0.21 (0.17–0.25) mmol/L (*p* < 0.03) in the last 15 years of follow-up (Supplementary Table 9). At dementia diagnosis, mean FPG was higher at 8.64 (8.58–8.70) mmol/L versus 8.48 (8.46–8.50) mmol/L for people without dementia.

Mean levels of HbA1c among people with dementia were 5 mmol/mol higher (*p* < 0.001) 19 years before zero time-point [63 (60–65) mmol/mol] compared with people without dementia [58 (57–59) mmol/mol]. People with dementia experienced a − 2 (− 3 to − 1) mmol/mol greater reduction per year vs. people without dementia between 19 and 17 years before diagnosis (Supplementary Table 8). HbA1c trajectories followed a non-linear trend in both groups, where estimated HbA1c levels remained higher among people with dementia relative to people without dementia. However, overall mean differences only reduced by about 1 mmol/mol in the latter 16 years of follow-up (*p* < 0.002) (Supplementary Table 9). At diagnosis, mean HbA1c levels were 56 (55–57) mmol/mol versus 55 (55–55) mmol/mol for people with dementia versus without respectively.

### Lipids

**Fig. 3:**
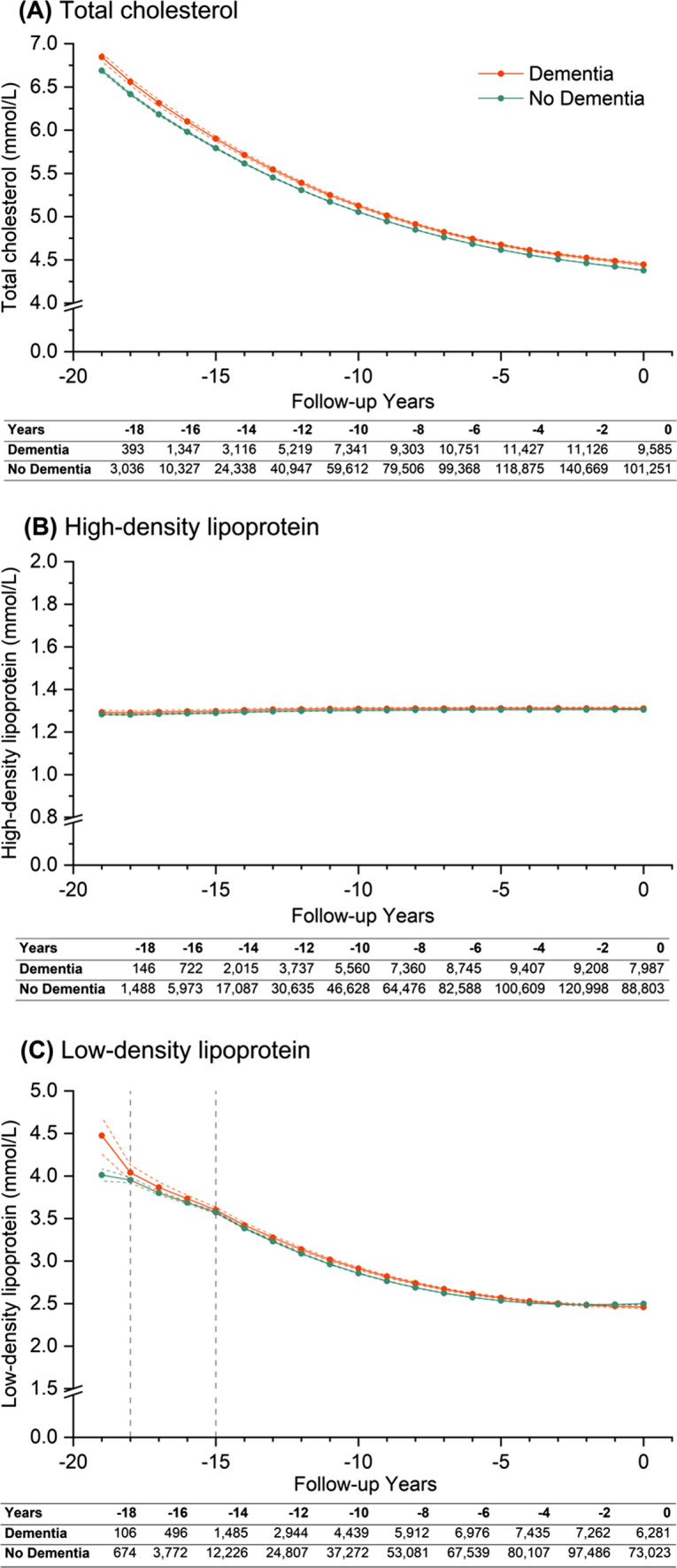
Trajectories of (**A**) cholesterol, (**B**) high density lipoprotein, and (**C**) low density lipoprotein over 20 years of retrospective follow-up. Year 0 (zero time point) represents either the date of diagnosis of dementia (dementia group) or last contact with healthcare (non-dementia group). Patients were traced backwards in time, and were allowed to enter the cohort at any time conditional on a diabetes diagnosis within their retrospective follow-up duration. Estimations are based on non-piecewise linear (high-density lipoprotein) or non-linear (total cholesterol), or piecewise non-linear (low-density lipoprotein) growth curve models, including up to three time periods modelled as linear or non-linear splines, dementia status, and interactions between dementia status and time. Coloured solid lines represent point estimates for each group, coloured dotted lines for 95% confidence intervals for said estimations, while modelled time periods are indicted by grey vertical dotted lines. Both total cholesterol and high-density lipoprotein considered time as a single period from 0 to -19 (from right to left), while three time periods ranging from year 0 to − 15, − 15 to − 18, − 18 to − 19 were defined for low-density lipoprotein. Retrospective trajectories are adjusted for baseline covariates including age, sex, ethnicity, smoking status, Index of Multiple Deprivation quintiles, duration of diabetes and their interactions with time, and time-varying covariates including insulin prescription and number of comorbid conditions. Tables underneath each graph report the number of measurements for dementia and non-dementia group at each stated retrospective follow-up year, where year 0 is the study baseline

Overall, differences in mean levels of cholesterol, HDL, and LDL were small between groups, following largely similar trajectories (Supplementary Table 8). People with dementia generally had higher levels of all lipids during follow-up (Fig. 3 and Supplementary Table 10).

Mean levels of total cholesterol followed a non-linear downwards trajectory from 6.85 (6.80–6.89) mmol/L 19 years before to 4.45 (4.43–4.46) mmol/L at zero time-point among people with dementia, versus 6.69 (6.67–6.71) mmol/L 19 years before to 4.38 (4.37–4.38) mmol/L at zero time-point for people without dementia. Although differences between groups were small, it remained statistically significant throughout follow-up (*p* < 0.001) (Supplementary Table 10). Mean levels of HDL followed a linear trend during follow-up in both groups without significant changes.

Estimated mean LDL at zero time-point for people with and without dementia was 2.46 (2.44–2.48) mmol/L and 2.50 (2.49– 2.50) mmol/L, as opposed to 4.48 (4.26–4.69) mmol/L and 4.01 (3.94–4.08) mmol/L, respectively, 19 years prior. Annual decline in mean LDL levels was steeper by 0.38 (0.62–0.14) mmol/L per year among people with dementia 19–18 years before zero time-point, but differences in decline did not statistically differ between the two groups between 18 and 15 years before zero time-point and followed a non-linear downward trend in the latter 15 years of follow-up.

### Sensitivity analyses

Sensitivity analysis including probable and possible cognitive impairment cases; and the restriction of analyses to people with ≥ 10 years of follow-up did not considerably alter findings (Supplementary Tables 11–15). Cohort characteristics using the age- and sex- matched case-control approach were reported in Supplementary Table 16. Findings using this approach were generally similar to the main analyses with minor shifts in estimates (Supplementary Tables 17–19 and Fig. 6). However, the annual rate of change in SBP mmHg/year was smaller in the earliest period of follow-up: case-control approach: 0.27 (0.17–0.71) mmHg versus main approach: 0.47 (0.19–0.74) mmHg (Supplementary Table 17).

## Discussion

In this large cohort study, we observed differences in cardio-metabolic factors in people with type 2 diabetes in England by dementia status over a 20-year study period. People with type 2 diabetes who developed dementia had higher levels of SBP, BMI, and LDL 19 years before dementia diagnosis that decreased more steeply leading up to diagnosis, resulting in lower levels at the time of dementia diagnosis. Conversely, historical glycaemic measures and total cholesterol levels remained consistently higher among people with dementia, albeit with minor absolute differences, compared with people without dementia after adjustment for several key confounders.

Previous research assessing long-term trajectories of cardio-metabolic factors in type 2 diabetes leading up to dementia onset is scarce. The Hoorn study (n = 64) showed higher SBP, similar weight and lipid measures, and lower HbA1c among people with type 2 diabetes, associated with poor cognition during a 16-year follow-up [15]. Our study expands upon these findings by utilising a larger sample (hence larger statistical power), including more repeated measurements, and ascertaining dementia using multiple sources (vs. cognitive tests only). Importantly, our models were adjusted for age and several key confounders which were not considered in prior studies. Our findings were also robust to an age-and-sex-matched case-control approach and further adjustments for medications.

Previous trials had not reported benefits for cognition with intensive glucose lowering [28], blood pressure and lipid lowering [29], or individualised lifestyle interventions, including weight loss [30, 31]. Furthermore, the ACCORDIAN MIND study reported no long-term benefits for cognitive function with intensive vs. standard treatments for blood pressure, glycaemia, or lipids among people with type 2 diabetes [32]. Crucially, many previous observational studies examining modifiable diabetes-related risk factors for dementia were cross-sectional, lacked substantive follow-up data and had a limited range of study variables, and generally provided conflicting results [16]. While midlife hypertension, dyslipidaemia, and obesity have been associated with late-life dementia in the general population, the role of these factors in influencing excess dementia risk in people with diabetes is not well-understood [16]. Associations between hypertension and subsequent cognitive dysfunction in diabetes have been reported [15–17], but others failed to demonstrate similar findings [18] or even found protective effects of hypertension [19]. Prior findings for associations between dyslipidaemia and cognitive decline in people with diabetes were also inconsistent, with some studies linking hypercholesterinaemia to a decreased risk of cognitive decline [15, 16, 21]. Furthermore, epidemiological studies suggest associations between mid-life obesity and central adiposity and diabetes-related cognitive dysfunction, but late-life associations remain uncertain [5, 15, 20, 21]. Our findings, however, agree with several studies that showed higher glycaemic measures associated with dementia [16, 22].

There are several explanations for the overall downward trends in blood pressure, BMI, cholesterol, and LDL. Firstly, in line with our findings, blood pressure, weight, and cholesterol have been shown to reduce with an increasing age from mid- to late-life in the general population, with more pronounced reductions associated with cognitive decline [11–14, 33]. Secondly, these trends may also reflect general improvements in medication use over time, given how trajectories in both groups were not appreciably different, e.g. increase in statin use. However, evolving group differences in the latter decade, i.e., lower levels of SBP, BMI, LDL, may be attributed to reverse causation, driven by the onset and progression of prodromal dementia. High SBP at midlife could be due to vascular endothelial dysfunction, which may lead to cerebral hypoperfusion hypoxia and other brain injuries, contributing to cognitive decline preceding dementia diagnosis [34]. Accelerated decline in BMI among people with dementia could be a manifestation of wasting, associated with reduced olfactory function, predementia apathy, loss of initiative, difficulty in eating, and malnutrition associated with dementia [35–37]. However, unlike findings from the general population, we found lower levels of BMI in the group who developed dementia throughout the 20-year follow-up. It is possible that the role of BMI in dementia development in type 2 diabetes is less defining than that in the general population, given how BMI is already a predictor of type 2 diabetes. While the role of cholesterol remains inconclusive [38], genetic factors such as ApoE4, and established mechanisms such as atherosclerosis may play a role in the development of microvascular and macrovascular disease, a known risk factor for dementia [19, 39]. Altogether, these proposed mechanisms lend support to our findings, and may be associated with accelerated decline in levels of SBP, BMI, and LDL in the years leading to dementia diagnosis.

Consistently higher levels of glycaemic measures among people with dementia vs. without dementia after adjustment for several confounders (e.g. diabetes duration and time-varying microvascular disease) may reflect the effects of cumulative exposures [22]. Biological mechanisms which support this include worsening insulin resistance, likely associated with abnormalities in insulin signalling pathways affecting systemic metabolism and cerebral insulin signalling [34], and possibly microvascular disease, a known risk factor of diabetes and dementia [39]. Although reported differences were statistically significant, the absolute differences were small. Furthermore, randomised clinical trials did not find sufficient evidence to support the prevention or delay in cognitive dysfunction with intensive glycaemic control over standard treatments [9, 28]. As a result, further evidence is needed to support interventions altering glycaemic measures among people with type 2 diabetes to prevent or delay dementia, given small observed differences between groups.

Our findings suggest that cardiometabolic measurements taken within a decade of dementia diagnosis are likely to reflect reverse causation, acting as markers of dementia development, rather than risk factors. Potential non-linear associations, such as those observed for BMI and LDL, should also be considered. Based on the poorer cardiometabolic profile at mid-life among people with dementia, our findings also highlight that people who develop dementia are likely to have undergone changes in cardiometabolic levels which differ from people who do not develop dementia. It is possible that these changes are a result of cumulative long-term exposure. Future studies which aim to investigate causal relationships between cardiometabolic factors and dementia should consider accounting for potential non-linear associations, as well as the timeframe when measurements are taken, as measurements taken later in mid-life or late life would limit its ability to assess dementia risk.

To our knowledge, this is the first large-scale study to characterise long-term retrospective trajectories of cardio-metabolic factors before dementia onset in people with type 2 diabetes. Strengths include using a large type 2 diabetes population-based CPRD cohort, which is broadly representative of the English population [24], availability of key laboratory and clinical parameters, and two decades of follow-up.

However, several limitations need to be considered. Routinely collected data are subject to missingness and changing recording practices potentially resulting in increasing non-representativeness over time. However, our study includes an unselected population from the CPRD covering up to 8% of the English population, and patients who newly met eligibility criteria during the 20-year follow-up were included in the cohort. We were unable to assess the extent of misclassification due to miscoding or undiagnosed cases, as well as missed dementia cases in individuals with severe cognitive decline who relocated to care homes due to incipient dementia. However, the inclusion of probable and possible cognitive impairment cases in our study aim to maximise case detection by identifying people who are lost to follow-up before the recording of a clinical diagnosis. CPRD data is also widely used for research with well-documented accuracy and completeness, and the implementation of recommended quality indicators ensures improved selection for research-quality data [24]. Validity of dementia codes in CPRD have also been established [25]. Dementia is known to be under-diagnosed [40], but we implemented an algorithm using diagnostic and administrative data, cognitive functioning testing, prescriptions, hospitalisation and mortality data to maximise case detection and subsequently tested the robustness of our findings by incorporating probable and possible cognitive impairment cases in sensitivity analyses. Residual presence of potential misclassification would likely underestimate group differences. Our study design does not allow accounting for the competing risk of dying from other causes before developing dementia. Changes in individuals’ cardio-metabolic parameters over time (e.g., increasing or decreasing blood pressure levels) may affect the risk of both all-cause deaths and dementia development [41, 42], and these associations could be moderated by patient characteristics such as age. Furthermore, our analyses did not specifically explore different patterns of change within the dementia and non-dementia groups in this population. However, the application of the multilevel growth curve model with individual-specific random intercept and slope allowed us to account for individual trends and non-linear relationships. Changes in treatment guidelines and quality of care may have influenced underlying trends in cardio-metabolic factors, which was likely to affect both groups given the similar patterns of change over time. However, we adjusted our analyses for calendar years and time-varying medications in sensitivity analyses. Age is a crucial confounder for dementia and the large age differences between dementia and non-dementia groups may contribute to bias. However, our findings are largely robust to a case-control sensitivity analysis, matched by age and sex. Despite adjusting for key confounders, residual confounding due to important confounders unavailable in routine care data, such as diet and physical activity, as well as the presence of other unknown factors may have affected the findings. Lastly, less than half of the study participants were middle-aged (45 years to 65 years), with the mean age ranging from 57 to 65 years for 19 to 10 study years, respectively. While the study period spans over two decades, a longer retrospective follow-up is needed to capture exposures before and throughout mid-life.

In a large cohort of people with type 2 diabetes followed up over two decades, differences in levels of modifiable cardio-metabolic factors can be observed between people who developed dementia and those who did not throughout follow-up. Although these findings do not imply causal associations, they highlight the importance of a long follow-up to minimise reverse causation, accounting for potential non-linear relationships, and the timeframe when measurements are taken to consider future exploration in the risk of dementia within this high-risk population.

## Electronic supplementary material

Below is the link to the electronic supplementary material.

Supplementary Material 1 (DOCX 2590KB)
