# Supplementary material for: Twenty-year trajectories of cardio-metabolic factors among people with type 2 diabetes by dementia status in England: a retrospective cohort study
Source: Eur J Epidemiol. 2023 Mar 4;38(7):733–44. doi: 10.1007/s10654-023-00977-7 (PMC10276060; doi:10.1007/s10654-023-00977-7)
Supplement: Supplementary file 1 — Supplementary Material 1 (DOCX 2590KB) [file 10654_2023_977_MOESM1_ESM.docx]

Supplementary Materials

**Listed in the order of appearance**

**Supplementary Methods**

*Covariate definitions*

Age was modelled continuously, centred at 82 years old (mean age for dementia group). Sex included male and female. Ethnicity (white, non-white, missing) was determined from the combination of CPRD and HES records (Supplementary Table 7). If ethnicity could not be determined from both CRPD and HES data, we assigned people to the ‘missing/unknown’ category (6%). Smoking status (smoker, ex-smoker, non-smoker) was defined using Read codes recorded closest to the date of dementia diagnosis (people with dementia) or last contact with healthcare. Index of Multiple Deprivation was mapped using general practice postcodes and provided in quintiles (1). This is a relative measure of neighbourhood deprivation derived for each small area of England based on seven indicators. Duration of diabetes was calculated from the first diagnosis of type 2 diabetes until last contact with healthcare or dementia diagnosis. Medications, including anti-hyperglycaemic (defined as insulin and oral anti-hyperglycaemic medications), anti-hypertensive, anti-lipid, anti-platelet medications were coded as binary time-varying variables. Co-morbid conditions were modelled as a continuous time-varying variable and were defined as the total number of comorbidities, including stroke, acute myocardial infarction, peripheral artery disease, atrial fibrillation, heart failure, asthma, chronic lung disease, malignancies, chronic kidney disease, retinopathy, rheumatoid arthritis, Parkinson’s disease, and depression, all defined based on individual’s history from a combination of primary and secondary care data.

**Supplementary Table 1.**

**Code list for the ascertainment of Type 2 diabetes, non-specific diabetes, and Type 1 diabetes in the Clinical Practice Research Datalink**

| **Type 2 Diabetes** |  |
| --- | --- |
| **Read codes** | **Description** |
| 66A3.00 | Diabetic on diet only |
| 66A4.00 | Diabetic on oral treatment |
| 66A4.00 | Diabetic on oral treatment |
| 66Ao.00 | Diabetes type 2 review |
| 66At100 | Type II diabetic dietary review |
| 66At111 | Type 2 diabetic dietary review |
| 66AV.00 | Diabetic on insulin and oral treatment |
| 8Hj4.00 | Referral to DESMOND diabetes structured education programme |
| 9NiD.00 | Did not attend DESMOND diabetes structured education program |
| 9OLK.00 | DESMOND diabetes structured education programme completed |
| C100100 | Diabetes mellitus, adult onset, no mention of complication |
| C100112 | Non-insulin dependent diabetes mellitus |
| C101100 | Diabetes mellitus, adult onset, with ketoacidosis |
| C102.00 | Diabetes mellitus with hyperosmolar coma |
| C102100 | Diabetes mellitus, adult onset, with hyperosmolar coma |
| C104100 | Diabetes mellitus, adult onset, with renal manifestation |
| C105100 | Diabetes mellitus, adult onset, + ophthalmic manifestation |
| C106100 | Diabetes mellitus, adult onset, + neurological manifestation |
| C107.00 | Diabetes mellitus with peripheral circulatory disorder |
| C107100 | Diabetes mellitus, adult, + peripheral circulatory disorder |
| C107200 | Diabetes mellitus, adult with gangrene |
| C107400 | NIDDM with peripheral circulatory disorder |
| C109.00 | Non-insulin dependent diabetes mellitus |
| C109.11 | NIDDM - Non-insulin dependent diabetes mellitus |
| C109.12 | Type 2 diabetes mellitus |
| C109.13 | Type II diabetes mellitus |
| C109000 | Non-insulin-dependent diabetes mellitus with renal comps |
| C109011 | Type II diabetes mellitus with renal complications |
| C109012 | Type 2 diabetes mellitus with renal complications |
| C109100 | Non-insulin-dependent diabetes mellitus with ophthalm comps |
| C109111 | Type II diabetes mellitus with ophthalmic complications |
| C109112 | Type 2 diabetes mellitus with ophthalmic complications |
| C109200 | Non-insulin-dependent diabetes mellitus with neuro comps |
| C109211 | Type II diabetes mellitus with neurological complications |
| C109212 | Type 2 diabetes mellitus with neurological complications |
| C109300 | Non-insulin-dependent diabetes mellitus with multiple comps |
| C109311 | Type II diabetes mellitus with multiple complications |
| C109312 | Type 2 diabetes mellitus with multiple complications |
| C109400 | Non-insulin dependent diabetes mellitus with ulcer |
| C109411 | Type II diabetes mellitus with ulcer |
| C109412 | Type 2 diabetes mellitus with ulcer |
| C109500 | Non-insulin dependent diabetes mellitus with gangrene |
| C109511 | Type II diabetes mellitus with gangrene |
| C109512 | Type 2 diabetes mellitus with gangrene |
| C109600 | Non-insulin-dependent diabetes mellitus with retinopathy |
| C109611 | Type II diabetes mellitus with retinopathy |
| C109612 | Type 2 diabetes mellitus with retinopathy |
| C109700 | Non-insulin dependent diabetes mellitus - poor control |
| C109711 | Type II diabetes mellitus - poor control |
| C109712 | Type 2 diabetes mellitus - poor control |
| C109900 | Non-insulin-dependent diabetes mellitus without complication |
| C109911 | Type II diabetes mellitus without complication |
| C109912 | Type 2 diabetes mellitus without complication |
| C109A00 | Non-insulin dependent diabetes mellitus with mononeuropathy |
| C109A11 | Type II diabetes mellitus with mononeuropathy |
| C109B00 | Non-insulin dependent diabetes mellitus with polyneuropathy |
| C109B11 | Type II diabetes mellitus with polyneuropathy |
| C109B12 | Type 2 diabetes mellitus with polyneuropathy |
| C109C00 | Non-insulin dependent diabetes mellitus with nephropathy |
| C109C11 | Type II diabetes mellitus with nephropathy |
| C109C12 | Type 2 diabetes mellitus with nephropathy |
| C109D00 | Non-insulin dependent diabetes mellitus with hypoglyca coma |
| C109D11 | Type II diabetes mellitus with hypoglycaemic coma |
| C109D12 | Type 2 diabetes mellitus with hypoglycaemic coma |
| C109E00 | Non-insulin depend diabetes mellitus with diabetic cataract |
| C109E11 | Type II diabetes mellitus with diabetic cataract |
| C109E12 | Type 2 diabetes mellitus with diabetic cataract |
| C109F00 | Non-insulin-dependent d m with peripheral angiopath |
| C109F11 | Type II diabetes mellitus with peripheral angiopathy |
| C109F12 | Type 2 diabetes mellitus with peripheral angiopathy |
| C109G00 | Non-insulin dependent diabetes mellitus with arthropathy |
| C109G11 | Type II diabetes mellitus with arthropathy |
| C109G12 | Type 2 diabetes mellitus with arthropathy |
| C109H00 | Non-insulin dependent d m with neuropathic arthropathy |
| C109H11 | Type II diabetes mellitus with neuropathic arthropathy |
| C109H12 | Type 2 diabetes mellitus with neuropathic arthropathy |
| C109J00 | Insulin treated Type 2 diabetes mellitus |
| C109J11 | Insulin treated non-insulin dependent diabetes mellitus |
| C109J12 | Insulin treated Type II diabetes mellitus |
| C109K00 | Hyperosmolar non-ketotic state in type 2 diabetes mellitus |
| C10C.11 | Maturity onset diabetes in youth |
| C10D.00 | Diabetes mellitus autosomal dominant type 2 |
| C10D.11 | Maturity onset diabetes in youth type 2 |
| C10F.00 | Type 2 diabetes mellitus |
| C10F.11 | Type II diabetes mellitus |
| C10F000 | Type 2 diabetes mellitus with renal complications |
| C10F011 | Type II diabetes mellitus with renal complications |
| C10F100 | Type 2 diabetes mellitus with ophthalmic complications |
| C10F111 | Type II diabetes mellitus with ophthalmic complications |
| C10F200 | Type 2 diabetes mellitus with neurological complications |
| C10F211 | Type II diabetes mellitus with neurological complications |
| C10F300 | Type 2 diabetes mellitus with multiple complications |
| C10F311 | Type II diabetes mellitus with multiple complications |
| C10F400 | Type 2 diabetes mellitus with ulcer |
| C10F411 | Type II diabetes mellitus with ulcer |
| C10F500 | Type 2 diabetes mellitus with gangrene |
| C10F511 | Type II diabetes mellitus with gangrene |
| C10F600 | Type 2 diabetes mellitus with retinopathy |
| C10F611 | Type II diabetes mellitus with retinopathy |
| C10F700 | Type 2 diabetes mellitus - poor control |
| C10F711 | Type II diabetes mellitus - poor control |
| C10F800 | Reaven's syndrome |
| C10F900 | Type 2 diabetes mellitus without complication |
| C10F911 | Type II diabetes mellitus without complication |
| C10FA00 | Type 2 diabetes mellitus with mononeuropathy |
| C10FA11 | Type II diabetes mellitus with mononeuropathy |
| C10FB00 | Type 2 diabetes mellitus with polyneuropathy |
| C10FB11 | Type II diabetes mellitus with polyneuropathy |
| C10FC00 | Type 2 diabetes mellitus with nephropathy |
| C10FC11 | Type II diabetes mellitus with nephropathy |
| C10FD00 | Type 2 diabetes mellitus with hypoglycaemic coma |
| C10FD11 | Type II diabetes mellitus with hypoglycaemic coma |
| C10FE00 | Type 2 diabetes mellitus with diabetic cataract |
| C10FE00 | Type 2 diabetes mellitus with diabetic cataract |
| C10FE11 | Type II diabetes mellitus with diabetic cataract |
| C10FF00 | Type 2 diabetes mellitus with peripheral angiopathy |
| C10FF11 | Type II diabetes mellitus with peripheral angiopathy |
| C10FG00 | Type 2 diabetes mellitus with arthropathy |
| C10FG11 | Type II diabetes mellitus with arthropathy |
| C10FH00 | Type 2 diabetes mellitus with neuropathic arthropathy |
| C10FH11 | Type II diabetes mellitus with neuropathic arthropathy |
| C10FJ00 | Insulin treated Type 2 diabetes mellitus |
| C10FJ11 | Insulin treated Type II diabetes mellitus |
| C10FK00 | Hyperosmolar non-ketotic state in type 2 diabetes mellitus |
| C10FK11 | Hyperosmolar non-ketotic state in type II diabetes mellitus |
| C10FL00 | Type 2 diabetes mellitus with persistent proteinuria |
| C10FL11 | Type II diabetes mellitus with persistent proteinuria |
| C10FM00 | Type 2 diabetes mellitus with persistent microalbuminuria |
| C10FM00 | Type 2 diabetes mellitus with persistent microalbuminuria |
| C10FM11 | Type II diabetes mellitus with persistent microalbuminuria |
| C10FN00 | Type 2 diabetes mellitus with ketoacidosis |
| C10FN11 | Type II diabetes mellitus with ketoacidosis |
| C10FP00 | Type 2 diabetes mellitus with ketoacidotic coma |
| C10FP11 | Type II diabetes mellitus with ketoacidotic coma |
| C10FQ00 | Type 2 diabetes mellitus with exudative maculopathy |
| C10FQ11 | Type II diabetes mellitus with exudative maculopathy |
| C10FR00 | Type 2 diabetes mellitus with gastroparesis |
| C10FR11 | Type II diabetes mellitus with gastroparesis |
| C10K000 | Type A insulin resistance without complication |
| C10N000 | Secondary diabetes mellitus without complication |
| C10P.00 | Diabetes mellitus in remission |
| C10P100 | Type II diabetes mellitus in remission |
| C10y100 | Diabetes mellitus, adult, + other specified manifestation |
| C10z.00 | Diabetes mellitus with unspecified complication |
| C10z100 | Diabetes mellitus, adult onset, + unspecified complication |
| **Non-specific Diabetes** |  |
| **Read codes** | **Description** |
| 1434 | H/O: diabetes mellitus |
| 3882 | Diabetes well being questionnaire |
| 3883 | Diabetes treatment satisfaction questionnaire |
| 7276 | Pan retinal photocoagulation for diabetes |
| 13AB.00 | Diabetic lipid lowering diet |
| 13AC.00 | Diabetic weight reducing diet |
| 13B1.00 | Diabetic diet |
| 14P3.00 | H/O: insulin therapy |
| 2BBF.00 | Retinal abnormality - diabetes related |
| 2BBJ.00 | O/E - no right diabetic retinopathy |
| 2BBK.00 | O/E - no left diabetic retinopathy |
| 2BBk.00 | O/E - right eye stable treated prolif diabetic retinopathy |
| 2BBL.00 | O/E - diabetic maculopathy present both eyes |
| 2BBl.00 | O/E - left eye stable treated prolif diabetic retinopathy |
| 2BBM.00 | O/E - diabetic maculopathy absent both eyes |
| 2BBo.00 | O/E - sight threatening diabetic retinopathy |
| 2BBR.00 | O/E - right eye preproliferative diabetic retinopathy |
| 2BBr.00 | Impaired vision due to diabetic retinopathy |
| 2BBS.00 | O/E - left eye preproliferative diabetic retinopathy |
| 2BBT.00 | O/E - right eye proliferative diabetic retinopathy |
| 2BBV.00 | O/E - left eye proliferative diabetic retinopathy |
| 2BBW.00 | O/E - right eye diabetic maculopathy |
| 2BBX.00 | O/E - left eye diabetic maculopathy |
| 2G5A.00 | O/E - Right diabetic foot at risk |
| 2G5B.00 | O/E - Left diabetic foot at risk |
| 2G5C.00 | Foot abnormality - diabetes related |
| 2G5d.00 | O/E - Left diabetic foot at increased risk |
| 2G5E.00 | O/E - Right diabetic foot at low risk |
| 2G5e.00 | O/E - Right diabetic foot at increased risk |
| 2G5F.00 | O/E - Right diabetic foot at moderate risk |
| 2G5G.00 | O/E - Right diabetic foot at high risk |
| 2G5H.00 | O/E - Right diabetic foot - ulcerated |
| 2G5I.00 | O/E - Left diabetic foot at low risk |
| 2G5J.00 | O/E - Left diabetic foot at moderate risk |
| 2G5K.00 | O/E - Left diabetic foot at high risk |
| 2G5L.00 | O/E - Left diabetic foot - ulcerated |
| 2G5V.00 | O/E - right chronic diabetic foot ulcer |
| 2G5W.00 | O/E - left chronic diabetic foot ulcer |
| 42c..00 | HbA1 - diabetic control |
| 42W..00 | Hb. A1C - diabetic control |
| 42WZ.00 | Hb. A1C - diabetic control NOS |
| 661M400 | Diabetes self-management plan agreed |
| 661N400 | Diabetes self-management plan review |
| 66A..00 | Diabetic monitoring |
| 66A1.00 | Initial diabetic assessment |
| 66A2.00 | Follow-up diabetic assessment |
| 66A5.00 | Diabetic on insulin |
| 66A8.00 | Has seen dietician - diabetes |
| 66A9.00 | Understands diet - diabetes |
| 66Aa.00 | Diabetic diet - poor compliance |
| 66Ab.00 | Diabetic foot examination |
| 66Ac.00 | Diabetic peripheral neuropathy screening |
| 66AD.00 | Fundoscopy - diabetic check |
| 66Af.00 | Patient diabetes education review |
| 66AG.00 | Diabetic drug side effects |
| 66AH.00 | Diabetic treatment changed |
| 66AH200 | Conversion to insulin by diabetes specialist nurse |
| 66Ai.00 | Diabetic 6 month review |
| 66AI.00 | Diabetic - good control |
| 66AJ.00 | Diabetic - poor control |
| 66AJ.11 | Unstable diabetes |
| 66AJz00 | Diabetic - poor control NOS |
| 66AK.00 | Diabetic - cooperative patient |
| 66Ak.00 | Diabetic monitoring - lower risk albumin excretion |
| 66AL.00 | Diabetic-uncooperative patient |
| 66Al.00 | Diabetic monitoring - higher risk albumin excretion |
| 66AM.00 | Diabetic - follow-up default |
| 66AN.00 | Date diabetic treatment start |
| 66AP.00 | Diabetes: practice programme |
| 66AQ.00 | Diabetes: shared care programme |
| 66Aq.00 | Diabetic foot screen |
| 66AR.00 | Diabetes management plan given |
| 66AS.00 | Diabetic annual review |
| 66As.00 | Diabetic on subcutaneous treatment |
| 66AS000 | Diabetes Year of Care annual review |
| 66At.00 | Diabetic dietary review |
| 66AT.00 | Annual diabetic blood test |
| 66AU.00 | Diabetes care by hospital only |
| 66Au.00 | Diabetic erectile dysfunction review |
| 66Av.00 | Diabetic assessment of erectile dysfunction |
| 66AW.00 | Diabetic foot risk assessment |
| 66AY.00 | Diabetic diet - good compliance |
| 66AZ.00 | Diabetic monitoring NOS |
| 66o..00 | Further diabetic monitoring |
| 679R.00 | Patient offered diabetes structured education programme |
| 67D8.00 | Provision of diabetes clinical summary |
| 68A7.00 | Diabetic retinopathy screening |
| 68A9.00 | Diabetic retinopathy screening offered |
| 68AB.00 | Diabetic digital retinopathy screening offered |
| 8A12.00 | Diabetic crisis monitoring |
| 8A13.00 | Diabetic stabilisation |
| 8B3l.00 | Diabetes medication review |
| 8BL2.00 | Patient on maximal tolerated therapy for diabetes |
| 8CA4100 | Pt advised re diabetic diet |
| 8CMW700 | Diabetes clinical pathway |
| 8CR2.00 | Diabetes clinical management plan |
| 8CS0.00 | Diabetes care plan agreed |
| 8H2J.00 | Admit diabetic emergency |
| 8H3O.00 | Non-urgent diabetic admission |
| 8H4e.00 | Referral to diabetes special interest general practitioner |
| 8H7C.00 | Refer, diabetic liaison nurse |
| 8H7f.00 | Referral to diabetes nurse |
| 8H7r.00 | Refer to diabetic foot screener |
| 8HBG.00 | Diabetic retinopathy 12 month review |
| 8HBH.00 | Diabetic retinopathy 6 month review |
| 8HHy.00 | Referral to diabetic register |
| 8Hj0.00 | Referral to diabetes structured education programme |
| 8Hl1.00 | Referral for diabetic retinopathy screening |
| 8Hl4.00 | Referral to community diabetes specialist nurse |
| 8Hlc.00 | Referral to community diabetes service |
| 8HTE100 | Referral to community diabetes clinic |
| 8HTi.00 | Referral to multidisciplinary diabetic clinic |
| 8HTk.00 | Referral to diabetic eye clinic |
| 8I3W.00 | Diabetic foot examination declined |
| 8I3X.00 | Diabetic retinopathy screening refused |
| 8I6G.00 | Diabetic foot examination not indicated |
| 8I94.00 | Diabetes structured education programme not available |
| 8IAs.00 | Diabetic dietary review declined |
| 8OA3.00 | Provision of written information about diabetes and driving |
| 9m0..00 | Diabetic retinopathy screening administrative status |
| 9m00.00 | Eligible for diabetic retinopathy screening |
| 9m0A.00 | Declined diabetic retinopathy screening |
| 9N0m.00 | Seen in diabetic nurse consultant clinic |
| 9N0n.00 | Seen in community diabetes specialist clinic |
| 9N0o.00 | Seen in community diabetic specialist nurse clinic |
| 9N1i.00 | Seen in diabetic foot clinic |
| 9N1o.00 | Seen in multidisciplinary diabetic clinic |
| 9N1Q.00 | Seen in diabetic clinic |
| 9N2i.00 | Seen by diabetic liaison nurse |
| 9N4I.00 | DNA - Did not attend diabetic clinic |
| 9N4p.00 | Did not attend diabetic retinopathy clinic |
| 9NiZ.00 | Did not attend diabetes foot screening |
| 9NN9.00 | Under care of diabetes specialist nurse |
| 9NND.00 | Under care of diabetic foot screener |
| 9OL..00 | Diabetes monitoring admin. |
| 9OL..11 | Diabetes clinic administration |
| 9OL1.00 | Attends diabetes monitoring |
| 9OL2.00 | Refuses diabetes monitoring |
| 9OL3.00 | Diabetes monitoring default |
| 9OL4.00 | Diabetes monitoring 1st letter |
| 9OL5.00 | Diabetes monitoring 2nd letter |
| 9OL6.00 | Diabetes monitoring 3rd letter |
| 9OL7.00 | Diabetes monitor.verbal invite |
| 9OL8.00 | Diabetes monitor.phone invite |
| 9OLA.00 | Diabetes monitor. check done |
| 9OLA.11 | Diabetes monitored |
| 9OLB.00 | Attended diabetes structured education programme |
| 9OLD.00 | Diabetic patient unsuitable for digital retinal photography |
| 9OLF.00 | Diabetes structured education programme completed |
| 9OLM.00 | Diabetes structured education programme declined |
| 9OLN.00 | Diabetes monitor invitation by SMS (short message service) |
| 9OLZ.00 | Diabetes monitoring admin.NOS |
| 9Oy..00 | Diabetes screening administration |
| 9Oy0000 | Diabetic foot screening invitation |
| 9Oy0200 | Diabetic foot screening invitation first letter |
| 9Oy0300 | Diabetic foot screening invitation second letter |
| 9Oy0400 | Diabetic foot screening invitation third letter |
| C10..00 | Diabetes mellitus |
| C100.00 | Diabetes mellitus with no mention of complication |
| C100111 | Maturity onset diabetes |
| C100z00 | Diabetes mellitus NOS with no mention of complication |
| C101.00 | Diabetes mellitus with ketoacidosis |
| C101y00 | Other specified diabetes mellitus with ketoacidosis |
| C101z00 | Diabetes mellitus NOS with ketoacidosis |
| C103.00 | Diabetes mellitus with ketoacidotic coma |
| C103100 | Diabetes mellitus, adult onset, with ketoacidotic coma |
| C103z00 | Diabetes mellitus NOS with ketoacidotic coma |
| C104.00 | Diabetes mellitus with renal manifestation |
| C104.11 | Diabetic nephropathy |
| C104z00 | Diabetes mellitus with nephropathy NOS |
| C106.00 | Diabetes mellitus with neurological manifestation |
| C106.12 | Diabetes mellitus with neuropathy |
| C106.13 | Diabetes mellitus with polyneuropathy |
| C106z00 | Diabetes mellitus NOS with neurological manifestation |
| C107.00 | Diabetes mellitus with peripheral circulatory disorder |
| C107.11 | Diabetes mellitus with gangrene |
| C107.12 | Diabetes with gangrene |
| C107z00 | Diabetes mellitus NOS with peripheral circulatory disorder |
| C10FK11 | Hyperosmolar non-ketotic state in type II diabetes mellitus |
| C10z.00 | Diabetes mellitus with unspecified complication |
| Cyu2.00 | [X]Diabetes mellitus |
| F171100 | Autonomic neuropathy due to diabetes |
| F345000 | Diabetic mononeuritis multiplex |
| F35z000 | Diabetic mononeuritis NOS |
| F372.00 | Polyneuropathy in diabetes |
| F372.11 | Diabetic polyneuropathy |
| F372.12 | Diabetic neuropathy |
| F372000 | Acute painful diabetic neuropathy |
| F372100 | Chronic painful diabetic neuropathy |
| F372200 | Asymptomatic diabetic neuropathy |
| F381311 | Diabetic amyotrophy |
| F3y0.00 | Diabetic mononeuropathy |
| F420100 | Proliferative diabetic retinopathy |
| F420200 | Preproliferative diabetic retinopathy |
| F420300 | Advanced diabetic maculopathy |
| F420400 | Diabetic maculopathy |
| F420600 | Non proliferative diabetic retinopathy |
| F420700 | High risk proliferative diabetic retinopathy |
| F420800 | High risk non proliferative diabetic retinopathy |
| F420z00 | Diabetic retinopathy NOS |
| F440700 | Diabetic iritis |
| F464000 | Diabetic cataract |
| G73y000 | Diabetic peripheral angiopathy |
| K01x100 | Nephrotic syndrome in diabetes mellitus |
| K08yA00 | Proteinuric diabetic nephropathy |
| K08yA11 | Clinical diabetic nephropathy |
| K27y700 | Erectile dysfunction due to diabetes mellitus |
| M037200 | Cellulitis in diabetic foot |
| M271000 | Ischaemic ulcer diabetic foot |
| M271100 | Neuropathic diabetic ulcer - foot |
| M271200 | Mixed diabetic ulcer - foot |
| N030000 | Diabetic cheiroarthropathy |
| N030100 | Diabetic Charcot arthropathy |
| R054200 | [D]Gangrene of toe in diabetic |
| R054300 | [D]Widespread diabetic foot gangrene |
| ZC2C800 | Dietary advice for diabetes mellitus |
| ZL22500 | Under care of diabetic liaison nurse |
| ZL62500 | Referral to diabetes nurse |
| ZL62600 | Referral to diabetic liaison nurse |
| ZLA2500 | Seen by diabetic liaison nurse |
| ZRB4.00 | Diabetes clinic satisfaction questionnaire |
| ZRB6.00 | Diabetes wellbeing questionnaire |
| ZV65312 | [V]Dietary counselling in diabetes mellitus |
| **Type 1 Diabetes** |  |
| **Read Codes** | **Description** |
| 66AJ100 | Brittle diabetes |
| 66An.00 | Diabetes type 1 review |
| 66At011 | Type 1 diabetic dietary review |
| C100000 | Diabetes mellitus, juvenile type, no mention of complication |
| C100011 | Insulin dependent diabetes mellitus |
| C101000 | Diabetes mellitus, juvenile type, with ketoacidosis |
| C102000 | Diabetes mellitus, juvenile type, with hyperosmolar coma |
| C103000 | Diabetes mellitus, juvenile type, with ketoacidotic coma |
| C104000 | Diabetes mellitus, juvenile type, with renal manifestation |
| C105000 | Diabetes mellitus, juvenile type, + ophthalmic manifestation |
| C106000 | Diabetes mellitus, juvenile, + neurological manifestation |
| C107000 | Diabetes mellitus, juvenile +peripheral circulatory disorder |
| C107300 | IDDM with peripheral circulatory disorder |
| C108.00 | Insulin dependent diabetes mellitus |
| C108.11 | IDDM-Insulin dependent diabetes mellitus |
| C108.12 | Type 1 diabetes mellitus |
| C108.13 | Type I diabetes mellitus |
| C108000 | Insulin-dependent diabetes mellitus with renal complications |
| C108011 | Type I diabetes mellitus with renal complications |
| C108012 | Type 1 diabetes mellitus with renal complications |
| C108100 | Insulin-dependent diabetes mellitus with ophthalmic comps |
| C108112 | Type 1 diabetes mellitus with ophthalmic complications |
| C108200 | Insulin-dependent diabetes mellitus with neurological comps |
| C108211 | Type I diabetes mellitus with neurological complications |
| C108212 | Type 1 diabetes mellitus with neurological complications |
| C108300 | Insulin dependent diabetes mellitus with multiple complicatn |
| C108311 | Type I diabetes mellitus with multiple complications |
| C108400 | Unstable insulin dependent diabetes mellitus |
| C108411 | Unstable type I diabetes mellitus |
| C108412 | Unstable type 1 diabetes mellitus |
| C108500 | Insulin dependent diabetes mellitus with ulcer |
| C108511 | Type I diabetes mellitus with ulcer |
| C108512 | Type 1 diabetes mellitus with ulcer |
| C108600 | Insulin dependent diabetes mellitus with gangrene |
| C108700 | Insulin dependent diabetes mellitus with retinopathy |
| C108712 | Type 1 diabetes mellitus with retinopathy |
| C108800 | Insulin dependent diabetes mellitus - poor control |
| C108812 | Type 1 diabetes mellitus - poor control |
| C108900 | Insulin dependent diabetes maturity onset |
| C108911 | Type I diabetes mellitus maturity onset |
| C108912 | Type 1 diabetes mellitus maturity onset |
| C108A00 | Insulin-dependent diabetes without complication |
| C108A11 | Type I diabetes mellitus without complication |
| C108A12 | Type 1 diabetes mellitus without complication |
| C108B00 | Insulin dependent diabetes mellitus with mononeuropathy |
| C108B11 | Type I diabetes mellitus with mononeuropathy |
| C108C00 | Insulin dependent diabetes mellitus with polyneuropathy |
| C108D00 | Insulin dependent diabetes mellitus with nephropathy |
| C108D11 | Type I diabetes mellitus with nephropathy |
| C108D12 | Type 1 diabetes mellitus with nephropathy |
| C108E00 | Insulin dependent diabetes mellitus with hypoglycaemic coma |
| C108E11 | Type I diabetes mellitus with hypoglycaemic coma |
| C108E12 | Type 1 diabetes mellitus with hypoglycaemic coma |
| C108F00 | Insulin dependent diabetes mellitus with diabetic cataract |
| C108F11 | Type I diabetes mellitus with diabetic cataract |
| C108G00 | Insulin dependent diab mell with peripheral angiopathy |
| C108H00 | Insulin dependent diabetes mellitus with arthropathy |
| C108H11 | Type I diabetes mellitus with arthropathy |
| C108J00 | Insulin dependent diab mell with neuropathic arthropathy |
| C108J11 | Type I diabetes mellitus with neuropathic arthropathy |
| C108J12 | Type 1 diabetes mellitus with neuropathic arthropathy |
| C10E.00 | Type 1 diabetes mellitus |
| C10E.11 | Type I diabetes mellitus |
| C10E.12 | Insulin dependent diabetes mellitus |
| C10E000 | Type 1 diabetes mellitus with renal complications |
| C10E011 | Type I diabetes mellitus with renal complications |
| C10E012 | Insulin-dependent diabetes mellitus with renal complications |
| C10E100 | Type 1 diabetes mellitus with ophthalmic complications |
| C10E111 | Type I diabetes mellitus with ophthalmic complications |
| C10E112 | Insulin-dependent diabetes mellitus with ophthalmic comps |
| C10E200 | Type 1 diabetes mellitus with neurological complications |
| C10E212 | Insulin-dependent diabetes mellitus with neurological comps |
| C10E300 | Type 1 diabetes mellitus with multiple complications |
| C10E311 | Type I diabetes mellitus with multiple complications |
| C10E312 | Insulin dependent diabetes mellitus with multiple complicat |
| C10E400 | Unstable type 1 diabetes mellitus |
| C10E411 | Unstable type I diabetes mellitus |
| C10E412 | Unstable insulin dependent diabetes mellitus |
| C10E500 | Type 1 diabetes mellitus with ulcer |
| C10E511 | Type I diabetes mellitus with ulcer |
| C10E512 | Insulin dependent diabetes mellitus with ulcer |
| C10E600 | Type 1 diabetes mellitus with gangrene |
| C10E611 | Type I diabetes mellitus with gangrene |
| C10E612 | Insulin dependent diabetes mellitus with gangrene |
| C10E700 | Type 1 diabetes mellitus with retinopathy |
| C10E712 | Insulin dependent diabetes mellitus with retinopathy |
| C10E800 | Type 1 diabetes mellitus - poor control |
| C10E812 | Insulin dependent diabetes mellitus - poor control |
| C10E900 | Type 1 diabetes mellitus maturity onset |
| C10E911 | Type I diabetes mellitus maturity onset |
| C10E912 | Insulin dependent diabetes maturity onset |
| C10EA00 | Type 1 diabetes mellitus without complication |
| C10EA11 | Type I diabetes mellitus without complication |
| C10EA12 | Insulin-dependent diabetes without complication |
| C10EB00 | Type 1 diabetes mellitus with mononeuropathy |
| C10EC00 | Type 1 diabetes mellitus with polyneuropathy |
| C10EC11 | Type I diabetes mellitus with polyneuropathy |
| C10EC12 | Insulin dependent diabetes mellitus with polyneuropathy |
| C10ED00 | Type 1 diabetes mellitus with nephropathy |
| C10ED12 | Insulin dependent diabetes mellitus with nephropathy |
| C10EE00 | Type 1 diabetes mellitus with hypoglycaemic coma |
| C10EE12 | Insulin dependent diabetes mellitus with hypoglycaemic coma |
| C10EF00 | Type 1 diabetes mellitus with diabetic cataract |
| C10EF12 | Insulin dependent diabetes mellitus with diabetic cataract |
| C10EG00 | Type 1 diabetes mellitus with peripheral angiopathy |
| C10EH00 | Type 1 diabetes mellitus with arthropathy |
| C10EJ00 | Type 1 diabetes mellitus with neuropathic arthropathy |
| C10EK00 | Type 1 diabetes mellitus with persistent proteinuria |
| C10EL00 | Type 1 diabetes mellitus with persistent microalbuminuria |
| C10EL11 | Type I diabetes mellitus with persistent microalbuminuria |
| C10EM00 | Type 1 diabetes mellitus with ketoacidosis |
| C10EM11 | Type I diabetes mellitus with ketoacidosis |
| C10EN00 | Type 1 diabetes mellitus with ketoacidotic coma |
| C10EN11 | Type I diabetes mellitus with ketoacidotic coma |
| C10EP00 | Type 1 diabetes mellitus with exudative maculopathy |
| C10EP11 | Type I diabetes mellitus with exudative maculopathy |
| C10EQ00 | Type 1 diabetes mellitus with gastroparesis |
| C10EQ11 | Type I diabetes mellitus with gastroparesis |
| C10P011 | Type 1 diabetes mellitus in remission |
| C10z000 | Diabetes mellitus, juvenile type, + unspecified complication |

**Supplementary Table 2.**

**International Classification of Diseases 10th edition code list for the ascertainment of Type 1 diabetes and Type 2 diabetes in the Clinical Practice Research Datalink**

| **Type 1 Diabetes** |  |
| --- | --- |
| **ICD code** | **Description** |
| E10 | Type 1 diabetes mellitus |
| E10.0 | With coma |
| E10.1 | With ketoacidosis |
| E10.2† | With renal complications |
| N08.3* | Diabetic nephropathy |
| E10.3† | With ophthalmic complications |
| H36.0* | Diabetic: retinopathy |
| E10.4† | With neurological complications |
| G73.0* | Diabetic: amyotrophy |
| G99.0* | Diabetic: autonomic neuropathy |
| G59.0* | Diabetic: mononeuropathy |
| G63.2* | Diabetic: polyneuropathy |
| E10.5 | With peripheral circulatory complications |
| E10.6 | With other specified complications |
| E10.7 | With multiple complications |
| E10.8 | With unspecified complications |
| E10.9 | Without complications |
| **Type 2 Diabetes** |  |
| **ICD code** | **Description** |
| E11 | Type 2 diabetes mellitus |
| E11.0 | With coma |
| E11.1 | With ketoacidosis |
| E11.2† | With renal complications |
| N08.3* | Diabetic nephropathy |
| E11.3† | With ophthalmic complications |
| H36.0* | Diabetic: retinopathy |
| E11.4† | With neurological complications |
| G73.0* | Diabetic: amyotrophy |
| G99.0* | Diabetic: autonomic neuropathy |
| G59.0* | Diabetic: mononeuropathy |
| G63.2* | Diabetic: polyneuropathy |
| E11.5 | With peripheral circulatory complications |
| E11.6 | With other specified complications |
| E11.7 | With multiple complications |
| E11.8 | With unspecified complications |
| E11.9 | Without complications |

**Supplementary Table 3.**

**Prescription code list for the ascertainment of Type 1 diabetes and Type 2 diabetes in the Clinical Practice Research Datalink.**

| **Type 2 Diabetes** |  |
| --- | --- |
| **BNF code** | **BNF Header** |
| 6010151 | Biphasic Insulins |
| 6010102 | Intermediate- and Long-Acting Insulins |
| 6010101 | Short-acting Insulins |
| 6010100 | Insulins |
| 6010202 | Biguanids |
| 6010203 | Other antidiabetic drugs |
| 6010201 | Sulphonylureas |


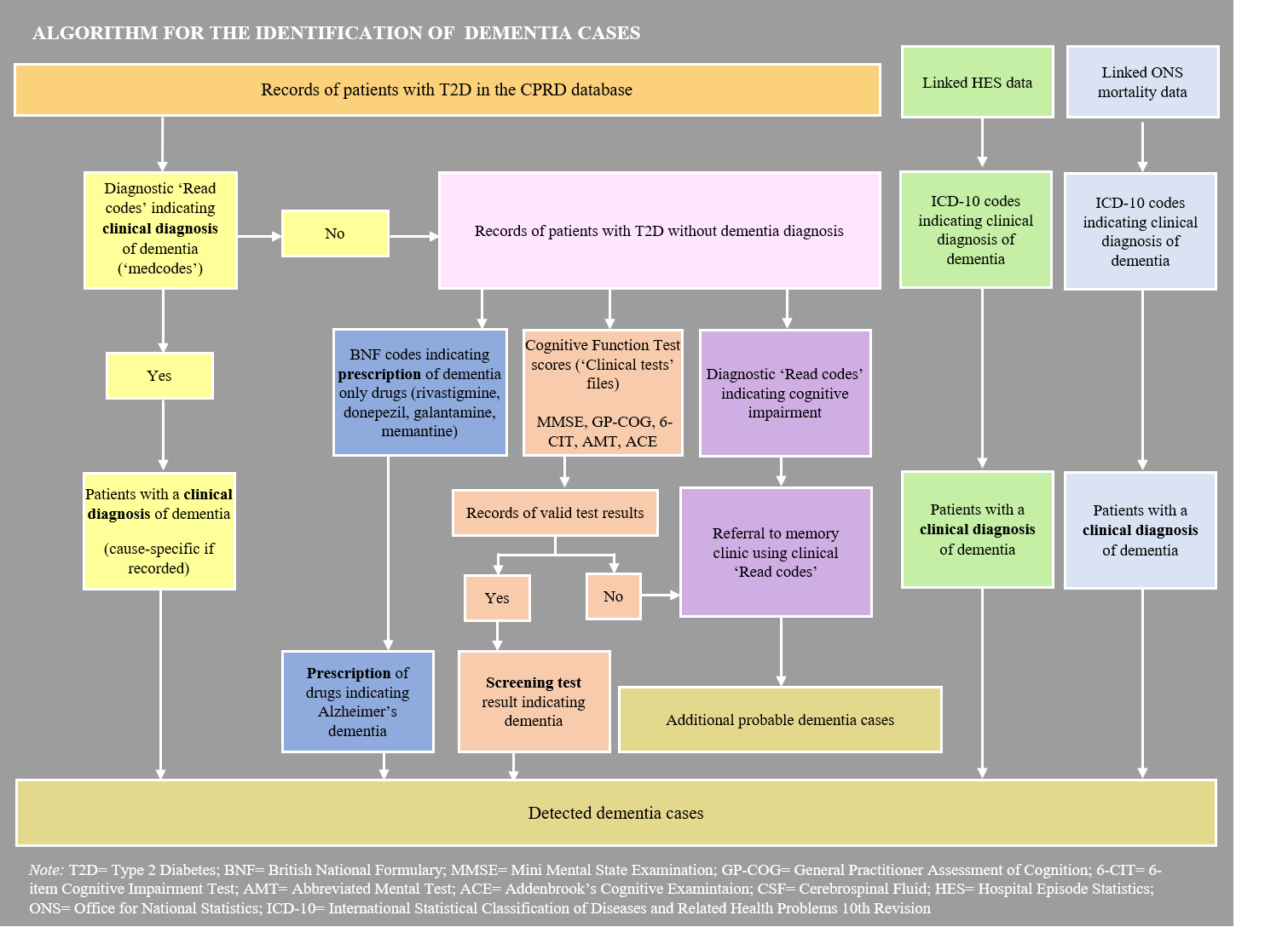


**Supplementary Figure 1. Algorithm for the identification of dementia cases within the Clinical Practice Research Datalink, Hospital Episode Statistics, and Office of National Statistics**

**Supplementary Table 4.**

**Code list for the ascertainment of subtypes of dementia in the Clinical Practice Research Datalink.(3)**

| **Vascular Dementia** |  |
| --- | --- |
| **Read Code** | **Read Term** |
| E004.00 | Arteriosclerotic dementia |
| E004.11 | Multi infarct dementia |
| E004000 | Uncomplicated arteriosclerotic dementia |
| E004100 | Arteriosclerotic dementia with delirium |
| E004200 | Arteriosclerotic dementia with paranoia |
| E004300 | Arteriosclerotic dementia with depression |
| E004z00 | Arteriosclerotic dementia NOS |
| Eu01.00 | [X]Vascular dementia |
| Eu01.11 | [X]Arteriosclerotic dementia |
| Eu01000 | [X]Vascular dementia of acute onset |
| Eu01100 | [X]Multi-infarct dementia |
| Eu01200 | [X]Subcortical vascular dementia |
| Eu01300 | [X]Mixed cortical and subcortical vascular dementia |
| Eu01y00 | [X]Other vascular dementia |
| Eu01z00 | [X]Vascular dementia, unspecified |
| F11x200 | Cerebral degeneration due to cerebrovascular disease |
| F11x200 | Cerebral degeneration due to cerebrovascular disease |
| **Alzheimer’s Dementia** |  |
| **Read Code** | **Read Term** |
| E00..11 | Senile dementia |
| E00..12 | Senile/presenile dementia |
| E000.00 | Uncomplicated senile dementia |
| E001000 | Uncomplicated presenile dementia |
| E001300 | Presenile dementia with depression |
| E002100 | Senile dementia with depression |
| Eu00.00 | [X]Dementia in Alzheimer's disease |
| Eu00000 | [X]Dementia in Alzheimer's disease with early onset |
| Eu00011 | [X]Presenile dementia,Alzheimer's type |
| Eu00011 | [X]Presenile dementia,Alzheimer's type |
| Eu00012 | [X]Primary degen dementia, Alzheimer's type, presenile onset |
| Eu00013 | [X]Alzheimer's disease type 2 |
| Eu00100 | [X]Dementia in Alzheimer's disease with late onset |
| Eu00111 | [X]Alzheimer's disease type 1 |
| Eu00112 | [X]Senile dementia,Alzheimer's type |
| Eu00113 | [X]Primary degen dementia of Alzheimer's type, senile onset |
| Eu00200 | [X]Dementia in Alzheimer's dis, atypical or mixed type |
| Eu00z00 | [X]Dementia in Alzheimer's disease, unspecified |
| Eu00z11 | [X]Alzheimer's dementia unspec |
| Eu01111 | [X]Predominantly cortical dementia |
| Eu02z13 | [X] Primary degenerative dementia NOS |
| Eu02z14 | [X] Senile dementia NOS |
| F110.00 | Alzheimer's disease |
| F110000 | Alzheimer's disease with early onset |
| F110100 | Alzheimer's disease with late onset |
| F110100 | Alzheimer's disease with late onset |
| F112.00 | Senile degeneration of brain |
| F11z.11 | Cerebral atrophy |
| Fyu3000 | [X]Other Alzheimer's disease |
| **Other/Unspecific Dementia** |  |
| **Read Code** | **Read Term** |
| 6AB..00 | Dementia annual review |
| 1461.00 | H/O: dementia |
| 1JA2.00 | Suspected dementia |
| 28E2.00 | Severe cognitive impairment |
| 38C1300 | Assessment of psychotic and behavioural symptoms of dementia |
| 3AE4.00 | GDS level 5 - moderately severe cognitive decline |
| 3AE5.00 | GDS level 6 - severe cognitive decline |
| 3AE6.00 | GDS level 7 - very severe cognitive decline |
| 66h..00 | Dementia monitoring |
| 6AB..00 | Dementia annual review |
| 8BM0200 | Dementia medication review |
| 8BPa.00 | Antipsychotic drug therapy for dementia |
| 8CMe000 | Dementia advance care plan |
| 8CMG200 | Review of dementia advance care plan |
| 8CMZ.00 | Dementia care plan |
| 8CMZ.00 | Dementia care plan |
| 8CMZ000 | Dementia care plan agreed |
| 8CMZ100 | Dementia care plan reviewed |
| 8CMZ200 | Dementia care plan declined |
| 8CMZ300 | Dementia care plan review declined |
| 8CSA.00 | Dementia advance care plan agreed |
| 8Hla.00 | Referral to dementia care advisor |
| 8Hla.00 | Referral to dementia care advisor |
| 8IAe000 | Dementia advance care plan declined |
| 8IAe200 | Dementia advance care plan review declined |
| 8T05000 | Referral to dementia support organisation |
| 8T05100 | Referral to dementia support organisation declined |
| 9Ou..00 | Dementia monitoring administration |
| 9Ou1.00 | Dementia monitoring first letter |
| 9Ou2.00 | Dementia monitoring second letter |
| 9Ou3.00 | Dementia monitoring third letter |
| 9Ou4.00 | Dementia monitoring verbal invite |
| 9Ou5.00 | Dementia monitoring telephone invite |
| 9Ou5.00 | Dementia monitoring telephone invite |
| A411.00 | Jakob-Creutzfeldt disease |
| E00..00 | Senile and presenile organic psychotic conditions |
| E001.00 | Presenile dementia |
| E001000 | Uncomplicated presenile dementia |
| E001100 | Presenile dementia with delirium |
| E001200 | Presenile dementia with paranoia |
| E001300 | Presenile dementia with depression |
| E001z00 | Presenile dementia NOS |
| E002.00 | Senile dementia with depressive or paranoid features |
| E002000 | Senile dementia with paranoia |
| E002z00 | Senile dementia with depressive or paranoid features NOS |
| E003.00 | Senile dementia with delirium |
| E00y.00 | Other senile and presenile organic psychoses |
| E012.00 | Other alcoholic dementia |
| E012.11 | Alcoholic dementia NOS |
| E02y100 | Drug-induced dementia |
| E041.00 | Dementia in conditions EC |
| Eu02.00 | [X]Dementia in other diseases classified elsewhere |
| Eu02000 | [X]Dementia in Pick's disease |
| Eu02100 | [X]Dementia in Creutzfeldt-Jakob disease |
| Eu02200 | [X]Dementia in Huntington's disease |
| Eu02300 | [X]Dementia in Parkinson's disease |
| Eu02400 | [X]Dementia in human immunodef virus [HIV] disease |
| Eu02500 | [X]Lewy body dementia |
| Eu02y00 | [X]Dementia in other specified diseases classif elsewhere |
| Eu02z00 | [X] Unspecified dementia |
| Eu02z11 | [X] Presenile dementia NOS |
| Eu02z16 | [X] Senile dementia, depressed or paranoid type |
| Eu04100 | [X]Delirium superimposed on dementia |
| Eu10711 | [X]Alcoholic dementia NOS |
| F103.00 | Cerebral degeneration in diseases EC |
| F103000 | Cerebral degeneration in Hunter's disease |
| F11..00 | Other cerebral degenerations |
| F111.00 | Pick's disease |
| F116.00 | Lewy body disease |
| F118.00 | Frontotemporal degeneration |
| F11x000 | Cerebral degeneration due to alcoholism |
| F11x000 | Cerebral degeneration due to alcoholism |
| F11x600 | Cerebral degeneration due to vitamin B12 deficiency |
| F11x700 | Cerebral degeneration due to Jakob - Creutzfeldt disease |
| F11x900 | Cerebral degeneration in Parkinson's disease |
| F11z.00 | Cerebral degeneration NOS |
| F134.00 | Huntington's chorea |
| ZS7C500 | Language disorder of dementia |
| **Memory loss or impairment, or cognitive impairment** |  |
| **Read Code** | **Read Term** |
| 1B1A.00 | Memory loss - amnesia |
| 1B1A.12 | Memory loss symptom |
| 1B1A.13 | Memory disturbance |
| 1B1A.13 | Memory disturbance |
| 1B1A100 | Short-term memory loss |
| 1S21.00 | Disturbance of memory for order of events |
| 1S23.00 | Memory impairment |
| 28E..0 | Cognitive decline |
| 28E0.00 | Mild cognitive impairment |
| 28E1.00 | Moderate cognitive impairment |
| 28E3.00 | Cognitive impairment |
| 3A10.00 | Memory: own age not known |
| 3A20.00 | Memory: present time not known |
| 3A30.00 | Memory: present place not knwn |
| 3A40.00 | Memory: present year not known |
| 3A50.00 | Memory: own DOB not known |
| 3A60.00 | Memory: present month not knwn |
| 3A70.00 | Memory: important event not kn |
| 3A80.00 | Memory: import.person not knwn |
| 3A91.00 | Memory: count down unsuccess. |
| 3AE2.00 | GDS level 3 - mild cognitive decline |
| 3AE3.00 | GDS level 4 - moderate cognitive decline |
| E2A1000 | Mild memory disturbance |
| E2A1100 | Organic memory impairment |
| Eu05700 | [X]Mild cognitive disorder |
| R00z011 | [D]Memory deficit |
| Z7CE412 | Memory loss symptom |
| Z7CE413 | Memory loss - amnesia |
| Z7CE414 | Memory disturbance |
| Z7CE415 | Loss of memory |
| Z7CE611 | Memory loss |
| Z7CE612 | Memory gone |
| Z7CE614 | Memory loss - amnesia |
| Z7CE615 | Loss of memory |
| Z7CE616 | LOM - Loss of memory |
| Z7CEA11 | Impairment of working memory |
| Z7CEA13 | Impairment of primary memory |
| Z7CEB12 | Poor memory for remote events |
| Z7CEC11 | Loss of memory for recent events |
| Z7CEC12 | No memory for recent events |
| Z7CEH00 | Memory impairment |
| Z7CEH11 | Memory dysfunction |
| Z7CEH12 | Memory deficit |
| Z7CEH13 | Bad memory |
| Z7CEH14 | Memory problem |
| Z7CEH15 | Poor memory |
| Z7CEJ00 | Memory lapses |
| Z7CEK00 | Minor memory lapses |
| Z7CEL00 | Mild memory disturbance |
| Z7CEM00 | Distortion of memory |
| Z7CF800 | Poor short-term memory |
| Z7CF811 | Short-term memory loss |
| Z7CFO00 | Poor long-term memory |
| Z7CFO11 | Long-term memory loss |
| Z7CFw00 | Memory aided by use of diary |
| Z7CFx00 | Memory aided by use of labels |
| Z7CFz00 | Memory aided by use of lists |
| Z7CGP00 | Delayed verbal memory |
| **Referral to memory clinic** |  |
| **Read Code** | **Read Term** |
| 8HTY.00 | Referral to memory clinic |
| 8IEn.00 | Referral to memory clinic declined |
| 8T05.00 | Referral to dementia service |
| 8T05200 | Referral for dementia assessment |
| 9Nk1.00 | Seen in memory clinic |
| **Cognitive Assessment** |  |
| **Read Code** | **Read Term** |
| 311B.00 | Cognitive assessment |
| 388L.00 | Abbreviated mental test |
| 388m.00 | Mini-mental state examination |
| 388m.11 | MMSE score |
| 388V.00 | Mini mental state score |
| 38Dv.00 | GPCOG - general practitioner assessment of cognition |
| 38Dv000 | GPCOG (GP assessment of cognition) patient examination |
| 38Dv100 | GPCOG (GP assessment of cognition) informant interview |
| 38QN.00 | Generalised anxiety disorder 2 scale |
| 3AD3.00 | Six item cognitive impairment test |
| 3AF..00 | Addenbrooke's cognitive examination revised |
| ZR16.00 | Abbreviated mental test |
| ZR16.11 | AMT - Abbreviated mental test |
| ZRa2.00 | Microcog - assessment of cognitive function |
| ZRaA.00 | Mini-mental state examination |

**Supplementary Table 5.**

**International Classification of Diseases 10^th^ edition code list for the ascertainment of subtypes of dementia in the Clinical Practice Research Datalink.**

| **Vascular Dementia** |  |
| --- | --- |
| **ICD code** | **Description** |
| F01+ | Vascular dementia incl.: Arteriosceloritic dementia |
| **Alzheimer’s Dementia** |  |
| **ICD code** | **Description** |
| F00* | Dementia in Alzheimer’s disease |
| F00.0* | Dementia in Alzheimer’s disease with early onset |
| F00.1* | Dementia in Alzheimer’s disease with late onset |
| F00.2* | Dementia in Alzheimer’s disease, atypical or mixed |
| F00.9* | Dementia in Alzheimer’s disease, unspecified |
| G30.0+ | Dementia in Alzheimer’s disease with early onset |
| G30.1+ | Dementia in Alzheimer’s disease with late onset |
| G30.8+ | Dementia in Alzheimer’s disease, atypical or mixed |
| G30.9+ | Dementia in Alzheimer’s disease, unspecified |
| **Other/Unspecific Dementia** |  |
| **ICD code** | **Description** |
| F02* | Dementia in other diseases classified elsewhere |
| F02.0* | Dementia in Pick disease |
| F02.1* | Dementia in Creutzfeldt-Jakob disease |
| F02.2* | Dementia in Huntington disease |
| F02.3* | Dementia in Parkinson disease |
| F02.4* | Dementia in human immunodeficiency virus [HIV] disease |
| F02.8* | Dementia in other specified diseases classified elsewhere |
| F03 | Unspecified dementia |
| F05.1 | Delirium superimposed on dementia. Senile dementia with delirium |
| F10.7 | Dementia- alcoholic |
| G31.0 | Circumscribed brain atrophy including froto-temporal dementia, Pick's) |
| G31.1 | Senile degenerati on of the brain, not elsewhere classified |
| G31.8 | Other specified degenerative disease of the nervous system. Including Grey matter degeneration; Lewy body disease; Lewy body dementia; Subacute necrotizing encephalopathy |

**Supplementary Table 6.**

**Prescription code list for the ascertainment of Alzheimer’s dementia in the Clinical Practice Research Datalink.**

| **Alzheimer’s Dementia** |  |
| --- | --- |
| **BNF code** | **BNF Header** |
| 4110000 | Drugs for Dementia: Rivastigmine |
| 4110000 | Drugs for Dementia: Galantamine |
| 4110000 | Drugs for Dementia: Memantine |
| 4110000 | Drugs for Dementia: Donepezil |


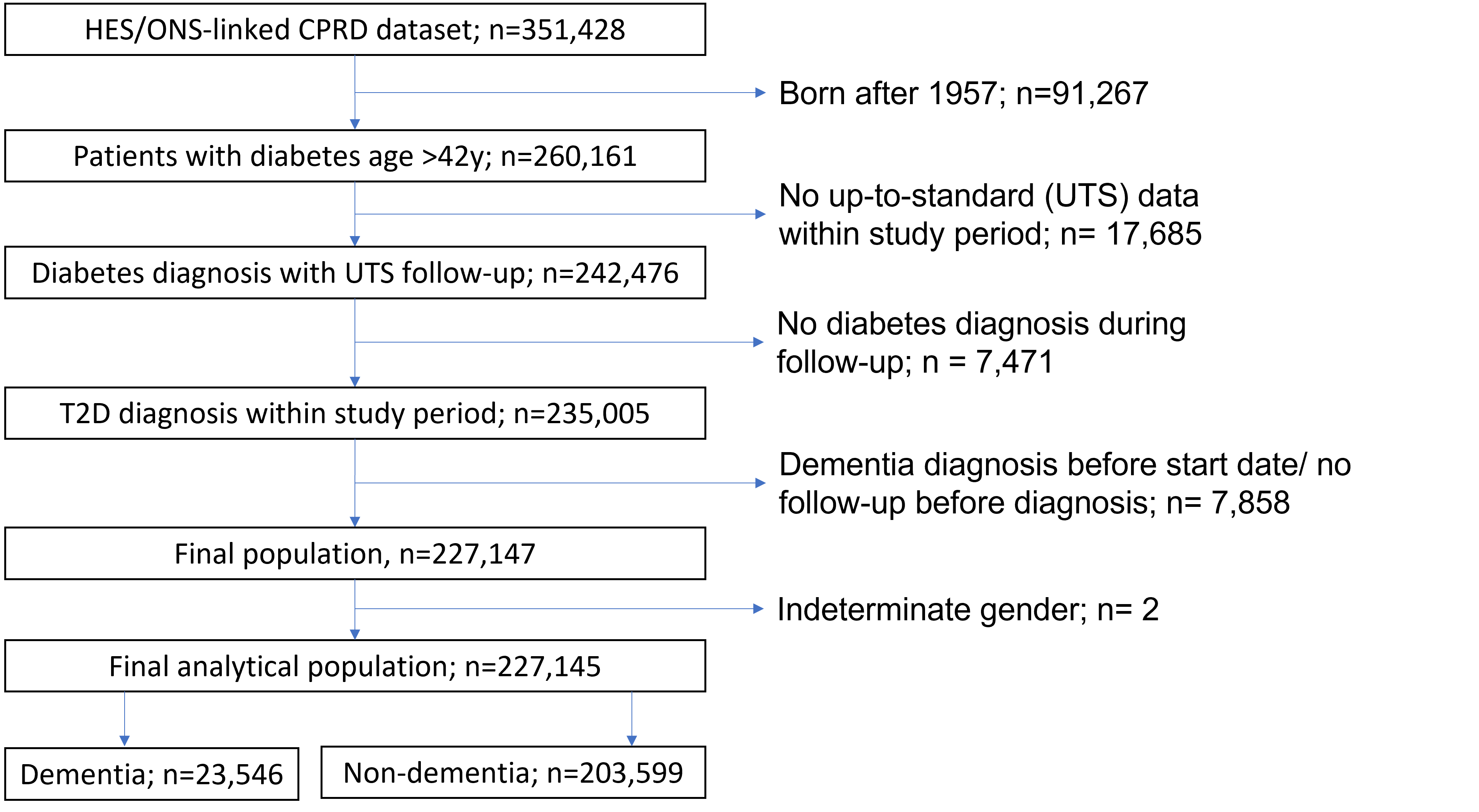


**Supplementary Figure 2. Study flowchart for obtaining the analytical population in the Clinical Practice Research Datalink with linkage to Hospital Episode Statistics (HES) and Office of National Statistics (ONS) mortality data in England.**

Up-to-standard dates are determined by CPRD for general practices, depending on the continuity of patient and death records, which define a period where practices meet the criteria; patient acceptability is a quality metric based on valid age and gender, registration status, and recording of events.(2) All patients in the linked dataset had acceptable data.


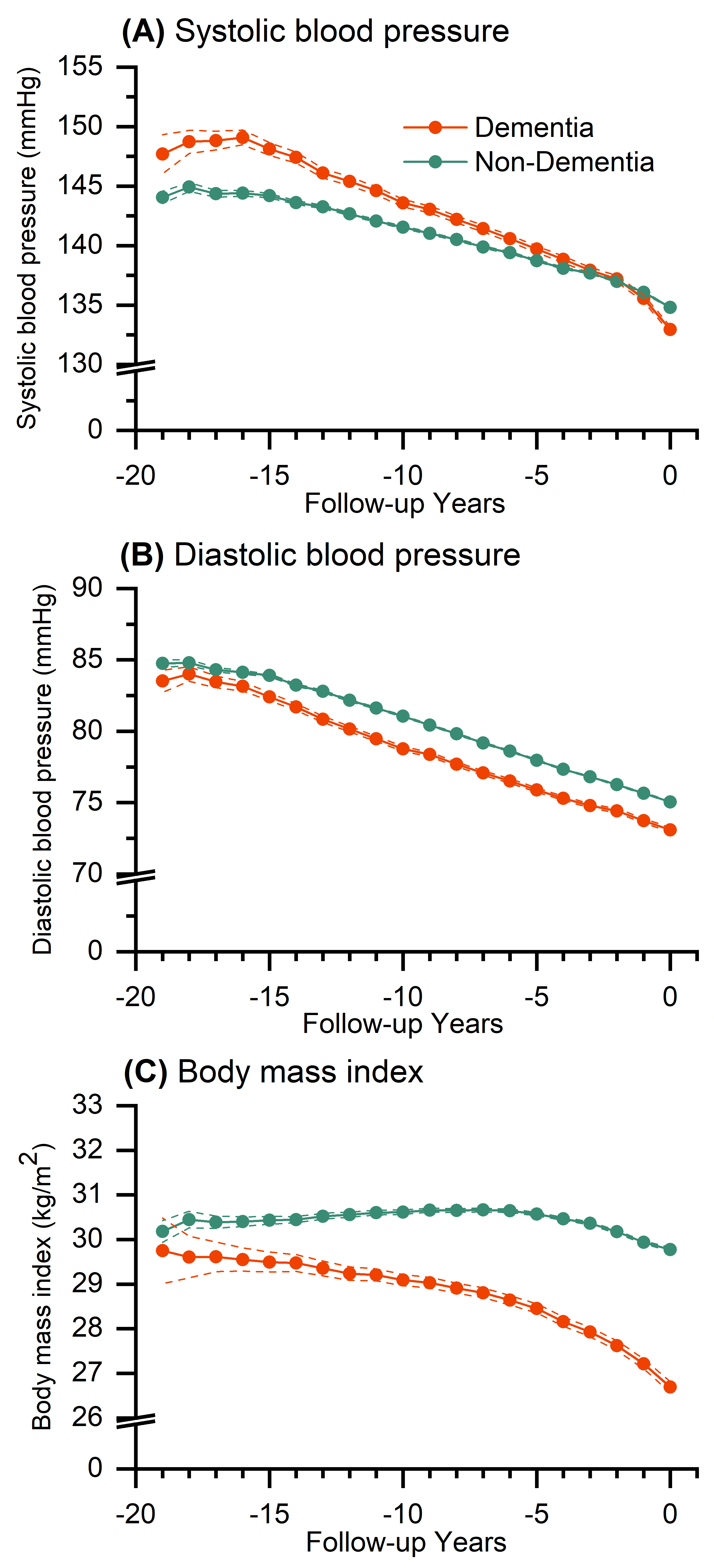


# **Supplementary Figure 3. Trajectories of systolic blood pressure (SBP), diastolic blood pressure (DBP), and body mass index (BMI): crude annual mean.**

Time is expressed as follow-up years with year 0 representing the time of diagnosis of dementia (dementia group) or last contact with healthcare (non-dementia group). Retrospective follow-up time was segmented into one-year time periods for each participant and annual means of SBP, DBP and BMI were plotted as a function of time to create retrospective trajectories. Coloured solid points represent the annual mean and coloured dotted lines represent 95% confidence intervals.


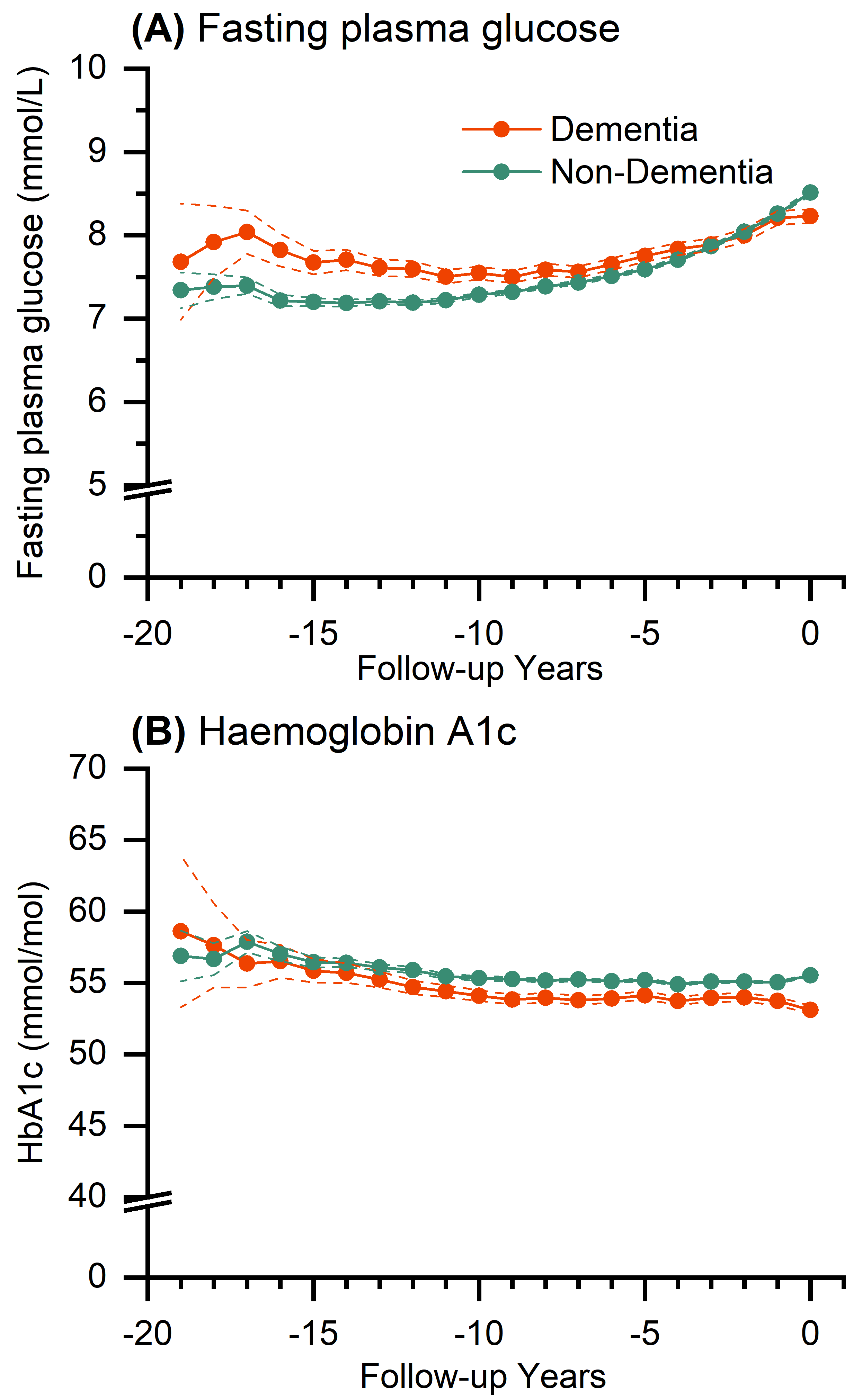


# **Supplementary Figure 4. Trajectories of fasting plasma glucose (FPG) and HbA1c: crude annual mean.**

Time is expressed as follow-up years with year 0 representing the time of diagnosis of dementia (dementia group) or last contact with healthcare (non-dementia group). Retrospective follow-up time was segmented into one-year time periods for each participant and annual means of FPG and HbA1c were plotted as a function of time to create retrospective trajectories. Coloured solid points represent the annual mean and coloured dotted lines represent 95% confidence intervals.


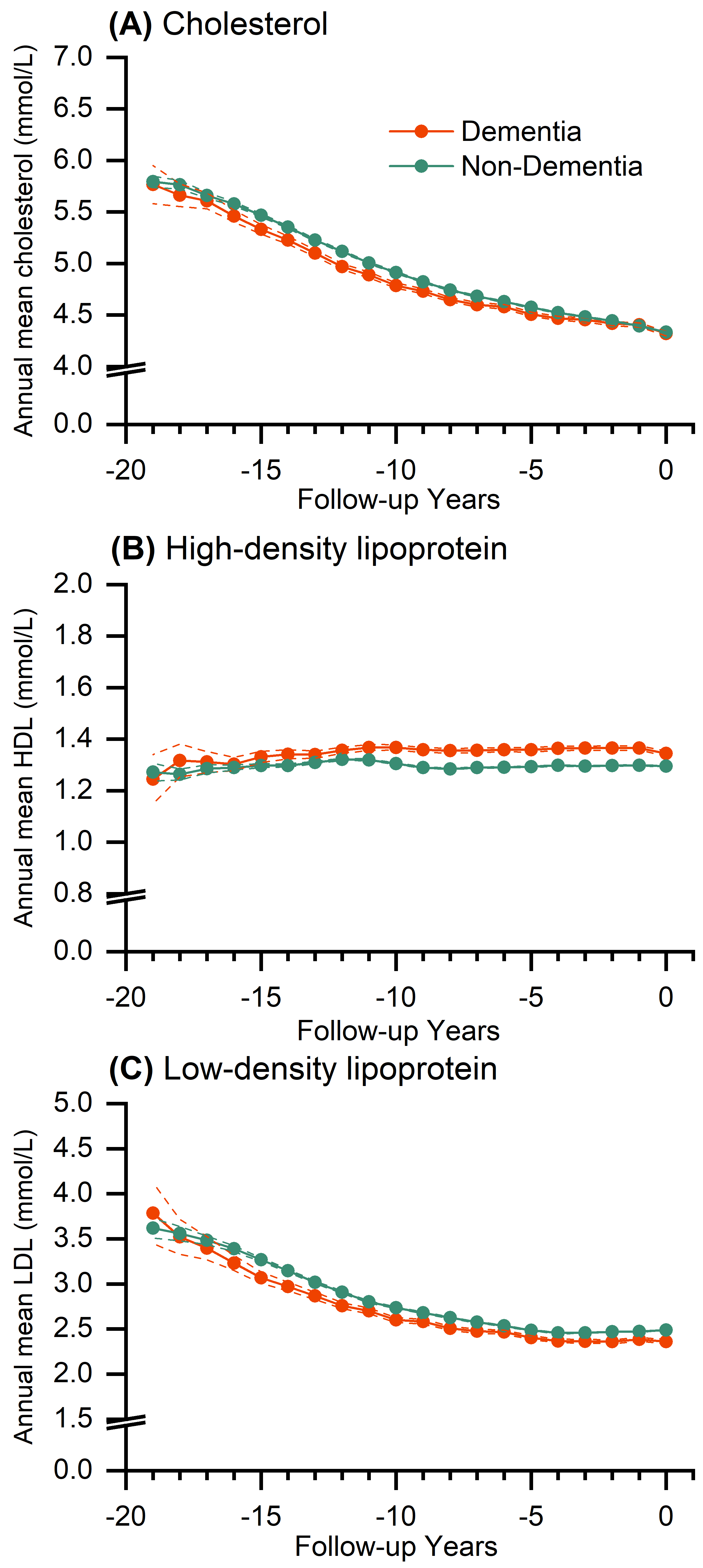


**Supplementary Figure 5. Trajectories of total cholesterol, high-density lipoprotein, and low-density lipoprotein: crude annual mean.**

Time is expressed as follow-up years with year 0 representing the time of diagnosis of dementia (dementia group) or last contact with healthcare (non-dementia group). Retrospective follow-up time was segmented into one-year time periods for each participant and annual means of cholesterol, high-density lipoprotein and low-density lipoprotein were plotted as a function of time to create retrospective trajectories. Coloured solid points represent the annual mean and coloured dotted lines represent 95% confidence intervals.

**Equations**

**(1) Non piecewise linear growth curve model: High-density lipoprotein**

$${CF}_{ij}=\beta_{0}+\beta_{1}{time}_{ij}+\beta_{2}{dementia}_{i}+\beta_{3}{dementia}_{i}{time}_{ij}+ \beta_{4}X_{i}+ \beta_{5}X_{i}{time}_{ij}+\beta_{6}Z_{ij}+u_{0j}+u_{1j}{time}_{ij}+e_{ij}$$

**(2) Non piecewise quadratic growth curve model: Total cholesterol**

$${CF}_{ij}=\beta_{0}+\beta_{1}{time}_{ij}+\beta_{2}{time}_{ij}^{2}+\beta_{3}{dementia}_{i}+\beta_{4}{dementia}_{i}{time}_{ij}+\beta_{5}{dementia}_{i}{time}_{ij}^{2}+ \beta_{6}X_{i}+ \beta_{7}X_{i}{time}_{ij}+\beta_{8}X_{i}{time}_{ij}^{2}+\beta_{9}Z_{ij}+u_{0j}+u_{1j}{time}_{ij}+e_{ij}$$

${CF}_{ij}$**=** Annual mean levels of each cardio-metabolic factor at time *i* for participant *j*,

${time}_{ij}$ **=** Follow-up time in years ranging between -19 to 0, where 0 is the study baseline as participants are traced backwards,

${dementia}_{i}$= dementia status at study baseline (0 = without dementia, 1 = with dementia),

$X_{i}$ = vector for covariates [age (centred at mean age of dementia diagnosis, 82 years), sex, ethnicity, Index of Multiple Deprivation, duration of diabetes (centred at 8 years)] for participant *j* at baseline,

$Z_{ij}$ = vector for time-varying covariates (ever had insulin prescription, number of comorbid conditions) at time *i* for participant *j*,

$u_{0j}$ = random intercept,

$u_{1j}$ = random slope,

$e_{ij}$ = residual.

**(3) Piecewise linear growth curve model: Systolic and diastolic blood pressure**

$${CF}_{ij}=\beta_{0}+\beta_{1}{time}_{1ij}+\beta_{2}{time}_{2ij}+\beta_{3}{time}_{3ij}+\beta_{4}{dementia}_{i}+\beta_{5}{dementia}_{i}{time}_{1ij}+\beta_{6}{dementia}_{i}{time}_{2ij}+\beta_{7}{dementia}_{i}{time}_{3ij}+ \beta_{8}X_{i}+ \beta_{9}X_{i}{time}_{1ij}+\beta_{10}X_{i}{time}_{2ij}+\beta_{11}X_{i}{time}_{3ij}+\beta_{12}Z_{ij}+u_{0j}+u_{1j}{time}_{1ij}+e_{ij}$$

${CF}_{ij}$**=** Annual mean levels of each cardio-metabolic factor at time *i* for participant *j*,

${time}_{1ij}$ **=** 1^st^ time period, follow-up time in years ranging between -2 to 0 for systolic blood pressure, -15 to 0 for diastolic blood pressure, where 0 is the study baseline as participants are traced backwards,

${time}_{2ij}$ **=** 2^nd^ time period, follow-up time in years ranging between -16 to -2 for systolic blood pressure, -18 to -15 for diastolic blood pressure,

${time}_{3ij}$ **=** 3^rd^ time period, follow-up time in years ranging between -19 to -16 for systolic blood pressure, -19 to -18 for diastolic blood pressure,

${dementia}_{i}$= dementia status at study baseline (0 = without dementia, 1 = with dementia),

$X_{i}$ = vector for covariates [age (centred at mean age of dementia diagnosis, 82 years), sex, ethnicity, Index of Multiple Deprivation, duration of diabetes (centred at 8 years)] for participant *j* at baseline,

$Z_{ij}$ = vector for time-varying covariates (ever had insulin prescription, number of comorbid conditions) at time *i* for participant *j*,

$u_{0j}$ = random intercept,

$u_{1j}$ = random slope,

$e_{ij}$ = residual.

**(4) Piecewise non-linear growth curve model: Body mass index (BMI), fasting plasma glucose, HbA1c, and low-density lipoprotein (LDL)**

$${CF}_{ij}=\beta_{0}+\beta_{1}{time}_{1ij}+\beta_{2}{time}_{1ij}^{2}+\beta_{3}{time}_{2ij}+\beta_{4j}{time}_{3ij}+\beta_{5}{dementia}_{i}+\beta_{6}{dementia}_{i}{time}_{1ij}+\beta_{7}{dementia}_{i}{time}_{1ij}^{2}+\beta_{8}{dementia}_{i}{time}_{2ij}+\beta_{9}{dementia}_{i}{time}_{3ij}+ \beta_{10}X_{i}+ \beta_{11}X_{i}{time}_{1ij}+\beta_{12}X_{i}{time}_{1ij}^{2}+\beta_{13}X_{i}{time}_{2ij}+\beta_{14}X_{i}{time}_{3ij}+\beta_{15}Z_{ij}+u_{0j}+u_{1j}{time}_{1ij}+e_{ij}$$

${CF}_{ij}$**=** Annual mean levels of each cardio-metabolic factor at time *i* for participant *j*,

${time}_{1ij}$ **=** 1^st^ time period, follow-up time in years ranging between -11 to 0 for BMI, -17 to 0 for fasting plasma glucose and HbA1c, and -15 to 0 for LDL, where 0 is the study baseline as participants are traced backwards,

${time}_{2ij}$ **=** 2^nd^ time period, follow-up time in years ranging between –19 to -11 for BMI, -19 to -17 for fasting plasma glucose and HbA1c, and -18 to -15 for LDL,

${time}_{3ij}$ **=** 3^rd^ time period, follow-up time in years ranging between -19 to -18 for LDL only,

${dementia}_{i}$= dementia status at study baseline (0 = without dementia, 1 = with dementia),

$X_{i}$ = vector for covariates [age (centred at mean age of dementia diagnosis, 82 years), sex, ethnicity, Index of Multiple Deprivation, duration of diabetes (centred at 8 years)] for participant *j* at baseline,

$Z_{ij}$ = vector for time-varying covariates (ever had insulin prescription, number of comorbid conditions) at time *i* for participant *j*,

$u_{0j}$ = random intercept,

$u_{1j}$ = random slope,

$e_{ij}$ = residual.

**Supplementary Table 7. Ethnicity recoding**

| **Study coding** | **CPRD** | **HES** |
| --- | --- | --- |
| White | White | White |
| Non-White | Black | Black, Caribbean |
|  |  | Black, African |
|  |  | Black, Other |
|  | South Asian | Indian |
|  |  | Pakistani |
|  |  | Bangladeshi |
|  | Mixed | Mixed |
|  | Chinese/Other Asian/Other | Chinese |
|  |  | Other Asian |
|  |  | Other |
| Missing | Missing | Unknown |

# **Supplementary Table 8. Multilevel models for trajectories of cardio-metabolic factors among people with type 2 diabetes before diagnosis of dementia or last contact with healthcare^*^ over 20 years of retrospective follow-up**

|  | **Systolic BP, mmHg** | | | **Diastolic BP, mmHg** | | |
| --- | --- | --- | --- | --- | --- | --- |
| **Fixed effects coefficients** | Model 1 | Model 2 | Time periods, years^†^ | Model 1 | Model 2 | Time periods, years^†^ |
| **Intercept (baseline)** | 135.3^‡^ (135.2, 135.5) | 138.3^‡^ (138.1, 138.5) | 0 | 72.7^‡^ (72.6, 72.7) | 74.0^‡^ (73.9, 74.1) | 0 |
| **1^st^ period** | -1.56^‡^ (-1.63, -1.49) | -1.11^‡^ (-1.22, -0.99) | -2 to 0 | -0.72^‡^ (-0.73, -0.72) | -0.61^‡^ (-0.62, -0.60) | -15 to 0 |
| **1^st^ period^2^** | .. | .. | .. | .. | .. | .. |
| **2^nd^ period** | -0.84^‡^ (-0.85, -0.84) | -0.63^‡^ (-0.65, -0.62) | -16 to -2 | -0.99^‡^ (-1.05, -0.93) | -1.16^‡^ (-1.26, -1.06) | -18 to -15 |
| **3^rd^ period** | -0.61^‡^ (-0.73, -0.48) | -1.01^‡^ (-1.22, -0.80) | -19 to -16 | -0.47^§^ (-0.79, -0.15) | -0.76^§^ (-1.29, -0.22) | -19 to -18 |
| **Dementia case** | -2.29^‡^ (-2.56, -2.02) | -2.01^‡^ (-2.28, -1.74) | 0 | -0.07 (-0.19, 0.04) | 0.13^¶^ (0.02, 0.24) | 0 |
| **Dementia case × 1^st^ period** | -0.40^‡^ (-0.54, -0.25) | -0.46^‡^ (-0.60, -0.31) | -2 to 0 | 0.003 (-0.01, 0.02) | -0.007 (-0.02, 0.01) | -15 to 0 |
| **Dementia case × 1^st^ period^2^** | .. | .. | .. | .. | .. | .. |
| **Dementia case × 2^nd^ period** | -0.17^‡^ (-0.19, -0.16) | -0.16^‡^ (-0.18, -0.14) | -16 to -2 | -0.05 (-0.17, 0.07) | 0.15^¶^ (0.03, 0.27) | -18 to -15 |
| **Dementia case × 3^rd^ period** | 0.35^¶^ (0.08, 0.62) | 0.47^§^ (0.19, 0.74) | -19 to -16 | 0.43 (-0.29, 1.15) | 0.40 (-0.34, 1.13) | -19 to -18 |
|  | **Body mass index, kg/m^2^** | | | **Fasting PG, mmol/L** | | |
|  | Model 1 | Model 2 | Time periods, years^†^ | Model 1 | Model 2 | Time periods, years^†^ |
| **Intercept (baseline)** | 28.0^‡^ (28.0, 28.1) | 28.1^‡^ (28.0, 28.2) | 0 | 8.05^‡^ (8.02, 8.09) | 8.42^‡^ (8.37, 8.48) | 0 |
| **1^st^ period** | -0.33^‡^ (-0.34, -0.33) | -0.36^‡^ (-0.37, -0.35) | -11 to 0 | 0.15^‡^ (0.15, 0.16) | 0.18^‡^ (0.17, 0.19) | -17 to 0 |
| **1^st^ period^2^** | -0.020^‡^ (-0.020, -0.019) | -0.022^‡^ (-0.022, -0.021) | -11 to 0 | 0.006^‡^ (0.005, 0.006) | 0.004^‡^ (0.003, 0.005) | -17 to 0 |
| **2^nd^ period** | 0.06^‡^ (0.05, 0.06) | 0.06^‡^ (0.05, 0.07) | -19 to -11 | -0.05 (-0.16, 0.06) | -0.06 (-0.26, 0.13) | -19 to -17 |
| **3^rd^ period** | .. | .. | .. | .. | .. | .. |
| **Dementia case** | -1.17^‡^ (-1.27, -1.07) | -1.24^‡^ (-1.34, -1.14) | 0 | 0.23^‡^ (0.17, 0.30) | 0.16^‡^ (0.10, 0.22) | 0 |
| **Dementia case × 1^st^ period** | -0.14^‡^ (-0.15, -0.12) | -0.12^‡^ (-0.13, -0.10) | -11 to 0 | -0.02^§^ (-0.04, -0.01) | -0.02^¶^ (-0.03, -0.003) | -17 to 0 |
| **Dementia case × 1^st^ period^2^** | -0.005^‡^ (-0.006, -0.004) | -0.004^‡^ (-0.005, -0.002) | -11 to 0 | -0.0005 (-0.0015, 0.0006) | -0.0015^§^ (-0.0025, -0.0005) | -17 to 0 |
| **Dementia case × 2^nd^ period** | -0.037^‡^ (-0.049, -0.025) | -0.023^‡^ (-0.035, -0.011) | -19 to -11 | 0.04 (-0.18, 0.26) | 0.06 (-0.16, 0.28) | -19 to -17 |
| **Dementia case × 3^rd^ period** | .. | .. | .. | .. | .. | .. |
|  | **HbA1c, mmol/mol** | | | **Cholesterol, mmol/L** | | |
|  | Model 1 | Model 2 | Time periods, years^†^ | Model 1 | Model 2 | Time periods, years^†^ |
| **Intercept (baseline)** | 52.5^‡^ (52.3, 52.6) | 52.9^‡^ (52.7, 53.1) | 0 | 4.58^‡^ (4.57, 4.59) | 4.76^‡^ (4.74, 4.77) | 0 |
| **1^st^ period** | 0.23^‡^ (0.20, 0.26) | 0.31^‡^ (0.26, 0.36) | -17 to 0 | -0.013^‡^ (-0.015, -0.011) | -0.011^‡^ (-0.014, -0.008) | -19 to 0 |
| **1^st^ period^2^** | 0.024^‡^ (0.021, 0.026) | 0.018^‡^ (0.013, 0.022) | -17 to 0 | 0.007^‡^ (0.006, 0.007) | 0.005^‡^ (0.005, 0.006) | -19 to 0 |
| **2^nd^ period** | 0.20 (-0.41, 0.82) | 2.62^§^ (1.05, 4.19) | -19 to -17 | .. | .. | .. |
| **3^rd^ period** | .. | .. | .. | .. | .. | .. |
| **Dementia case** | 0.90^‡^ (0.64, 1.16) | 0.68^‡^ (0.43, 0.94) | 0 | 0.026^§^ (0.008, 0.044) | 0.068^‡^ (0.050, 0.086) | 0 |
| **Dementia case × 1^st^ period** | -0.08^¶^ (-0.15, -0.02) | -0.03 (-0.10, 0.03) | -17 to 0 | 0.011^‡^ (0.007, 0.015) | 0.004^¶^ (0.0004, 0.008) | -19 to 0 |
| **Dementia case × 1^st^ period^2^** | -0.003 (-0.008, 0.001) | -0.0004 (-0.005, 0.004) | -17 to 0 | 0.0008^‡^ (0.0005, 0.0011) | 0.0005^‡^ (0.0002, 0.0007) | -19 to 0 |
| **Dementia case × 2^nd^ period** | -1.45^¶^ (-2.66, -0.24) | -1.98^§^ (-3.21, -0.74) | -19 to -17 | .. | .. | .. |
| **Dementia case × 3^rd^ period** | .. | .. | .. | .. | .. | .. |
|  | **HDL, mmol/L** | | | **LDL, mmol/L** | | |
|  | Model 1 | Model 2 | Time periods, years^†^ | Model 1 | Model 2 | Time periods, years^†^ |
| **Intercept (baseline)** | 1.469^‡^ (1.466, 1.472) | 1.503^‡^ (1.497, 1.508) | 0 | 2.58^‡^ (2.57, 2.59) | 2.76^‡^ (2.75, 2.78) | 0 |
| **1^st^ period** | 0.0004^¶^ (0.0001, 0.0007) | 0.0035^‡^ (0.0030, 0.0040) | -19 to 0 | 0.036^‡^ (0.033, 0.038) | 0.039^‡^ (0.035, 0.042) | -15 to 0 |
| **1^st^ period^2^** | .. | .. | .. | 0.008^‡^ (0.008, 0.008) | 0.008^‡^ (0.007, 0.008) | -15 to 0 |
| **2^nd^ period** | .. | .. | .. | -0.17^‡^ (-0.19, -0.16) | -0.11^‡^ (-0.14, -0.07) | -18 to -15 |
| **3^rd^ period** | .. | .. | .. | -0.10 (-0.21, 0.02) | -0.47^‡^ (-0.71, -0.23) | -19 to -18 |
| **Dementia case** | 0.001 (-0.005, 0.007) | 0.006 (-0.001, 0.012) | 0 | -0.07^‡^ (-0.09, -0.05) | -0.04^‡^ (-0.06, -0.02) | 0 |
| **Dementia case × 1^st^ period** | -0.0002 (-0.0008, 0.0004) | -0.0002 (-0.0008, 0.0004) | -19 to 0 | -0.016^‡^ (-0.021, -0.011) | -0.019^‡^ (-0.024, -0.015) | -15 to 0 |
| **Dementia case × 1^st^ period^2^** | .. | .. | .. | -0.001^‡^ (-0.001, -0.001) | -0.001^‡^ (-0.001, -0.001) | -15 to 0 |
| **Dementia case × 2^nd^ period** | .. | .. | .. | -0.04^¶^ (-0.07, -0.01) | -0.02 (-0.05, 0.01) | -18 to -15 |
| **Dementia case × 3^rd^ period** | .. | .. | .. | -0.39^§^ (-0.62, -0.16) | -0.38^§^ (-0.62, -0.14) | -19 to -18 |

Abbreviations: BP - blood pressure; PG, - plasma glucose; HbA1c - haemoglobin A1c; HDL - high-density lipoprotein; LDL - low-density lipoprotein.

Regression coefficients (95% confidence intervals) were derived from piecewise linear and non-linear growth curve models with random intercept and slope, and represent differences in annual changes in cardio-metabolic factors between dementia and non-dementia cases during distinct time periods over the 20-year follow-up. Time was expressed as follow-up time in years for up to three time periods. For HDL and total cholesterol, non-piecewise models were fitted (with a single time period).

^*^Study baseline is established as the date of diagnosis of dementia (dementia group) or last contact with healthcare (non-dementia group). Participants were then traced backwards in time.

^†^Time periods indicate the segmentation of the 20-year retrospective follow-up time ranging from -19 to 0 years (i.e., -19 refers to ‘19 years before year 0’), where the 1^st^ time period is the closest to baseline, followed by a 2^nd^ time period, and finally a 3^rd^ time period which is the furthest away from baseline.

Model 1 is adjusted for age and sex.

Model 2 is adjusted for age, sex, ethnicity, smoking status, Index of Multiple Deprivation quintiles, duration of diabetes, if ever prescribed insulin, and number of comorbid conditions.

Patient ID and time (non-piecewise), or the time period closest to baseline (piecewise) was modelled as random effects in all models.

^‡^ *p*<0.001, ^§^ *p*<0.01, ^¶^ *p*<0.05.

# **Supplementary Table 9. Annual differences (95% confidence interval) in levels of systolic blood pressure, diastolic blood pressure, body mass index, fasting plasma glucose, and haemoglobin A1c for participants with dementia relative to participants without dementia**

| **Year^*^** | **Systolic blood pressure** | | **Diastolic blood pressure** | | **Body mass index** | | **Fasting plasma glucose** | | **Haemoglobin A1c** | |
| --- | --- | --- | --- | --- | --- | --- | --- | --- | --- | --- |
|  | **Difference in mmHg/year** | **p-value**^†^ | **Difference in mmHg/year** | **p-value**^†^ | **Difference in kg/m^2^** | **p-value**^†^ | **Difference in mmol/L** | **p-value**^†^ | **Difference in mmol/mol** | **p-value**^†^ |
| **-19** | -0.21 (-1.02, 0.61) | 0.615 | -0.61 (-1.31, 0.09) | 0.085 | -0.21 (-0.33, -0.09) | 0.001 | -0.09 (-0.53, 0.35) | 0.688 | 5.08 (2.57, 7.59) | <0.001 |
| **-18** | 0.26 (-0.30, 0.82) | 0.368 | -0.22 (-0.59, 0.16) | 0.258 | -0.23 (-0.35, -0.12) | <0.001 | -0.03 (-0.26, 0.20) | 0.798 | 3.10 (1.73, 4.48) | <0.001 |
| **-17** | 0.72 (0.39, 1.06) | <0.001 | -0.07 (-0.34, 0.21) | 0.630 | -0.26 (-0.37, -0.15) | <0.001 | 0.03 (-0.08, 0.14) | 0.611 | 1.13 (0.38, 1.88) | 0.003 |
| **-16** | 1.19 (0.94, 1.43) | <0.001 | 0.08 (-0.11, 0.27) | 0.399 | -0.28 (-0.38, -0.18) | <0.001 | 0.06 (-0.03, 0.16) | 0.206 | 1.11 (0.46, 1.76) | 0.001 |
| **-15** | 1.02 (0.79, 1.26) | <0.001 | 0.23 (0.07, 0.40) | 0.006 | -0.30 (-0.40, -0.20) | <0.001 | 0.09 (0.01, 0.17) | 0.025 | 1.09 (0.53, 1.65) | <0.001 |
| **-14** | 0.86 (0.64, 1.08) | <0.001 | 0.23 (0.07, 0.38) | 0.004 | -0.33 (-0.42, -0.23) | <0.001 | 0.12 (0.05, 0.18) | <0.001 | 1.06 (0.58, 1.55) | <0.001 |
| **-13** | 0.70 (0.49, 0.91) | <0.001 | 0.22 (0.08, 0.36) | 0.003 | -0.35 (-0.44, -0.26) | <0.001 | 0.14 (0.08, 0.19) | <0.001 | 1.04 (0.63, 1.46) | <0.001 |
| **-12** | 0.53 (0.33, 0.74) | <0.001 | 0.21 (0.08, 0.34) | 0.002 | -0.37 (-0.47, -0.28) | <0.001 | 0.16 (0.11, 0.20) | <0.001 | 1.02 (0.66, 1.38) | <0.001 |
| **-11** | 0.37 (0.18, 0.56) | <0.001 | 0.21 (0.08, 0.33) | 0.001 | -0.40 (-0.49, -0.30) | <0.001 | 0.17 (0.13, 0.22) | <0.001 | 0.99 (0.68, 1.31) | <0.001 |
| **-10** | 0.21 (0.02, 0.39) | 0.028 | 0.20 (0.09, 0.31) | 0.001 | -0.44 (-0.53, -0.35) | <0.001 | 0.19 (0.15, 0.23) | <0.001 | 0.97 (0.69, 1.25) | <0.001 |
| **-9** | 0.04 (-0.14, 0.23) | 0.626 | 0.19 (0.09, 0.30) | <0.001 | -0.48 (-0.57, -0.40) | <0.001 | 0.20 (0.16, 0.24) | <0.001 | 0.94 (0.70, 1.19) | <0.001 |
| **-8** | -0.12 (-0.30, 0.06) | 0.190 | 0.19 (0.09, 0.28) | <0.001 | -0.54 (-0.63, -0.45) | <0.001 | 0.21 (0.17, 0.24) | <0.001 | 0.92 (0.69, 1.15) | <0.001 |
| **-7** | -0.28 (-0.46, -0.11) | 0.002 | 0.18 (0.09, 0.27) | <0.001 | -0.60 (-0.69, -0.52) | <0.001 | 0.21 (0.17, 0.25) | <0.001 | 0.89 (0.68, 1.10) | <0.001 |
| **-6** | -0.44 (-0.62, -0.27) | <0.001 | 0.17 (0.08, 0.26) | <0.001 | -0.67 (-0.76, -0.59) | <0.001 | 0.21 (0.17, 0.25) | <0.001 | 0.86 (0.66, 1.07) | <0.001 |
| **-5** | -0.61 (-0.79, -0.43) | <0.001 | 0.17 (0.08, 0.25) | <0.001 | -0.75 (-0.83, -0.66) | <0.001 | 0.21 (0.17, 0.25) | <0.001 | 0.84 (0.64, 1.03) | <0.001 |
| **-4** | -0.77 (-0.95, -0.59) | <0.001 | 0.16 (0.07, 0.25) | 0.001 | -0.83 (-0.92, -0.75) | <0.001 | 0.21 (0.17, 0.25) | <0.001 | 0.81 (0.62, 0.99) | <0.001 |
| **-3** | -0.93 (-1.12, -0.75) | <0.001 | 0.15 (0.06, 0.24) | 0.001 | -0.92 (-1.01, -0.84) | <0.001 | 0.20 (0.16, 0.24) | <0.001 | 0.78 (0.59, 0.97) | <0.001 |
| **-2** | -1.10 (-1.29, -0.90) | <0.001 | 0.15 (0.05, 0.24) | 0.004 | -1.02 (-1.11, -0.93) | <0.001 | 0.19 (0.14, 0.24) | <0.001 | 0.75 (0.55, 0.94) | <0.001 |
| **-1** | -1.56 (-1.74, -1.37) | <0.001 | 0.14 (0.03, 0.24) | 0.010 | -1.13 (-1.22, -1.04) | <0.001 | 0.18 (0.12, 0.23) | <0.001 | 0.72 (0.50, 0.93) | <0.001 |
| **0** | -2.01 (-2.28, -1.74) | <0.001 | 0.13 (0.02, 0.24) | 0.022 | -1.24 (-1.34, -1.14) | <0.001 | 0.16 (0.10, 0.22) | <0.001 | 0.68 (0.43, 0.94) | <0.001 |

^*^Study baseline is established as the date of diagnosis of dementia (dementia group) or last contact with healthcare (non-dementia group). Participants were then traced backwards in time. As such, -19 year refers to ‘19 years before year 0’.

^†^ Differences presented are for the dementia group relative to the non-dementia group, with p-value obtained through the Wald-test.

# **Supplementary Table 10. Annual differences (95% confidence interval) in levels of cholesterol, low-density lipoprotein, and high-density lipoprotein for participants with dementia relative to participants without dementia**

| **Year** | **Cholesterol** | | **Low-density lipoprotein** | | **High-density lipoprotein** | |
| --- | --- | --- | --- | --- | --- | --- |
|  | **Difference in mmol/L** | **p-value**^†^ | **Difference in mmol/L** | **p-value**^†^ | **Difference in mmol/L** | **p-value**^†^ |
| **-19** | 0.16 (0.11, 0.21) | <0.001 | 0.46 (0.23, 0.69) | <0.001 | 0.010 (0.000, 0.020) | 0.047 |
| **-18** | 0.14 (0.10, 0.19) | <0.001 | 0.09 (-0.01, 0.18) | 0.075 | 0.010 (0.000, 0.019) | 0.040 |
| **-17** | 0.13 (0.09, 0.17) | <0.001 | 0.07 (0.00, 0.13) | 0.052 | 0.010 (0.001, 0.018) | 0.034 |
| **-16** | 0.12 (0.09, 0.15) | <0.001 | 0.05 (0.00, 0.09) | 0.038 | 0.009 (0.001, 0.018) | 0.029 |
| **-15** | 0.11 (0.08, 0.14) | <0.001 | 0.03 (-0.01, 0.06) | 0.156 | 0.009 (0.001, 0.017) | 0.024 |
| **-14** | 0.10 (0.07, 0.13) | <0.001 | 0.04 (0.00, 0.07) | 0.023 | 0.009 (0.001, 0.016) | 0.020 |
| **-13** | 0.09 (0.07, 0.11) | <0.001 | 0.04 (0.02, 0.07) | 0.001 | 0.009 (0.002, 0.016) | 0.016 |
| **-12** | 0.08 (0.06, 0.10) | <0.001 | 0.05 (0.03, 0.07) | <0.001 | 0.008 (0.002, 0.015) | 0.013 |
| **-11** | 0.08 (0.06, 0.10) | <0.001 | 0.05 (0.03, 0.07) | <0.001 | 0.008 (0.002, 0.014) | 0.011 |
| **-10** | 0.07 (0.05, 0.09) | <0.001 | 0.05 (0.04, 0.07) | <0.001 | 0.008 (0.002, 0.014) | 0.009 |
| **-9** | 0.07 (0.05, 0.08) | <0.001 | 0.05 (0.04, 0.07) | <0.001 | 0.008 (0.002, 0.013) | 0.008 |
| **-8** | 0.06 (0.05, 0.08) | <0.001 | 0.05 (0.04, 0.07) | <0.001 | 0.008 (0.002, 0.013) | 0.008 |
| **-7** | 0.06 (0.05, 0.07) | <0.001 | 0.05 (0.03, 0.06) | <0.001 | 0.007 (0.002, 0.013) | 0.008 |
| **-6** | 0.06 (0.05, 0.07) | <0.001 | 0.04 (0.03, 0.06) | <0.001 | 0.007 (0.002, 0.012) | 0.009 |
| **-5** | 0.06 (0.04, 0.07) | <0.001 | 0.03 (0.02, 0.05) | <0.001 | 0.007 (0.002, 0.012) | 0.012 |
| **-4** | 0.06 (0.04, 0.07) | <0.001 | 0.02 (0.01, 0.04) | 0.001 | 0.007 (0.001, 0.012) | 0.016 |
| **-3** | 0.06 (0.05, 0.07) | <0.001 | 0.01 (0.00, 0.02) | 0.145 | 0.006 (0.001, 0.012) | 0.023 |
| **-2** | 0.06 (0.05, 0.08) | <0.001 | 0.00 (-0.02, 0.01) | 0.568 | 0.006 (0.000, 0.012) | 0.034 |
| **-1** | 0.06 (0.05, 0.08) | <0.001 | -0.02 (-0.04, 0.00) | 0.014 | 0.006 (0.000, 0.012) | 0.050 |
| **0** | 0.07 (0.05, 0.09) | <0.001 | -0.04 (-0.06, -0.02) | <0.001 | 0.006 (-0.001, 0.012) | 0.072 |

^*^Study baseline is established as the date of diagnosis of dementia (dementia group) or last contact with healthcare (non-dementia group). Participants were then traced backwards in time. As such, -19 year refers to ‘19 years before year 0’.

^†^ Differences presented are for the dementia group relative to the non-dementia group, with p-value obtained through the Wald-test.

# **Supplementary Table 11. Multilevel models for differences in trajectories of cardio-metabolic factors between participants with type 2 diabetes with and without dementia (including probable cognitive impairment) over 20 years of retrospective follow-up**

| **Fixed effects coefficients** | **Systolic BP, mmHg** | **Time periods^*^, years** | **Diastolic BP, mmHg** | **Time periods^*^, years** |
| --- | --- | --- | --- | --- |
| **Intercept (baseline)** | 138.3^†^ (138.1, 138.5) | 0 | 74.0^†^ (73.9, 74.1) | 0 |
| **1^st^ period** | -1.11^†^ (-1.23, -1.00) | -2 to 0 | -0.61^†^ (-0.62, -0.60) | -15 to 0 |
| **1^st^ period^2^** | .. | .. | .. | .. |
| **2^nd^ period** | -0.63^†^ (-0.65, -0.62) | -16 to -2 | -1.16^†^ (-1.26, -1.06) | -18 to -15 |
| **3^rd^ period** | -1.03^†^ (-1.24, -0.82) | -19 to -16 | -0.76^‡^ (-1.29, -0.22) | -19 to -18 |
| **Dementia case** | -1.96^†^ (-2.23, -1.70) | 0 | 0.12 (-0.004, 0.23) | 0 |
| **Dementia case × 1^st^ period** | -0.42^†^ (-0.57, -0.28) | -2 to 0 | -0.007 (-0.02, 0.01) | -15 to 0 |
| **Dementia case × 1^st^ period^2^** | .. | .. | .. | .. |
| **Dementia case × 2^nd^ period** | -0.16^†^ (-0.18, -0.14) | -16 to -2 | 0.15^§^ (0.03, 0.27) | -18 to -15 |
| **Dementia case × 3^rd^ period** | 0.47^†^ (0.20, 0.74) | -19 to -16 | 0.39 (-0.34, 1.12) | -19 to -18 |
| **Fixed effects coefficients** | **Body mass index, kg/m^2^** | **Time periods^*^, years** | **Fasting PG, mmol/L** | **Time periods^*^, years** |
| **Intercept (baseline)** | 28.1^†^ (28.0, 28.2) | 0 | 8.42^†^ (8.37, 8.48) | 0 |
| **1^st^ period** | -0.36^†^ (-0.37, -0.35) | -11 to 0 | 0.18^†^ (0.17, 0.19) | -17 to 0 |
| **1^st^ period^2^** | -0.021^†^ (-0.022, -0.021) | -11 to 0 | 0.004^†^ (0.003, 0.005) | -17 to 0 |
| **2^nd^ period** | 0.06^†^ (0.05, 0.07) | -19 to -11 | -0.06 (-0.25, 0.13) | -19 to -17 |
| **3^rd^ period** | .. | .. | .. | .. |
| **Dementia case** | -1.19^†^ (-1.29, -1.10) | 0 | 0.14^†^ (0.08, 0.21) | 0 |
| **Dementia case × 1^st^ period** | -0.11^†^ (-0.13, -0.10) | -11 to 0 | -0.02^§^ (-0.03, -0.003) | -17 to 0 |
| **Dementia case × 1^st^ period^2^** | -0.004^†^ (-0.005, -0.002) | -11 to 0 | -0.0015^‡^ (-0.0025, -0.0005) | -17 to 0 |
| **Dementia case × 2^nd^ period** | -0.024^†^ (-0.035, -0.012) | -19 to -11 | 0.05 (-0.17, 0.27) | -19 to -17 |
| **Dementia case × 3^rd^ period** | .. | .. | .. | .. |
| **Fixed effects coefficients** | **HbA1c, mmol/mol** | **Time periods^*^, years** | **Cholesterol, mmol/L** | **Time periods^*^, years** |
| **Intercept (baseline)** | 52.9^†^ (52.7, 53.1) | 0 | 4.76^†^ (4.74, 4.77) | 0 |
| **1^st^ period** | 0.31^†^ (0.26, 0.36) | -17 to 0 | -0.011^†^ (-0.014, -0.008) | -19 to 0 |
| **1^st^ period^2^** | 0.018^†^ (0.013, 0.023) | -17 to 0 | 0.005^†^ (0.005, 0.006) | -19 to 0 |
| **2^nd^ period** | 2.59^‡^ (1.01, 4.16) | -19 to -17 | .. | .. |
| **3^rd^ period** | .. | .. | .. | .. |
| **Dementia case** | 0.62^†^ (0.37, 0.86) | 0 | 0.065^†^ (0.047, 0.082) | 0 |
| **Dementia case × 1^st^ period** | -0.03 (-0.09, 0.03) | -17 to 0 | 0.005^§^ (0.0009, 0.009) | -19 to 0 |
| **Dementia case × 1^st^ period^2^** | -0.0003 (-0.005, 0.004) | -17 to 0 | 0.0005^†^ (0.0002, 0.0007) | -19 to 0 |
| **Dementia case × 2^nd^ period** | -1.77^‡^ (-2.98, -0.55) | -19 to -17 | .. | .. |
| **Dementia case × 3^rd^ period** | .. | .. | .. | .. |
|  | **HDL, mmol/L** | **Time periods^*^, years** | **LDL, mmol/L** | **Time periods^*^, years** |
| **Intercept (baseline)** | 1.502^†^ (1.497, 1.508) | 0 | 2.76^†^ (2.75, 2.78) | 0 |
| **1^st^ period** | 0.0035^†^ (0.0030, 0.0040) | -19 to 0 | 0.038^†^ (0.035, 0.042) | -15 to 0 |
| **1^st^ period^2^** | .. | .. | 0.008^†^ (0.007, 0.008) | -15 to 0 |
| **2^nd^ period** | .. | .. | -0.11^†^ (-0.14, -0.07) | -18 to -15 |
| **3^rd^ period** | .. | .. | -0.47^†^ (-0.71, -0.23) | -19 to -18 |
| **Dementia case** | 0.006 (-0.0001, 0.012) | 0 | -0.04^‡^ (-0.06, -0.02) | 0 |
| **Dementia case × 1^st^ period** | -0.0001 (-0.0007, 0.0005) | -19 to 0 | -0.018^†^ (-0.023, -0.013) | -15 to 0 |
| **Dementia case × 1^st^ period^2^** | .. | .. | -0.001^†^ (-0.001, -0.001) | -15 to 0 |
| **Dementia case × 2^nd^ period** | .. | .. | -0.02 (-0.05, 0.01) | -18 to -15 |
| **Dementia case × 3^rd^ period** | .. | .. | -0.38^‡^ (-0.62, -0.14) | -19 to -18 |

Abbreviations: BP - blood pressure; PG, - plasma glucose; HbA1c - haemoglobin A1c; HDL - high-density lipoprotein; LDL - low-density lipoprotein.

Regression coefficients (95% confidence intervals) were derived from piecewise linear and non-linear growth curve models with random intercept and slope, and represent differences in annual changes in cardio-metabolic factors between dementia and non-dementia cases during distinct time periods over the 20-year follow-up. Time was expressed as years for up to three time periods. For HDL and total cholesterol, non-piecewise models were fitted (with a single time period).

^*^Study baseline is established as the date of diagnosis of dementia (dementia group) or last contact with healthcare (non-dementia group). Participants were then traced backwards in time. Time periods indicate the segmentation of the 20-year retrospective follow-up time ranging from -19 to 0 years (i.e., -19 refers to ‘19 years before year 0’), where the 1^st^ time period is the closest to baseline, followed by a 2^nd^ time period, and finally a 3^rd^ time period which is the furthest away from baseline.

Model is adjusted for age, sex, ethnicity, smoking status, Index of Multiple Deprivation quintiles, duration of diabetes, if ever prescribed insulin, stroke, and myocardial infarction. Patient ID and time (non-piecewise), or the time period closest to baseline (piecewise) was modelled as random effects.

Level of significance at ^†^*p*<0.001, unless otherwise indicated, ^‡^ *p*<0.01, ^§^ *p*<0.05.

# **Supplementary Table 12.** **Multilevel models for differences in trajectories of cardio-metabolic factors between people with type 2 diabetes with and without dementia (including referrals to memory clinic) over 20 years of retrospective follow-up**

| **Fixed effects coefficients** | **Systolic BP, mmHg** | **Time periods^*^, years** | **Diastolic BP, mmHg** | **Time periods^*^, years** |
| --- | --- | --- | --- | --- |
| **Intercept (baseline)** | 138.4^†^ (138.1, 138.6) | 0 | 74.0^†^ (73.9, 74.1) | 0 |
| **1^st^ period** | -1.11^†^ (-1.22, -0.99) | -2 to 0 | -0.61^†^ (-0.62, -0.60) | -15 to 0 |
| **1^st^ period^2^** | .. | .. | .. | .. |
| **2^nd^ period** | -0.63^†^ (-0.65, -0.62) | -16 to -2 | -1.17^†^ (-1.27, -1.07) | -18 to -15 |
| **3^rd^ period** | -1.03^†^ (-1.24, -0.82) | -19 to -16 | -0.76^‡^ (-1.30, -0.22) | -19 to -18 |
| **Dementia case** | -1.92^†^ (-2.19, -1.66) | 0 | 0.11 (-0.001, 0.22) | 0 |
| **Dementia case × 1^st^ period** | -0.41^†^ (-0.55, -0.27) | -2 to 0 | -0.008 (-0.02, 0.01) | -15 to 0 |
| **Dementia case × 1^st^ period^2^** | .. | .. | .. | .. |
| **Dementia case × 2^nd^ period** | -0.16^†^ (-0.17, -0.14) | -16 to -2 | 0.15^§^ (0.04, 0.27) | -18 to -15 |
| **Dementia case × 3^rd^ period** | 0.46^†^ (0.18, 0.73) | -19 to -16 | 0.39 (-0.34, 1.12) | -19 to -18 |
| **Fixed effects coefficients** | **Body mass index, kg/m^2^** | **Time periods^*^, years** | **Fasting PG, mmol/L** | **Time periods^*^, years** |
| **Intercept (baseline)** | 28.1^†^ (28.0, 28.2) | 0 | 8.42^†^ (8.37, 8.48) | 0 |
| **1^st^ period** | -0.36^†^ (-0.37, -0.35) | -11 to 0 | 0.18^†^ (0.17, 0.19) | -17 to 0 |
| **1^st^ period^2^** | -0.021^†^ (-0.022, -0.021) | -11 to 0 | 0.004^†^ (0.003, 0.005) | -17 to 0 |
| **2^nd^ period** | 0.06^†^ (0.05, 0.07) | -19 to -11 | -0.07 (-0.26, 0.13) | -19 to -17 |
| **3^rd^ period** |  | .. | .. | .. |
| **Dementia case** | -1.18^†^ (-1.27, -1.08) | 0 | 0.14^†^ (0.08, 0.21) | 0 |
| **Dementia case × 1^st^ period** | -0.11^†^ (-0.12, -0.10) | -11 to 0 | -0.02^§^ (-0.03, -0.002) | -17 to 0 |
| **Dementia case × 1^st^ period^2^** | -0.003^†^ (-0.005, -0.002) | -11 to 0 | -0.0014^‡^ (-0.0024, -0.0004) | -17 to 0 |
| **Dementia case × 2^nd^ period** | -0.023^†^ (-0.034, -0.011) | -19 to -11 | 0.06 (-0.16, 0.28) | -19 to -17 |
| **Dementia case × 3^rd^ period** | .. | .. | .. | .. |
| **Fixed effects coefficients** | **HbA1c, mmol/mol** | **Time periods^*^, years** | **Cholesterol, mmol/L** | **Time periods^*^, years** |
| **Intercept (baseline)** | 52.9^†^ (52.7, 53.1) | 0 | 4.76^†^ (4.74, 4.77) | 0 |
| **1^st^ period** | 0.31^†^ (0.25, 0.36) | -17 to 0 | -0.011^†^ (-0.015, -0.008) | -19 to 0 |
| **1^st^ period^2^** | 0.018^†^ (0.013, 0.022) | -17 to 0 | 0.005^†^ (0.005, 0.006) | -19 to 0 |
| **2^nd^ period** | 2.57^‡^ (1.00, 4.14) | -19 to -17 | .. | .. |
| **3^rd^ period** | .. | .. | .. | .. |
| **Dementia case** | 0.62^†^ (0.37, 0.87) | 0 | 0.063^†^ (0.046, 0.081) | 0 |
| **Dementia case × 1^st^ period** | -0.02 (-0.08, 0.04) | -17 to 0 | 0.005^§^ (0.0012, 0.009) | -19 to 0 |
| **Dementia case × 1^st^ period^2^** | -0.0004 (-0.004, 0.005) | -17 to 0 | 0.0005^†^ (0.0003, 0.0008) | -19 to 0 |
| **Dementia case × 2^nd^ period** | -1.73^‡^ (-2.96, -0.51) | -19 to -17 | .. | .. |
| **Dementia case × 3^rd^ period** | .. | .. | .. | .. |
|  | **HDL, mmol/L** | **Time periods^*^, years** | **LDL, mmol/L** | **Time periods^*^, years** |
| **Intercept (baseline)** | 1.502^†^ (1.497, 1.508) | 0 | 2.76^†^ (2.74, 2.78) | 0 |
| **1^st^ period** | 0.0035^†^ (0.0030, 0.0040) | -19 to 0 | 0.038^†^ (0.034, 0.042) | -15 to 0 |
| **1^st^ period^2^** | .. | .. | 0.008^†^ (0.007, 0.008) | -15 to 0 |
| **2^nd^ period** | .. | .. | -0.11^†^ (-0.14, -0.07) | -18 to -15 |
| **3^rd^ period** | .. | .. | -0.47^†^ (-0.71, -0.23) | -19 to -18 |
| **Dementia case** | 0.006 (-0.00004, 0.012) | 0 | -0.04^‡^ (-0.06, -0.02) | 0 |
| **Dementia case × 1^st^ period** | -0.00004 (-0.0006, 0.0006) | -19 to 0 | -0.018^†^ (-0.023, -0.014) | -15 to 0 |
| **Dementia case × 1^st^ period^2^** | .. | .. | -0.001^†^ (-0.001, -0.001) | -15 to 0 |
| **Dementia case × 2^nd^ period** | .. | .. | -0.02 (-0.05, 0.01) | -18 to -15 |
| **Dementia case × 3^rd^ period** | .. | .. | -0.38^‡^ (-0.62, -0.14) | -19 to -18 |

Abbreviations: BP - blood pressure; PG, - plasma glucose; HbA1c - haemoglobin A1c; HDL - high-density lipoprotein; LDL - low-density lipoprotein.

Regression coefficients (95% confidence intervals) were derived from piecewise linear and non-linear growth curve models with random intercept and slope, and represent differences in annual changes in cardio-metabolic factors between dementia and non-dementia cases during distinct time periods over the 20-year follow-up. Time was expressed as years for up to three time periods. For HDL and total cholesterol, non-piecewise models were fitted (with a single time period).

^*^Study baseline is established as the date of diagnosis of dementia (dementia group) or last contact with healthcare (non-dementia group). Participants were then traced backwards in time. Time periods indicate the segmentation of the 20-year retrospective follow-up time ranging from -19 to 0 years (i.e., -19 refers to ‘19 years before year 0’), where the 1^st^ time period is the closest to baseline, followed by a 2^nd^ time period, and finally a 3^rd^ time period which is the furthest away from baseline.

Model is adjusted for age, sex, ethnicity, smoking status, Index of Multiple Deprivation quintiles, duration of diabetes, if ever prescribed insulin, stroke, and myocardial infarction. Patient ID and time (non-piecewise), or the time period closest to baseline (piecewise) was modelled as random effects.

Level of significance at ^†^*p*<0.001, unless otherwise indicated, ^‡^ *p*<0.01, ^§^ *p*<0.05.

# **Supplementary Table 13. Multilevel models for differences in trajectories of cardio-metabolic factors between people with type 2 diabetes with and without dementia over 20 years of retrospective follow-up: adjusting for stroke and myocardial infarction**

| **Fixed effects coefficients** | **Systolic BP, mmHg** | **Time periods^*^, years** | **Diastolic BP, mmHg** | **Time periods^*^, years** |
| --- | --- | --- | --- | --- |
| **Intercept (baseline)** | 136.1^†^ (135.9, 136.3) | 0 | 73.1^†^ (73.0, 73.2) | 0 |
| **1^st^ period** | -1.31^†^ (-1.42, -1.20) | -2 to 0 | -0.68^†^ (-0.69, -0.67) | -15 to 0 |
| **1^st^ period^2^** | .. | .. | .. | .. |
| **2^nd^ period** | -0.81^†^ (-0.83, -0.80) | -16 to -2 | -1.19^†^ (-1.29, -1.09) | -18 to -15 |
| **3^rd^ period** | -1.01^†^ (-1.22, -0.80) | -19 to -16 | -0.80^‡^ (-1.34, -0.27) | -19 to -18 |
| **Dementia case** | -2.04^†^ (-2.31, -1.77) | 0 | 0.10 (-0.02, 0.21) | 0 |
| **Dementia case × 1^st^ period** | -0.44^†^ (-0.59, -0.30) | -2 to 0 | -0.01 (-0.03, 0.002) | -15 to 0 |
| **Dementia case × 1^st^ period^2^** | .. | .. | .. | .. |
| **Dementia case × 2^nd^ period** | -0.18^†^ (-0.20, -0.16) | -16 to -2 | 0.16^§^ (0.04, 0.28) | -18 to -15 |
| **Dementia case × 3^rd^ period** | 0.52^‡^ (0.24, 0.79) | -19 to -16 | 0.43 (-0.34, 1.13) | -19 to -18 |
| **Fixed effects coefficients** | **Body mass index, kg/m^2^** | **Time periods^*^, years** | **Fasting PG, mmol/L** | **Time periods^*^, years** |
| **Intercept (baseline)** | 28.1^†^ (28.0, 28.1) | 0 | 8.29^†^ (8.23, 8.34) | 0 |
| **1^st^ period** | -0.36^†^ (-0.37, -0.35) | -11 to 0 | 0.17^†^ (0.16, 0.18) | -17 to 0 |
| **1^st^ period^2^** | -0.021^†^ (-0.022, -0.020) | -11 to 0 | 0.004^†^ (0.003, 0.005) | -17 to 0 |
| **2^nd^ period** | 0.05^†^ (0.04, 0.07) | -19 to -11 | -0.07 (-0.26, 0.13) | -19 to -17 |
| **3^rd^ period** |  | .. | .. | .. |
| **Dementia case** | -1.23^†^ (-1.33, -1.13) | 0 | 0.16^†^ (0.10, 0.23) | 0 |
| **Dementia case × 1^st^ period** | -0.11^†^ (-0.13, -0.10) | -11 to 0 | -0.02^§^ (-0.03, -0.003) | -17 to 0 |
| **Dementia case × 1^st^ period^2^** | -0.004^†^ (-0.005, -0.002) | -11 to 0 | -0.0015^‡^ (-0.0026, -0.0005) | -17 to 0 |
| **Dementia case × 2^nd^ period** | -0.023^†^ (-0.035, -0.011) | -19 to -11 | 0.06 (-0.16, 0.28) | -19 to -17 |
| **Dementia case × 3^rd^ period** | .. | .. | .. | .. |
| **Fixed effects coefficients** | **HbA1c, mmol/mol** | **Time periods^*^, years** | **Cholesterol, mmol/L** | **Time periods^*^, years** |
| **Intercept (baseline)** | 52.4^†^ (52.2, 52.6) | 0 | 4.65^†^ (4.64, 4.67) | 0 |
| **1^st^ period** | 0.28^†^ (0.22, 0.33) | -17 to 0 | -0.019^†^ (-0.022, -0.016) | -19 to 0 |
| **1^st^ period^2^** | 0.018^†^ (0.014, 0.023) | -17 to 0 | 0.005^†^ (0.005, 0.006) | -19 to 0 |
| **2^nd^ period** | 2.64^‡^ (1.06, 4.21) | -19 to -17 | .. | .. |
| **3^rd^ period** | .. | .. | .. | .. |
| **Dementia case** | 0.73^†^ (0.47, 0.98) | 0 | 0.071^†^ (0.053, 0.089) | 0 |
| **Dementia case × 1^st^ period** | -0.03 (-0.09, 0.04) | -17 to 0 | 0.004^§^ (0.0002, 0.008) | -19 to 0 |
| **Dementia case × 1^st^ period^2^** | -0.0002 (-0.005, 0.005) | -17 to 0 | 0.0005^†^ (0.0002, 0.0007) | -19 to 0 |
| **Dementia case × 2^nd^ period** | -1.98^‡^ (-3.21, -0.74) | -19 to -17 | .. | .. |
| **Dementia case × 3^rd^ period** | .. | .. | .. | .. |
|  | **HDL, mmol/L** | **Time periods^*^, years** | **LDL, mmol/L** | **Time periods^*^, years** |
| **Intercept (baseline)** | 1.487^†^ (1.482, 1.492) | 0 | 2.67^†^ (2.66, 2.69) | 0 |
| **1^st^ period** | 0.0023^†^ (0.0018, 0.0028) | -19 to 0 | 0.033^†^ (0.029, 0.037) | -15 to 0 |
| **1^st^ period^2^** | .. | .. | 0.008^†^ (0.008, 0.008) | -15 to 0 |
| **2^nd^ period** | .. | .. | -0.11^†^ (-0.15, -0.08) | -18 to -15 |
| **3^rd^ period** | .. | .. | -0.47^†^ (-0.71, -0.23) | -19 to -18 |
| **Dementia case** | 0.005 (-0.001, 0.011) | 0 | -0.04^‡^ (-0.06, -0.02) | 0 |
| **Dementia case × 1^st^ period** | -0.0004 (-0.0010, 0.0003) | -19 to 0 | -0.020^†^ (-0.025, -0.015) | -15 to 0 |
| **Dementia case × 1^st^ period^2^** | .. | .. | -0.001^†^ (-0.001, -0.001) | -15 to 0 |
| **Dementia case × 2^nd^ period** | .. | .. | -0.02 (-0.05, 0.01) | -18 to -15 |
| **Dementia case × 3^rd^ period** | .. | .. | -0.38^‡^ (-0.62, -0.14) | -19 to -18 |

Abbreviations: BP - blood pressure; PG, - plasma glucose; HbA1c - haemoglobin A1c; HDL - high-density lipoprotein; LDL - low-density lipoprotein.

Regression coefficients (95% confidence intervals) were derived from piecewise linear and non-linear growth curve models with random intercept and slope, and represent differences in annual changes in cardio-metabolic factors between dementia and non-dementia cases during distinct time periods over the 20-year follow-up. Time was expressed as years for up to three time periods. For HDL and total cholesterol, non-piecewise models were fitted (with a single time period).

^*^Study baseline is established as the date of diagnosis of dementia (dementia group) or last contact with healthcare (non-dementia group). Participants were then traced backwards in time. Time periods indicate the segmentation of the 20-year retrospective follow-up time ranging from -19 to 0 years (i.e., -19 refers to ‘19 years before year 0’), where the 1^st^ time period is the closest to baseline, followed by a 2^nd^ time period, and finally a 3^rd^ time period which is the furthest away from baseline.

Model is adjusted for age, sex, ethnicity, smoking status, Index of Multiple Deprivation quintiles, duration of diabetes, if ever prescribed insulin, stroke, and myocardial infarction. Patient ID and time (non-piecewise), or the time period closest to baseline (piecewise) was modelled as random effects.

Level of significance at ^†^*p*<0.001, unless otherwise indicated, ^‡^ *p*<0.01, ^§^ *p*<0.05.

# **Supplementary Table 14. Multilevel models for differences in trajectories of cardio-metabolic factors between people with type 2 diabetes with and without dementia over 20 years of retrospective follow-up: restricting to ≥10 years of follow-up**

| **Fixed effects coefficients** | **Systolic BP, mmHg** | **Time periods^*^, years** | **Diastolic BP, mmHg** | **Time periods^*^, years** |
| --- | --- | --- | --- | --- |
| **Intercept (baseline)** | 139.1^†^ (138.9, 139.4) | 0 | 73.7^†^ (73.6, 73.8) | 0 |
| **1^st^ period** | -0.46^†^ (-0.60, -0.32) | -2 to 0 | -0.61^†^ (-0.62, -0.59) | -15 to 0 |
| **1^st^ period^2^** | .. | .. | .. | .. |
| **2^nd^ period** | -0.64^†^ (-0.65, -0.62) | -16 to -2 | -1.17^†^ (-1.27, -1.07) | -18 to -15 |
| **3^rd^ period** | -1.02^†^ (-1.24, -0.81) | -19 to -16 | -0.75^‡^ (-1.28, -0.22) | -19 to -18 |
| **Dementia case** | -2.09^†^ (-2.45, -1.72) | 0 | 0.14 (-0.01, 0.28) | 0 |
| **Dementia case × 1^st^ period** | -0.48^†^ (-0.68, -0.29) | -2 to 0 | -0.007 (-0.02, 0.01) | -15 to 0 |
| **Dementia case × 1^st^ period^2^** | .. | .. | .. | .. |
| **Dementia case × 2^nd^ period** | -0.16^†^ (-0.18, -0.14) | -16 to -2 | 0.15^§^ (0.03, 0.27) | -18 to -15 |
| **Dementia case × 3^rd^ period** | 0.48^‡^ (0.21, 0.76) | -19 to -16 | 0.40 (-0.33, 1.13) | -19 to -18 |
| **Fixed effects coefficients** | **Body mass index, kg/m^2^** | **Time periods^*^, years** | **Fasting PG, mmol/L** | **Time periods^*^, years** |
| **Intercept (baseline)** | 28.1^†^ (28.0, 28.2) | 0 | 8.13^†^ (8.06, 8.20) | 0 |
| **1^st^ period** | -0.35^†^ (-0.37, -0.34) | -11 to 0 | 0.16^†^ (0.15, 0.18) | -17 to 0 |
| **1^st^ period^2^** | -0.021^†^ (-0.022, -0.020) | -11 to 0 | 0.005^†^ (0.004, 0.006) | -17 to 0 |
| **2^nd^ period** | 0.06^†^ (0.05, 0.07) | -19 to -11 | -0.05 (-0.23, 0.14) | -19 to -17 |
| **3^rd^ period** | .. | .. | .. | .. |
| **Dementia case** | -1.10^†^ (-1.23, -0.97) | 0 | 0.23^†^ (0.14, 0.31) | 0 |
| **Dementia case × 1^st^ period** | -0.11^†^ (-0.13, -0.10) | -11 to 0 | 0.006 (-0.01, 0.02) | -17 to 0 |
| **Dementia case × 1^st^ period^2^** | -0.004^†^ (-0.005, -0.002) | -11 to 0 | -0.0001 (-0.0012, 0.0010) | -17 to 0 |
| **Dementia case × 2^nd^ period** | -0.024^†^ (-0.036, -0.012) | -19 to -11 | 0.10 (-0.12, 0.31) | -19 to -17 |
| **Dementia case × 3^rd^ period** | .. | .. | .. | .. |
| **Fixed effects coefficients** | **HbA1c, mmol/mol** | **Time periods^*^, years** | **Cholesterol, mmol/L** | **Time periods^*^, years** |
| **Intercept (baseline)** | 52.0^†^ (51.7, 52.3) | 0 | 4.79^†^ (4.77, 4.81) | 0 |
| **1^st^ period** | 0.41^†^ (0.35, 0.47) | -17 to 0 | 0.001 (-0.002, 0.005) | -19 to 0 |
| **1^st^ period^2^** | 0.025^†^ (0.020, 0.030) | -17 to 0 | 0.006^†^ (0.006, 0.006) | -19 to 0 |
| **2^nd^ period** | 2.89^†^ (1.35, 4.42) | -19 to -17 | .. | .. |
| **3^rd^ period** | .. | .. | .. | .. |
| **Dementia case** | 1.10^†^ (0.78, 1.43) | 0 | 0.091^†^ (0.067, 0.114) | 0 |
| **Dementia case × 1^st^ period** | 0.05 (-0.03, 0.12) | -17 to 0 | 0.012^†^ (0.008, 0.017) | -19 to 0 |
| **Dementia case × 1^st^ period^2^** | 0.004 (-0.001, 0.009) | -17 to 0 | 0.0009^†^ (0.0006, 0.0012) | -19 to 0 |
| **Dementia case × 2^nd^ period** | -1.88^‡^ (-3.10, -0.67) | -19 to -17 | .. | .. |
| **Dementia case × 3^rd^ period** | .. | .. | .. | .. |
|  | **HDL, mmol/L** | **Time periods^*^, years** | **LDL, mmol/L** | **Time periods^*^, years** |
| **Intercept (baseline)** | 1.540^†^ (1.533, 1.547) | 0 | 2.82^†^ (2.80, 2.84) | 0 |
| **1^st^ period** | 0.0053^†^ (0.0047, 0.0058) | -19 to 0 | 0.052^†^ (0.047, 0.056) | -15 to 0 |
| **1^st^ period^2^** | .. | .. | 0.008^†^ (0.008, 0.009) | -15 to 0 |
| **2^nd^ period** | .. | .. | -0.10^†^ (-0.13, -0.06) | -18 to -15 |
| **3^rd^ period** | .. | .. | -0.48^†^ (-0.73, -0.24) | -19 to -18 |
| **Dementia case** | 0.011 (0.002, 0.019) | 0 | -0.03^§^ (-0.05, -0.00) | 0 |
| **Dementia case × 1^st^ period** | 0.00002 (-0.0006, 0.0007) | -19 to 0 | -0.014^†^ (-0.019, -0.008) | -15 to 0 |
| **Dementia case × 1^st^ period^2^** | .. | .. | -0.001^‡^ (-0.001, 0.000) | -15 to 0 |
| **Dementia case × 2^nd^ period** | .. | .. | -0.02 (-0.05, 0.01) | -18 to -15 |
| **Dementia case × 3^rd^ period** | .. | .. | -0.38^‡^ (-0.62, -0.14) | -19 to -18 |

Abbreviations: BP - blood pressure; PG, - plasma glucose; HbA1c - haemoglobin A1c; HDL - high-density lipoprotein; LDL - low-density lipoprotein.

Regression coefficients (95% confidence intervals) were derived from piecewise linear and non-linear growth curve models with random intercept and slope, and represent differences in annual changes in cardio-metabolic factors between dementia and non-dementia cases during distinct time periods over the 20-year follow-up. Time was expressed as years for up to three time periods. For HDL and total cholesterol, non-piecewise models were fitted (with a single time period).

^*^Study baseline is established as the date of diagnosis of dementia (dementia group) or last contact with healthcare (non-dementia group). Participants were then traced backwards in time. Time periods indicate the segmentation of the 20-year retrospective follow-up time ranging from -19 to 0 years (i.e., -19 refers to ‘19 years before year 0’), where the 1^st^ time period is the closest to baseline, followed by a 2^nd^ time period, and finally a 3^rd^ time period which is the furthest away from baseline.

Model is adjusted for age, sex, ethnicity, smoking status, Index of Multiple Deprivation quintiles, duration of diabetes, if ever prescribed insulin, stroke, and myocardial infarction. Patient ID and time (non-piecewise), or the time period closest to baseline (piecewise) was modelled as random effects.

Level of significance at ^†^*p*<0.001, unless otherwise indicated, ^‡^ *p*<0.01, ^§^ *p*<0.05.

# **Supplementary Table 15.** **Multilevel models for differences in trajectories of cardio-metabolic factors between people with type 2 diabetes with and without dementia over 20 years of retrospective follow-up: additionally adjusting for time-varying anti-hypertensive, anti-hyperglycaemia, anti-lipid, and anti-platelet medication**

| **Fixed effects coefficients** | **Systolic BP, mmHg** | **Time periods^*^, years** | **Diastolic BP, mmHg** | **Time periods^*^, years** |
| --- | --- | --- | --- | --- |
| **Intercept (baseline)** | 139.4^†^ (139.2, 139.6) | 0 | 75.1^†^ (75.0, 75.2) | 0 |
| **1^st^ period** | -1.10^†^ (-1.22, -0.99) | -2 to 0 | -0.54^†^ (-0.55, -0.52) | -15 to 0 |
| **1^st^ period^2^** | .. | .. | .. | .. |
| **2^nd^ period** | -0.52^†^ (-0.53, -0.50) | -16 to -2 | -1.05^†^ (-1.15, -0.95) | -18 to -15 |
| **3^rd^ period** | -1.07^†^ (-1.28, -0.86) | -19 to -16 | -0.79^‡^ (-1.32, -0.25) | -19 to -18 |
| **Dementia case** | -1.88^†^ (-2.15, -1.61) | 0 | 0.20^‡^ (-0.08, 0.31) | 0 |
| **Dementia case × 1^st^ period** | -0.43^†^ (-0.58, -0.29) | -2 to 0 | 0.004 (-0.01, 0.02) | -15 to 0 |
| **Dementia case × 1^st^ period^2^** | .. | .. | .. | .. |
| **Dementia case × 2^nd^ period** | -0.15^†^ (-0.17, -0.13) | -16 to -2 | 0.13^§^ (0.01, 0.25) | -18 to -15 |
| **Dementia case × 3^rd^ period** | 0.49^†^ (0.21, 0.76) | -19 to -16 | 0.44 (-0.29, 1.17) | -19 to -18 |
| **Fixed effects coefficients** | **Body mass index, kg/m^2^** | **Time periods^*^, years** | **Fasting PG, mmol/L** | **Time periods^*^, years** |
| **Intercept (baseline)** | 28.1^†^ (28.0, 28.2) | 0 | 7.68^†^ (7.63, 7.74) | 0 |
| **1^st^ period** | -0.35^†^ (-0.36, -0.34) | -11 to 0 | 0.10^†^ (0.09, 0.11) | -17 to 0 |
| **1^st^ period^2^** | -0.021^†^ (-0.022, -0.020) | -11 to 0 | 0.002^†^ (0.001, 0.003) | -17 to 0 |
| **2^nd^ period** | 0.05^†^ (0.04, 0.06) | -19 to -11 | -0.05 (-0.23, 0.14) | -19 to -17 |
| **3^rd^ period** | .. | .. | .. | .. |
| **Dementia case** | -1.23^†^ (-1.33, -1.13) | 0 | 0.13^†^ (0.07, 0.19) | 0 |
| **Dementia case × 1^st^ period** | -0.12^†^ (-0.13, -0.10) | -11 to 0 | -0.014 (-0.029, 0.001) | -17 to 0 |
| **Dementia case × 1^st^ period^2^** | -0.004^†^ (-0.005, -0.002) | -11 to 0 | -0.0011^‡^ (-0.0021, -0.0001) | -17 to 0 |
| **Dementia case × 2^nd^ period** | -0.02^†^ (-0.03, -0.01) | -19 to -11 | 0.03 (-0.19, 0.25) | -19 to -17 |
| **Dementia case × 3^rd^ period** | .. | .. | .. | .. |
| **Fixed effects coefficients** | **HbA1c, mmol/mol** | **Time periods^*^, years** | **Cholesterol, mmol/L** | **Time periods^*^, years** |
| **Intercept (baseline)** | 51.1^†^ (50.9, 51.3) | 0 | 5.30^†^ (5.28, 5.31) | 0 |
| **1^st^ period** | 0.18^†^ (0.13, 0.23) | -17 to 0 | -0.012^†^ (-0.015, -0.009) | -19 to 0 |
| **1^st^ period^2^** | 0.022^†^ (0.018, 0.027) | -17 to 0 | 0.0031^†^ (0.0029, 0.0033) | -19 to 0 |
| **2^nd^ period** | 2.56^‡^ (0.99, 4.12) | -19 to -17 | .. | .. |
| **3^rd^ period** | .. | .. | .. | .. |
| **Dementia case** | 0.53^†^ (0.28, 0.77) | 0 | 0.062^†^ (0.045, 0.079) | 0 |
| **Dementia case × 1^st^ period** | -0.01 (-0.08, 0.05) | -17 to 0 | 0.0039^§^ (0.0002, 0.0077) | -19 to 0 |
| **Dementia case × 1^st^ period^2^** | 0.001 (-0.004, 0.006) | -17 to 0 | 0.0003^§^ (0.0001, 0.0006) | -19 to 0 |
| **Dementia case × 2^nd^ period** | -2.26^†^ (-3.49, -1.03) | -19 to -17 | .. | .. |
| **Dementia case × 3^rd^ period** | .. | .. | .. | .. |
|  | **HDL, mmol/L** | **Time periods^*^, years** | **LDL, mmol/L** | **Time periods^*^, years** |
| **Intercept (baseline)** | 1.528^†^ (1.523, 1.534) | 0 | 3.24^†^ (3.22, 3.26) | 0 |
| **1^st^ period** | 0.005^†^ (0.004, 0.005) | -19 to 0 | 0.038^†^ (0.035, 0.042) | -15 to 0 |
| **1^st^ period^2^** | .. | .. | 0.0059^†^ (0.0056, 0.0062) | -15 to 0 |
| **2^nd^ period** | .. | .. | -0.05^‡^ (-0.08, -0.01) | -18 to -15 |
| **3^rd^ period** | .. | .. | -0.47^†^ (-0.71, -0.23) | -19 to -18 |
| **Dementia case** | 0.007^‡^ (-0.001, 0.013) | 0 | -0.04^†^ (-0.06, -0.02) | 0 |
| **Dementia case × 1^st^ period** | -0.0001 (-0.0007, 0.0005) | -19 to 0 | -0.019^†^ (-0.023, -0.014) | -15 to 0 |
| **Dementia case × 1^st^ period^2^** | .. | .. | -0.0010^†^ (-0.0013, -0.0007) | -15 to 0 |
| **Dementia case × 2^nd^ period** | .. | .. | -0.02 (-0.05, 0.01) | -18 to -15 |
| **Dementia case × 3^rd^ period** | .. | .. | -0.30^‡^ (-0.53, -0.06) | -19 to -18 |

Abbreviations: BP - blood pressure; PG, - plasma glucose; HbA1c - haemoglobin A1c; HDL - high-density lipoprotein; LDL - low-density lipoprotein.

Regression coefficients (95% confidence intervals) were derived from piecewise linear and non-linear growth curve models with random intercept and slope, and represent differences in annual changes in cardio-metabolic factors between dementia and non-dementia cases during distinct time periods over the 20-year follow-up. Time was expressed as years for up to three time periods. For HDL and total cholesterol, non-piecewise models were fitted (with a single time period).

^*^Study baseline is established as the date of diagnosis of dementia (dementia group) or last contact with healthcare (non-dementia group). Participants were then traced backwards in time. Time periods indicate the segmentation of the 20-year retrospective follow-up time ranging from -19 to 0 years (i.e., -19 refers to ‘19 years before year 0’), where the 1^st^ time period is the closest to baseline, followed by a 2^nd^ time period, and finally a 3^rd^ time period which is the furthest away from baseline.

Model is adjusted for age, sex, ethnicity, smoking status, Index of Multiple Deprivation quintiles, duration of diabetes, if ever prescribed insulin, stroke, and myocardial infarction. Patient ID and time (non-piecewise), or the time period closest to baseline (piecewise) was modelled as random effects.

Level of significance at ^†^*p*<0.001, unless otherwise indicated, ^‡^ *p*<0.01, ^§^ *p*<0.05.

# **Supplementary Table 16.** **Characteristics of study participants with type 2 diabetes in the Clinical Research Practice Datalink at the time of dementia diagnosis (dementia group) or last contact with healthcare (non-dementia group) between 1999 and 2018 in England: a case-control approach matched without replacement by age (±3 years) and sex**

|  | **TOTAL**  **(n=70,638)** | **DEMENTIA (n=23,546)** | **NON-DEMENTIA (n=47,092)** | **p-value^*^** |
| --- | --- | --- | --- | --- |
| Age, years, mean (SD) | 80.5 (8.4) | 82.2 (8.1) | 79.6 (8.4) | <0.001 |
| Sex, n (%) |  |  |  |  |
| Male | 30,471 (43.1) | 10,157 (43.1) | 20,314 (43.1) | <0.001 |
| Female | 40,167 (56.7) | 13,389 (56.9) | 26,778 (56.9) |  |
| Ethnicity, n (%) |  |  |  |  |
| White | 62,897 (89.0) | 21,606 (91.8) | 41,291 (87.7) | <0.001 |
| Non-White | 4,461 (6.3) | 1,392 (5.9) | 3,069 (6.5) |  |
| Unknown/missing | 3,280 (4.6) | 548 (2.3) | 2,732 (5.8) |  |
| Smoking status, n (%) |  |  |  |  |
| Non-smoker | 29,214 (41.4) | 9,968 (42.3) | 19,246 (40.9) | <0.001 |
| Smoker | 17,435 (24.7) | 5,407 (23.0) | 12,028 (25.5) |  |
| Ex-smoker | 23,989 (34.0) | 8,171 (34.7) | 15,818 (33.6) |  |
| Index of Multiple Deprivation (IMD) quintiles, n (%) |  |  |  |  |
| 1^st^ quintile (least deprived) | 10,257 (14.5) | 3,264 (13.8) | 6,993 (14.8) | 0.190 |
| 2 | 13,813 (19.6) | 4,480 (19.0) | 9,333 (19.8) |  |
| 3 | 13,805 (19.5) | 4,520 (19.2) | 9,285 (19.7) |  |
| 4 | 15,626 (22.1) | 5,367 (22.8) | 10,259 (21.8) |  |
| 5^th^ quintile (most deprived) | 17,137 (24.3) | 5,915 (25.1) | 11,222 (23.8) |  |
| Diabetes duration, years, mean (SD) | 7.4 (5.8) | 8.2 (6.0) | 7.1 (5.6) | <0.001 |
| Insulin, ever prescribed, n (%) | 7,171 (10.2) | 2,558 (10.9) | 4,613 (9.8) | <0.001 |
| Number of co-morbidities, median (IQR)^†^ | 2 (1-3) | 2 (1-3) | 2 (1-3) | <0.001 |
| Anti-hyperglycaemic, ever prescribed, n (%) | 39,948 (56.6) | 13,341 (56.7) | 26,607 (56.5) | 0.687 |
| Anti-hypertensive, ever prescribed, n (%) | 54,738 (77.5) | 17,918 (76.1) | 36,820 (78.2) | <0.001 |
| Anti-lipid, ever prescribed, n (%) | 45,151 (63.9) | 14,883 (63.2) | 30,268 (64.3) | 0.005 |
| Anti-platelet, ever prescribed, n (%) | 33,459 (47.4) | 11,733 (49.8) | 21,726 (46.1) | <0.001 |
| Retrospective follow-up time, years, mean (SD) | 10.0 (5.8) | 9.6 (5.8) | 10.3 (5.9) | <0.001 |

**^*^**Comparisons were made between dementia and non-dementia groups, with p-value obtained through two-sample independent t-test for continuous variables and chi-squared test for categorical variables.

^†^Comorbidities include stroke, acute myocardial infarction, peripheral artery disease, atrial fibrillation, heart failure, asthma, chronic obstructive pulmonary disease, cancer, chronic kidney disease, rheumatoid arthritis, Parkinson’s disease, and clinical depression.

# **Supplementary Table 17. Multilevel models for differences in trajectories of cardio-metabolic factors between people with type 2 diabetes with and without dementia over 20 years of retrospective follow-up: case-control approach matched by age (±3 years) and sex**

| **Fixed effects coefficients** | **Systolic BP, mmHg** | **Time periods^*^, years** | **Diastolic BP, mmHg** | **Time periods^*^, years** |
| --- | --- | --- | --- | --- |
| **Intercept (baseline)** | 138.5^†^ (138.1, 138.9) | 0 | 74.2^†^ (74.0, 74.3) | 0 |
| **1^st^ period** | -1.39^†^ (-1.60, -1.19) | -2 to 0 | -0.64^†^ (-0.66, -0.61) | -15 to 0 |
| **1^st^ period^2^** | .. | .. | .. | .. |
| **2^nd^ period** | -0.65^†^ (-0.68, -0.60) | -16 to -2 | -1.16^†^ (-1.39, -0.94) | -18 to -15 |
| **3^rd^ period** | -0.51^§^ (-1.03, 0.01) | -19 to -16 | -0.57 (-1.54, 0.39) | -19 to -18 |
| **Dementia case** | -2.01^†^ (-2.32, -1.69) | 0 | -0.06 (-0.19, 0.07) | 0 |
| **Dementia case × 1^st^ period** | -0.41^†^ (-0.58, -0.24) | -2 to 0 | -0.008 (-0.02, 0.01) | -15 to 0 |
| **Dementia case × 1^st^ period^2^** | .. | .. | .. | .. |
| **Dementia case × 2^nd^ period** | -0.15^†^ (-0.19, -0.12) | -16 to -2 | 0.15 (-0.02, 0.31) | -18 to -15 |
| **Dementia case × 3^rd^ period** | 0.27 (0.17, 0.72) | -19 to -16 | 0.02 (-0.87, 0.90) | -19 to -18 |
| **Fixed effects coefficients** | **Body mass index, kg/m^2^** | **Time periods^*^, years** | **Fasting PG, mmol/L** | **Time periods^*^, years** |
| **Intercept (baseline)** | 27.9^†^ (27.8, 28.1) | 0 | 8.56^†^ (8.45, 8.67) | 0 |
| **1^st^ period** | -0.33^†^ (-0.36, -0.31) | -11 to 0 | 0.20^†^ (0.17, 0.23) | -17 to 0 |
| **1^st^ period^2^** | -0.019^†^ (-0.022, -0.017) | -11 to 0 | 0.007^†^ (0.005, 0.009) | -17 to 0 |
| **2^nd^ period** | 0.06^†^ (0.03, 0.09) | -19 to -11 | -0.003 (-0.35, 0.34) | -19 to -17 |
| **3^rd^ period** | .. | .. | .. | .. |
| **Dementia case** | -1.15^†^ (-1.25, -1.04) | 0 | 0.10^§^ (0.02, 0.18) | 0 |
| **Dementia case × 1^st^ period** | -0.12^†^ (-0.14, -0.09) | -11 to 0 | -0.03^‡^ (-0.05, -0.007) | -17 to 0 |
| **Dementia case × 1^st^ period^2^** | -0.004^†^ (-0.006, -0.002) | -11 to 0 | -0.002^‡^ (-0.004, -0.0008) | -17 to 0 |
| **Dementia case × 2^nd^ period** | -0.022 (-0.045, -0.001) | -19 to -11 | 0.03 (-0.29, 0.34) | -19 to -17 |
| **Dementia case × 3^rd^ period** | .. | .. | .. | .. |
| **Fixed effects coefficients** | **HbA1c, mmol/mol** | **Time periods^*^, years** | **Cholesterol, mmol/L** | **Time periods^*^, years** |
| **Intercept (baseline)** | 53.3^†^ (52.9, 53.7) | 0 | 4.79^†^ (4.76, 4.82) | 0 |
| **1^st^ period** | 0.31^†^ (0.19, 0.44) | -17 to 0 | -0.008^§^ (-0.016, -0.0004) | -19 to 0 |
| **1^st^ period^2^** | 0.036^†^ (0.024, 0.048) | -17 to 0 | 0.007^†^ (0.006, 0.007) | -19 to 0 |
| **2^nd^ period** | 3.24 (-0.80, 7.28) | -19 to -17 | .. | .. |
| **3^rd^ period** | .. | .. | .. | .. |
| **Dementia case** | 0.66^†^ (0.36, 0.96) | 0 | 0.052^†^ (0.031, 0.073) | 0 |
| **Dementia case × 1^st^ period** | 0.02 (-0.08, 0.12) | -17 to 0 | 0.009^‡^ (0.003, 0.015) | -19 to 0 |
| **Dementia case × 1^st^ period^2^** | -0.0029 (-0.006, 0.011) | -17 to 0 | 0.0005^§^ (0.0001, 0.0010) | -19 to 0 |
| **Dementia case × 2^nd^ period** | -2.14 (-4.67, 0.39) | -19 to -17 | .. | .. |
| **Dementia case × 3^rd^ period** | .. | .. | .. | .. |
|  | **HDL, mmol/L** | **Time periods^*^, years** | **LDL, mmol/L** | **Time periods^*^, years** |
| **Intercept (baseline)** | 1.504^†^ (1.494, 1.513) | 0 | 2.75^†^ (2.72, 2.78) | 0 |
| **1^st^ period** | 0.0030^†^ (0.0021, 0.0039) | -19 to 0 | 0.048^†^ (0.038, 0.057) | -15 to 0 |
| **1^st^ period^2^** | .. | .. | 0.010^†^ (0.009, 0.011) | -15 to 0 |
| **2^nd^ period** | .. | .. | -0.07 (-0.16, 0.02) | -18 to -15 |
| **3^rd^ period** | .. | .. | -1.12^†^ (-1.64, -0.60) | -19 to -18 |
| **Dementia case** | 0.006 (-0.002, 0.013) | 0 | -0.04^†^ (-0.06, -0.02) | 0 |
| **Dementia case × 1^st^ period** | -0.0001 (-0.0008, 0.0006) | -19 to 0 | -0.016^†^ (-0.023, -0.009) | -15 to 0 |
| **Dementia case × 1^st^ period^2^** | .. | .. | -0.001^†^ (-0.002, -0.0005) | -15 to 0 |
| **Dementia case × 2^nd^ period** | .. | .. | -0.02 (-0.07, 0.04) | -18 to -15 |
| **Dementia case × 3^rd^ period** | .. | .. | -0.29 (-0.73, 0.14) | -19 to -18 |

Abbreviations: BP - blood pressure; PG, - plasma glucose; HbA1c - haemoglobin A1c; HDL - high-density lipoprotein; LDL - low-density lipoprotein.

Regression coefficients (95% confidence intervals) were derived from piecewise linear and non-linear growth curve models with random intercept and slope, and represent differences in annual changes in cardio-metabolic factors between dementia and non-dementia cases during distinct time periods over the 20-year follow-up. Time was expressed as years for up to three time periods. For HDL and total cholesterol, non-piecewise models were fitted (with a single time period).

^*^Study baseline is established as the date of diagnosis of dementia (dementia group) or last contact with healthcare (non-dementia group). Participants were then traced backwards in time. Time periods indicate the segmentation of the 20-year retrospective follow-up time ranging from -19 to 0 years (i.e., -19 refers to ‘19 years before year 0’), where the 1^st^ time period is the closest to baseline, followed by a 2^nd^ time period, and finally a 3^rd^ time period which is the furthest away from baseline.

Model is adjusted for age, sex, ethnicity, smoking status, Index of Multiple Deprivation quintiles, duration of diabetes, if ever prescribed insulin, stroke, and myocardial infarction. Patient ID and time (non-piecewise), or the time period closest to baseline (piecewise) was modelled as random effects.

Level of significance at ^†^*p*<0.001, unless otherwise indicated, ^‡^ *p*<0.01, ^§^ *p*<0.05.

# **Supplementary Table 18. Annual differences (95% confidence interval) in levels of systolic blood pressure, diastolic blood pressure, body mass index, fasting plasma glucose, and haemoglobin A1c for participants with dementia relative to participants without dementia: case-control approach matched by age (±3 years) and sex**

| **Year^*^** | **Systolic blood pressure** | | **Diastolic blood pressure** | | **Body mass index** | | **Fasting plasma glucose** | | **Haemoglobin A1c** | |
| --- | --- | --- | --- | --- | --- | --- | --- | --- | --- | --- |
|  | **Difference in mmHg/year** | **p-value**^†^ | **Difference in mmHg/year** | **p-value**^†^ | **Difference in kg/m^2^** | **p-value**^†^ | **Difference in mmol/L** | **p-value**^†^ | **Difference in mmol/mol** | **p-value**^†^ |
| **-19** | 0.15 (-1.18, 1.48) | 0.825 | -0.39 (-1.28, 0.50) | 0.388 | -0.14 (-0.33, 0.05) | 0.138 | -0.12 (0.71, 0.51) | 0.710 | 5.47 (0.38, 10.55) | 0.035 |
| **-18** | 0.42 (-0.49, 1.34) | 0.363 | -0.38 (-0.89, 0.13) | 0.149 | -0.16 (-0.33, 0.01) | 0.057 | -0.09 (0.59, 0.24) | 0.593 | 3.33 (0.63, 6.03) | 0.016 |
| **-17** | 0.70 (0.15, 1.24) | 0.012 | -0.23 (-0.59, 0.13) | 0.211 | -0.19 (-0.34, -0.03) | 0.016 | -0.06 (0.45, 0.10) | 0.450 | 1.19 (-0.03, 2.41) | 0.056 |
| **-16** | 0.97 (0.58, 1.37) | <0.001 | -0.08 (-0.32, 0.15) | 0.481 | -0.21 (-0.34, -0.07) | 0.003 | -0.02 (0.82, 0.12) | 0.818 | 1.11 (0.08, 2.15) | 0.036 |
| **-15** | 0.82 (0.45, 1.18) | <0.001 | 0.06 (-0.13, 0.25) | 0.535 | -0.23 (-0.35, -0.11) | <0.001 | 0.03 (0.64, 0.14) | 0.642 | 1.04 (0.17, 1.91) | 0.019 |
| **-14** | 0.66 (0.33, 1.00) | <0.001 | 0.05 (-0.13, 0.23) | 0.560 | -0.25 (-0.36, -0.14) | <0.001 | 0.06 (0.17, 0.15) | 0.167 | 0.97 (0.25, 1.70) | 0.008 |
| **-13** | 0.51 (0.20, 0.82) | 0.001 | 0.05 (-0.12, 0.21) | 0.592 | -0.27 (-0.38, -0.17) | <0.001 | 0.10 (0.01, 0.17) | 0.011 | 0.91 (0.32, 1.51) | 0.003 |
| **-12** | 0.36 (0.07, 0.64) | 0.015 | 0.04 (-0.12, 0.19) | 0.631 | -0.30 (-0.40, -0.19) | <0.001 | 0.13 (0.00, 0.19) | <0.001 | 0.86 (0.37, 1.35) | 0.001 |
| **-11** | 0.20 (-0.06, 0.47) | 0.134 | 0.03 (-0.11, 0.17) | 0.680 | -0.32 (-0.43, -0.21) | <0.001 | 0.15 (0.00, 0.20) | <0.001 | 0.81 (0.41, 1.22) | <0.001 |
| **-10** | 0.05 (-0.20, 0.29) | 0.702 | 0.02 (-0.11, 0.15) | 0.742 | -0.35 (-0.45, -0.26) | <0.001 | 0.17 (0.00, 0.22) | <0.001 | 0.77 (0.43, 1.11) | <0.001 |
| **-9** | -0.11 (-0.33, 0.12) | 0.359 | 0.01 (-0.11, 0.14) | 0.820 | -0.40 (-0.49, -0.30) | <0.001 | 0.18 (0.00, 0.23) | <0.001 | 0.73 (0.43, 1.03) | <0.001 |
| **-8** | -0.26 (-0.48, -0.05) | 0.017 | 0.01 (-0.11, 0.12) | 0.913 | -0.45 (-0.54, -0.36) | <0.001 | 0.19 (0.00, 0.24) | <0.001 | 0.70 (0.43, 0.97) | <0.001 |
| **-7** | -0.41 (-0.62, -0.21) | <0.001 | 0.00 (-0.11, 0.11) | 0.977 | -0.51 (-0.60, -0.42) | <0.001 | 0.20 (0.00, 0.25) | <0.001 | 0.68 (0.42, 0.93) | <0.001 |
| **-6** | -0.57 (-0.77, -0.37) | <0.001 | -0.01 (-0.11, 0.09) | 0.859 | -0.58 (-0.67, -0.49) | <0.001 | 0.20 (0.00, 0.25) | <0.001 | 0.66 (0.42, 0.90) | <0.001 |
| **-5** | -0.72 (-0.93, -0.52) | <0.001 | -0.02 (-0.12, 0.08) | 0.741 | -0.65 (-0.75, -0.56) | <0.001 | 0.19 (0.00, 0.24) | <0.001 | 0.64 (0.41, 0.87) | <0.001 |
| **-4** | -0.88 (-1.09, -0.66) | <0.001 | -0.02 (-0.13, 0.08) | 0.634 | -0.74 (-0.83, -0.65) | <0.001 | 0.18 (0.00, 0.23) | <0.001 | 0.64 (0.42, 0.86) | <0.001 |
| **-3** | -1.03 (-1.26, -0.80) | <0.001 | -0.03 (-0.14, 0.07) | 0.547 | -0.83 (-0.92, -0.74) | <0.001 | 0.17 (0.00, 0.22) | <0.001 | 0.63 (0.42, 0.85) | <0.001 |
| **-2** | -1.19 (-1.43, -0.94) | <0.001 | -0.04 (-0.15, 0.07) | 0.480 | -0.93 (-1.02, -0.84) | <0.001 | 0.15 (0.00, 0.21) | <0.001 | 0.64 (0.42, 0.85) | <0.001 |
| **-1** | -1.60 (-1.82, -1.37) | <0.001 | -0.05 (-0.17, 0.07) | 0.432 | -1.03 (-1.13, -0.94) | <0.001 | 0.13 (0.00, 0.20) | <0.001 | 0.65 (0.40, 0.89) | <0.001 |
| **0** | -2.01 (-2.32, -1.69) | <0.001 | -0.06 (-0.19, 0.07) | 0.397 | -1.15 (-1.25, -1.04) | <0.001 | 0.10 (0.01, 0.18) | 0.011 | 0.66 (0.36, 0.96) | <0.001 |

^*^Study baseline (year 0) is established as the date of diagnosis of dementia (dementia group) or last contact with healthcare (non-dementia group). Participants were then traced backwards in time. As such, -19 year refers to ‘19 years before year 0’.

^†^ Differences presented are for the dementia group relative to the non-dementia group, with p-value obtained through the Wald-test.

# **Supplementary Table 19. Annual differences (95% confidence interval) in levels of cholesterol, low-density lipoprotein, and high-density lipoprotein for participants with dementia relative to participants without dementia: case-control approach matched by age (±3 years) and sex**

| **Year** | **Cholesterol** | | **Low-density lipoprotein** | | **High-density lipoprotein** | |
| --- | --- | --- | --- | --- | --- | --- |
|  | **Difference in mmol/L** | **p-value^*^** | **Difference in mmol/L** | **p-value^*^** | **Difference in mmol/L** | **p-value^*^** |
| **-19** | 0.074 (-0.004, 0.151) | 0.063 | 0.30 (-0.12, 0.72) | 0.158 | 0.007 (-0.005, 0.018) | 0.245 |
| **-18** | 0.063 (-0.004, 0.130) | 0.065 | 0.01 (-0.15, 0.17) | 0.907 | 0.007 (-0.004, 0.017) | 0.225 |
| **-17** | 0.054 (-0.004, 0.111) | 0.067 | -0.01 (-0.12, 0.11) | 0.897 | 0.007 (-0.004, 0.017) | 0.204 |
| **-16** | 0.045 (-0.004, 0.094) | 0.070 | -0.02 (-0.09, 0.04) | 0.485 | 0.006 (-0.003, 0.016) | 0.184 |
| **-15** | 0.038 (-0.003, 0.079) | 0.073 | -0.04 (-0.09, 0.01) | 0.114 | 0.006 (-0.003, 0.016) | 0.164 |
| **-14** | 0.031 (-0.003, 0.066) | 0.076 | -0.03 (-0.07, 0.02) | 0.216 | 0.006 (-0.002, 0.015) | 0.145 |
| **-13** | 0.026 (-0.003, 0.056) | 0.081 | -0.01 (-0.05, 0.02) | 0.424 | 0.006 (-0.002, 0.014) | 0.127 |
| **-12** | 0.022 (-0.003, 0.047) | 0.089 | 0.00 (-0.03, 0.03) | 0.795 | 0.006 (-0.001, 0.014) | 0.110 |
| **-11** | 0.019 (-0.003, 0.041) | 0.098 | 0.00 (-0.02, 0.03) | 0.715 | 0.006 (-0.001, 0.013) | 0.096 |
| **-10** | 0.016 (-0.003, 0.036) | 0.105 | 0.01 (-0.01, 0.03) | 0.312 | 0.006 (-0.001, 0.013) | 0.084 |
| **-9** | 0.015 (-0.003, 0.034) | 0.106 | 0.02 (0.00, 0.03) | 0.119 | 0.006 (-0.001, 0.013) | 0.074 |
| **-8** | 0.015 (-0.003, 0.033) | 0.092 | 0.02 (0.00, 0.04) | 0.056 | 0.006 (0.000, 0.012) | 0.067 |
| **-7** | 0.016 (-0.001, 0.033) | 0.064 | 0.02 (0.00, 0.04) | 0.045 | 0.006 (0.000, 0.012) | 0.064 |
| **-6** | 0.018 (0.002, 0.035) | 0.032 | 0.02 (0.00, 0.03) | 0.065 | 0.006 (0.000, 0.012) | 0.063 |
| **-5** | 0.021 (0.005, 0.037) | 0.010 | 0.01 (0.00, 0.03) | 0.155 | 0.006 (0.000, 0.012) | 0.066 |
| **-4** | 0.025 (0.010, 0.041) | 0.002 | 0.01 (-0.01, 0.02) | 0.485 | 0.006 (-0.001, 0.012) | 0.072 |
| **-3** | 0.030 (0.015, 0.046) | 0.000 | 0.00 (-0.02, 0.01) | 0.737 | 0.006 (-0.001, 0.012) | 0.082 |
| **-2** | 0.036 (0.020, 0.053) | 0.000 | -0.01 (-0.03, 0.00) | 0.117 | 0.006 (-0.001, 0.012) | 0.096 |
| **-1** | 0.044 (0.026, 0.062) | 0.000 | -0.03 (-0.04, -0.01) | 0.007 | 0.006 (-0.001, 0.013) | 0.114 |
| **0** | 0.052 (0.031, 0.073) | 0.000 | -0.04 (-0.06, -0.02) | 0.000 | 0.006 (-0.002, 0.013) | 0.136 |

^*^Study baseline (year 0) is established as the date of diagnosis of dementia (dementia group) or last contact with healthcare (non-dementia group). Participants were then traced backwards in time. As such, -19 year refers to ‘19 years before year 0’.

^†^ Differences presented are for the dementia group relative to the non-dementia group, with p-value obtained through the Wald-test.





**Supplementary Figure 6. Trajectories of (A) systolic blood pressure, (B) diastolic blood pressure, (C) body mass index, (D) fasting plasma glucose, (E) haemaglobin A1c, (F) cholesterol, (G) high-density lipoprotein, (H) low-density lipoprotein over 20 years of retrospective follow-up: a case-control approach.** Year 0 (zero time point) represents either the date of diagnosis of dementia (dementia group) or last contact with healthcare (non-dementia group). Participants were matched without replacement, with two controls per person with type 2 diabetes by age at baseline (±3 years) and exactly by sex. Participants were then traced backwards in time and were allowed to enter the cohort at any time conditional on a diabetes diagnosis within their retrospective follow-up duration. Estimations are based on piecewise linear (systolic and diastolic blood pressure) and non-linear (body mass index) growth curve models, including up to three time periods modelled as linear or non-linear splines, dementia status, and interactions between dementia status and time. Coloured solid lines represent point estimates for each group, coloured dotted lines for 95% confidence intervals for said estimations, while modelled time periods are indicted by grey vertical dotted lines. Three time periods ranging from year 0 to -2, -2 to -16, -16 to -19 (from right to left) were defined for systolic blood pressure; from year 0 to -15, -15 to -18, -18 to -19 for diastolic blood pressure; and two time periods ranging from year 0 to -11 and -11 to -19 were defined for body mass index. The two time periods ranging from year 0 to -17 and -17 to -19 (from right to left) were defined for fasting plasma glucose and haemoglobin A1c. Both total cholesterol and high-density lipoprotein considered time as a single period from 0 to -19 (from right to left), while three time periods ranging from year 0 to -15, -15 to -18, -18 to -19 were defined for low-density lipoprotein. Retrospective trajectories are adjusted for baseline covariates including age, sex, ethnicity, smoking status, Index of Multiple Deprivation quintiles, duration of diabetes and their interactions with time, and time-varying covariates including insulin prescription and number of comorbid conditions. Tables underneath each graph report the number of measurements for dementia and non-dementia group at each stated retrospective follow-up year.

# **References**

1. National Statistics: English indices of deprivation 2015 [article online], 2015. Available from <https://www.gov.uk/government/statistics/english-indices-of-deprivation-2015>. Accessed 03 Feb 2022

2. Herrett E, Gallagher AM, Bhaskaran K, Forbes H, Mathur R, van Staa T, Smeeth L. Data Resource Profile: Clinical Practice Research Datalink (CPRD). International Journal of Epidemiology 2015;44:827-836

3. Guidance on Dementia Coding [article online], 2012. Available from <https://dementiapartnerships.com/wp-content/uploads/sites/2/nhs-london-read-code-guidance.pdf>. Accessed 18 February 2021 2021
